# Quantifying the Influences of Epoxide Binding in Epoxide/CO_2_ Ring Opening Copolymerization Catalysis

**DOI:** 10.1021/jacs.5c09088

**Published:** 2026-02-11

**Authors:** Katharina H. S. Eisenhardt, Francesca Fiorentini, Jae Elise L. Payong, Ute L. Petri, Antoine Buchard, Jenny Yang, Charlotte K. Williams

**Affiliations:** † Department of Chemistry, Chemistry Research Laboratory, 6396 University of Oxford, Oxford OX1 3TA, U.K.; ‡ Department of Chemistry, 8788 University of California, Irvine, Irvine, California 92697, United States; § Department of Chemistry, Green Chemistry Centre of Excellence, 8748 University of York, York YO10 5DD, U.K.

## Abstract

Understanding and predicting the effect of epoxide structure on the rate of polymerization in epoxide/CO_2_ ring opening copolymerization catalysis is a long-standing challenge. Here, a known highly active Co­(III)­K­(I) catalyst is used to investigate the influences of six different epoxides' binding strengths on their rates of copolymerization. Since calculations and experiments indicate that studying the catalytically relevant Co­(III)–epoxide adduct directly is experimentally challenging, epoxide–catalyst binding interactions are quantified using a Co­(II)­K­(I) complex to model the key catalytic intermediate. Epoxide–catalyst coordination is investigated using UV–vis spectroscopy titrations which provide fast and effective determination of association or binding constants. The epoxide–catalyst equilibrium constants show a clear exponential correlation with copolymerization rates and a new catalyst performance linear free energy relationship is revealed. Epoxides exhibiting stronger catalyst binding constants show higher copolymerization rates. The structure–activity correlation is consistent with the polymerization kinetics, mechanism and DFT calculations. Both the methods to investigate epoxide–catalyst coordination and the linear free energy relationship are shown to apply to the series of six epoxides and a second Co­(III)­K­(I) catalyst. These structure–performance relationships are likely applicable to other transition metal catalysts and should expedite future epoxide and catalyst selection to make useful poly­(carbonate) materials.

## Graphical abstract

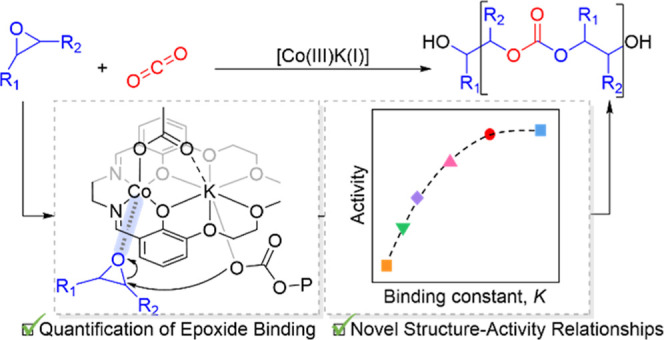

## Introduction

The ring opening copolymerization (ROCOP) of CO_2_ with epoxides is among the most promising strategies to transform CO_2_ waste gas into value added products (Figure 1A).^1–3^ Depending on the structure of the epoxide used, the poly­(carbonates) have a wide range of applications.^3–8^ For example, poly­(propylene carbonate), synthesized from propene oxide (PO)/CO_2_ ROCOP, has its main application as low molecular weight polyols (<10,000 kg mol^–1^) to make polyurethanes or as an electrolyte in batteries.^5,9,10^ In contrast, rigid high molecular weight poly­(carbonates), synthesized from epoxides such as cyclohexene oxide (CHO) or cyclopentene oxide (CPO), are strong engineering plastics and find applications in thermoplastic elastomers.^11,12^ The large property space spanned by CO_2_-based poly­(carbonates) as well as the potential to chemically recycle these materials back to the monomers, makes this an important material class for a future circular plastic economy.^3,6,13–17^

**Figure 1:**
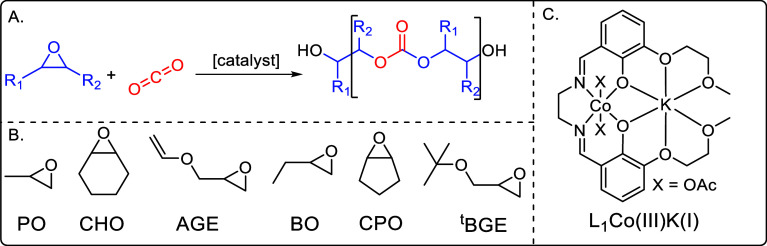
(A) Reaction scheme showing the ring opening copolymerization (ROCOP) of epoxides with CO_2_ forming poly­(carbonates). (B) Illustration of the structures of six commonly used epoxides, where PO: propene oxide, CHO: cyclohexene oxide, AGE: allyl glycidyl ether, BO: butene oxide, CPO: cyclopentene oxide, ^*t*^BGE: *tert* butyl glycidyl ether. (C) Structure of the previously reported catalyst used in this work.^35^

Investigations of new materials rely on being able to synthesize well-defined poly­(carbonates), facilitated by fast, selective and controlled catalysis.^4,7,18,19^ When applying catalysts in the synthesis of (block)­polymers, they need to be active and selective for a wide range of epoxides, including bicyclic epoxides, epoxides with varying steric bulk and side chains containing functional groups (Figure 1B). There are some excellent, highly active and selective epoxide/CO_2_ ROCOP catalysts reported over the past decades. One challenge is that the activity for a particular catalyst depends strongly on the specific epoxide (Figure 2).^4,18,20–33^ For example, using a bicomponent catalyst system, containing a chromium salen complex,(salen)­Cr­(III)­Cl, and a bis­(triphenylphosphine)­iminium azidee (PPNN_3_) cocatalyst, Darensbourg and co-workers observed that when reducing the ring size in cyclic epoxides from a six membered ring (CHO) to a five membered ring (CPO), no activity was observed.^21^ Hence, the reactivity decreased by 100% (TOF = 205 h^–1^ for CHO to poly­(cyclohexene carbonate) (PCHC) vs TOF = 0 h^–1^ for CPO to poly­(cyclopentene carbonate) (PCPC), Figure 2A).^21^ Interestingly, when an organoboron PPNCl catalyst system was used, a rate decrease of an order of magnitude was observed when exchanging CHO for CPO (TOF = 30 h^–1^ for CHO and TOF = 3 h^–1^ for CPO). This is a significantly smaller decrease in activity compared to the chromium salen catalyst (Figure 2C).^23^ Other studies observed that, for example, the addition of one carbon into the side chain of alkyl substituted epoxides (i.e., PO vs butene oxide (BO), Figure 2D) or the addition of one double bond into a ring system led to drastic decreases in the rate of copolymerization (Figure 2).^24^

**Figure 2:**
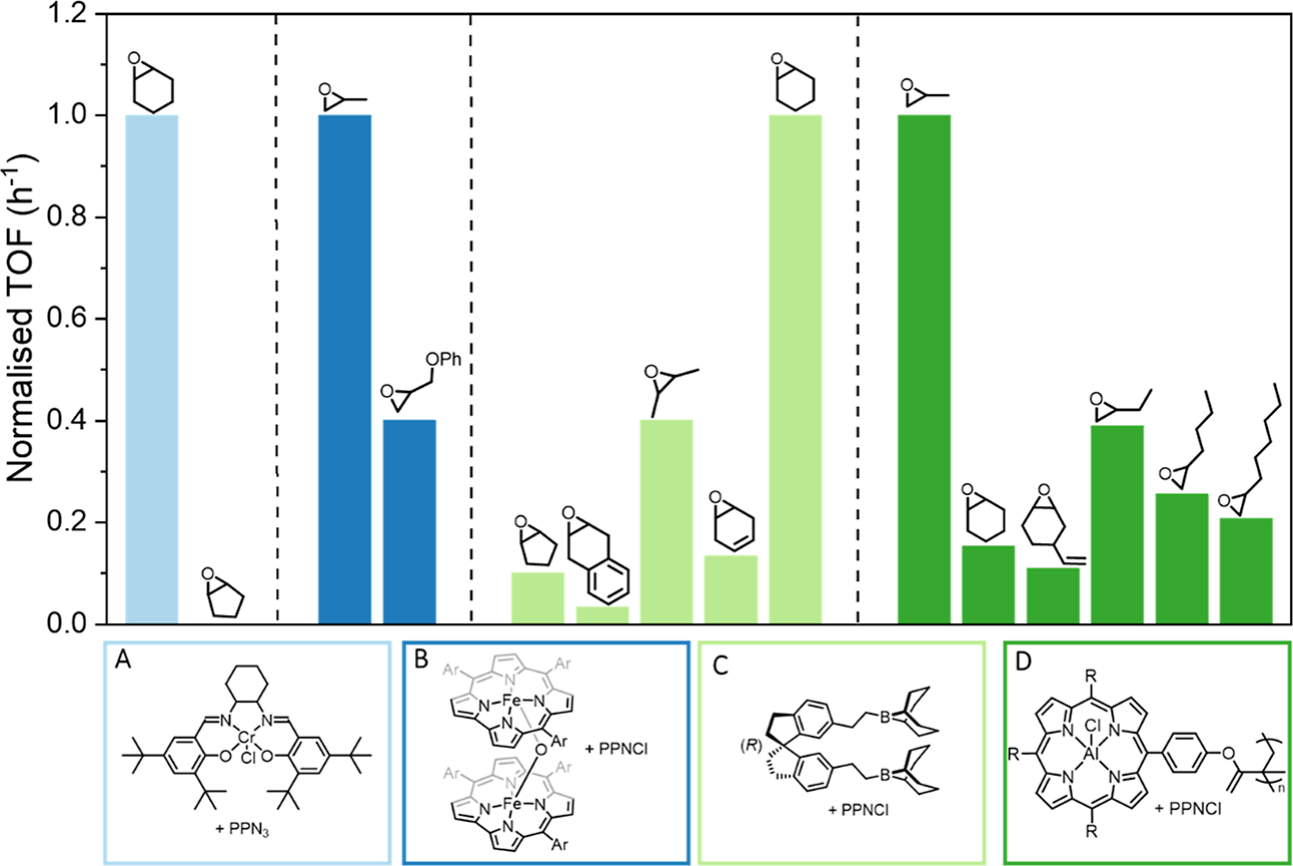
Results from the literature illustrating the difference in catalytic performance for different catalysts and epoxides: bar chart showing the relative turnover frequency (TOF) of four catalyst (A–D) for a range of different epoxides, illustrating the challenges in predicting catalyst performances for different epoxides. TOFs were taken from previous studies and the TOFs for each catalyst were normalized to the highest TOF reported for each catalyst (for full data see Supporting Information Table S1). Reaction conditions: catalyst A: [catalyst A]:[PPN_3_]:[epoxide] = 1:2:500, 35 bar CO_2_, 80 °C, 3 h.^21^ Catalyst B: [catalyst B]:[PPNCl]:[epoxide] = 1:0.5:4000, 20 bar CO_2_, 60 °C, 1 h.^22^ Catalyst C: [catalyst C]:[PPNCl]:[epoxide] = 1:1:500, 20 bar CO_2_, 25 °C, reaction time varied across epoxides.^23^ Catalyst D: [catalyst D]:[PPNCl]:[epoxide] = 1:0.5:10,000, 40 bar CO_2_, 70 °C, 3 h (1 h for PO).^24^

The analysisof previously reported activity data using four excellent catalysts, reveals both unexpected and very significant drops in activity for some epoxide/CO_2_ copolymerizations.^4,18,20–33^ There are substantial and unexplained variations in catalytic activity when using different epoxides; these effects apply generally to most catalysts, including those based on M­(III), M­(II) or organic active sites (Figure 2). Qualitative arguments, such as differences in ring strain or steric bulk, have been invoked to explain the observed variations in epoxide reactivity.^20,24,31,34^

However, these arguments do not allow for a direct comparison between cyclic and acyclic epoxides, and do not explain the drastic variation in activities often observed upon, for example, the addition of one methyl group to a side chain (e.g., Figure 2D, PO vs BO). Further, variations in the epoxide structure do not explain why the same epoxides behaves differently with different catalysts, i.e., the relative rates clearly vary depending on both the catalyst structure and the epoxide structure (Figure 2).

In 2013, Darensbourg proposed that the difference in the rate of copolymerization of PO and styrene oxide (SO) is related to their basicity as quantified using the p*K*_b_ of each epoxide. PO was observed to be a stronger base compared to SO (lower p*K*_b_ of 15.7 vs 16.4) and exhibited a higher rate of copolymerization compared to SO which was shown to be a weaker base and exhibited a slower rate of copolymerization (TOF = 570 h^–1^ vs TOF = 75 h^–1^, respectively).^36^ It was proposed that the basicity of the epoxide correlates to the epoxide binding strength and hence activation of the epoxide. More basic epoxides would be expected to be more strongly bound to the Lewis acidic catalyst. Stronger binding was proposed to lead to a stronger activation of the epoxide, and hence a faster rate of ring opening.^36^

Subsequently, Darensbourg and Yeung employed computational methods to calculate the enthalpies of epoxide binding for different epoxides to M­(III)­(salen)­X/PPNX catalysts, where M = Co­(III) or Cr­(III), X = halide, PPN = bis­(triphenylphosphine)­iminium.^37^ For example, studying four different epoxides (CHO, (*R*)-1,4-cyclohexadiene oxide, (*S*)-1,4-cyclohexadiene oxide and 1,3-cyclohexadiene oxide), suggested that more negative enthalpies of epoxide coordination correlated to increased rates of epoxide/CO_2_ copolymerization.^38^ It was hypothesized that weakly binding epoxides are less able to displace the growing polymer chain prior to the ring opening of the epoxide, resulting in a slower rate of reaction.^38^

Following on from Darensbourg’s computational work, which suggested that the enthalpies of epoxide binding to the catalyst influence copolymerization rates, we hypothesize that the activity of a specific catalyst for a particular epoxide is directly dependent on the strength of the interaction between both the catalyst and the epoxide, rather than the structure of one of them. Here, we aim to investigate this relationship experimentally.

There are a range of methods to study and quantify relationships between substrate structure and reaction rate, perhaps, most famously using Hammett plots.^39^ Hammett plots are an early and widely applied example of a linear free energy relationships (LFERs), relating kinetic and thermodynamic reaction parameters. LFERs have since been employed across many fields to elucidate the relationship between substrate–catalyst interactions and reaction rate.^40–43^ In epoxide/CO_2_ ROCOP catalysis, LFERs have recently been used to study relationships between catalyst structure, rate and selectivity.^44–46^ However, the effect of epoxide-binding on the rate of reaction has not been explored.

To study the interaction between epoxide and catalyst, a recently reported heterodinuclear L_1_Co­(III)­K­(I) catalyst was selected because it shows both high activity and selectivity for the demanding PO/CO_2_ ROCOP over a wide range of polymerization conditions (2–35 bar CO_2_ and 50–80 °C, Figures 1 and 3).^35^ It is anticipated that this catalyst should be active and selective for a wide range of other epoxides, although exactly what controls substrate activity remains to be discovered. A prior thermodynamic and kinetic analysis of PO/CO_2_ ROCOP revealed that the rate-determining step (RDS) is likely the ring opening of the cobalt­(III)-bound epoxide.^35^ Indeed, other Co­(III) catalysts for epoxide/CO_2_ ROCOP are also proposed to show the same RDS (Figure 3, RDS).^32^

**Figure 3:**
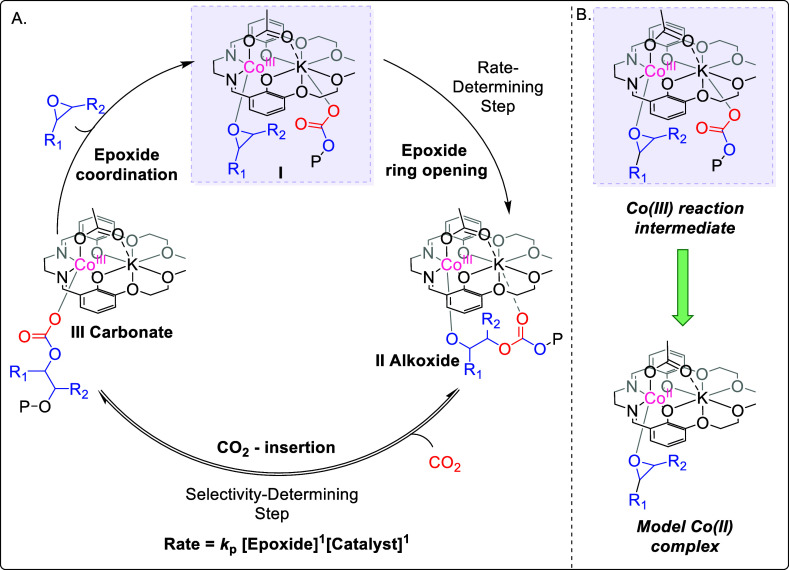
(A) Proposed catalytic cycle for PO/CO_2_ ROCOP using the heterodinuclear L_1_Co­(III)­K­(I) catalyst.^35^ Initiation and chain transfer are not shown in this mechanism, but are detailed in Figure S2. (B) Structure of key reaction intermediate proposed in the rate-determining step of the reaction and the target Co­(II)­K­(I) model complex investigated in this study.

So far, the interaction between epoxides and catalysts has only been explained qualitatively or using computational methods, perhaps because experimental quantifications are challenging.^20,24,31,34,38^ The metal–epoxide adducts, relevant to epoxide/CO_2_ ROCOP, are highly reactive and have proved difficult to isolate (Figure 3).^32^ In other cases, the most stable epoxide–catalyst adduct does not correspond to the true catalytic intermediate. This is a particular challenge in this study since the catalytically relevant Co­(III)–epoxide adduct is not the thermodynamic product of reacting the precatalyst with epoxide. Density functional calculations (DFT) indicate that epoxide coordination at the K­(I) center is slightly more stable than at the Co­(III) center, yet, productive catalysis occurs from the higher energy adduct (vide infra).^19,32,47^ This finding is fully consistent with both experimental and computational studies of related heterodinuclear M­(III)­K­(I) catalysts (M­(III) = Co­(III) and Al­(III)); in all cases the thermodynamic epoxide adduct is coordinated at K­(I).^32,47^ Therefore, the reaction between an epoxide and the L_1_Co­(III)­K­(I) complex is not expected to isolate the catalytically relevant Co­(III)–epoxide adduct. Here, the aim is to investigate catalyst–epoxide binding using a range of other approaches, specifically by applying a L_1_Co­(II)­K­(I) model reaction intermediate and DFT studies (Figure 3B). The L_1_Co­(II)­K­(I) complex is an intermediate in the synthesis of the L_1_Co­(III)­K­(I) catalyst and hence, known to be synthetically accessible. The L_1_Co­(II)­K­(I) complex should have a free Co­(II) coordination site suitable to bind the epoxide, preventing epoxide coordination at K­(I). The objective is to isolate the L_1_Co­(II)­K­(I) complex, and to study its interaction with six different epoxides using UV–visible (UV–vis) spectroscopy titration experiments to quantify the binding constants. Further, the performances of the L_1_Co­(III)­K­(I) catalyst with each of the six epoxides in copolymerizations with carbon dioxide will be evaluated to understand how epoxide binding influences the polymerization rate.

## Results and Discussion

### Catalyst Synthesis and Epoxide Binding Studies

The L_1_Co­(III)­K­(I) catalyst was synthesized using an ancillary ligand (L_1_) which was prepared in high yield from commercial precursors.^35^ The pro-ligand (H_2_L_1_) was reacted with the two metal precursors, Co­(II)­(OAc)_2_ and KOAc, in acetonitrile and under an inert atmosphere, to form L_1_Co­(II)­K­(I)­(OAc) which was isolated as a solid (Figure 4, 99% conversion, 30% isolated yield). The L_1_Co­(II)­K­(I)­(OAc) complex was oxidized in situ by addition of 2 equiv AcOH and stirring in air for 16 h (57% isolated yield).

**Figure 4:**
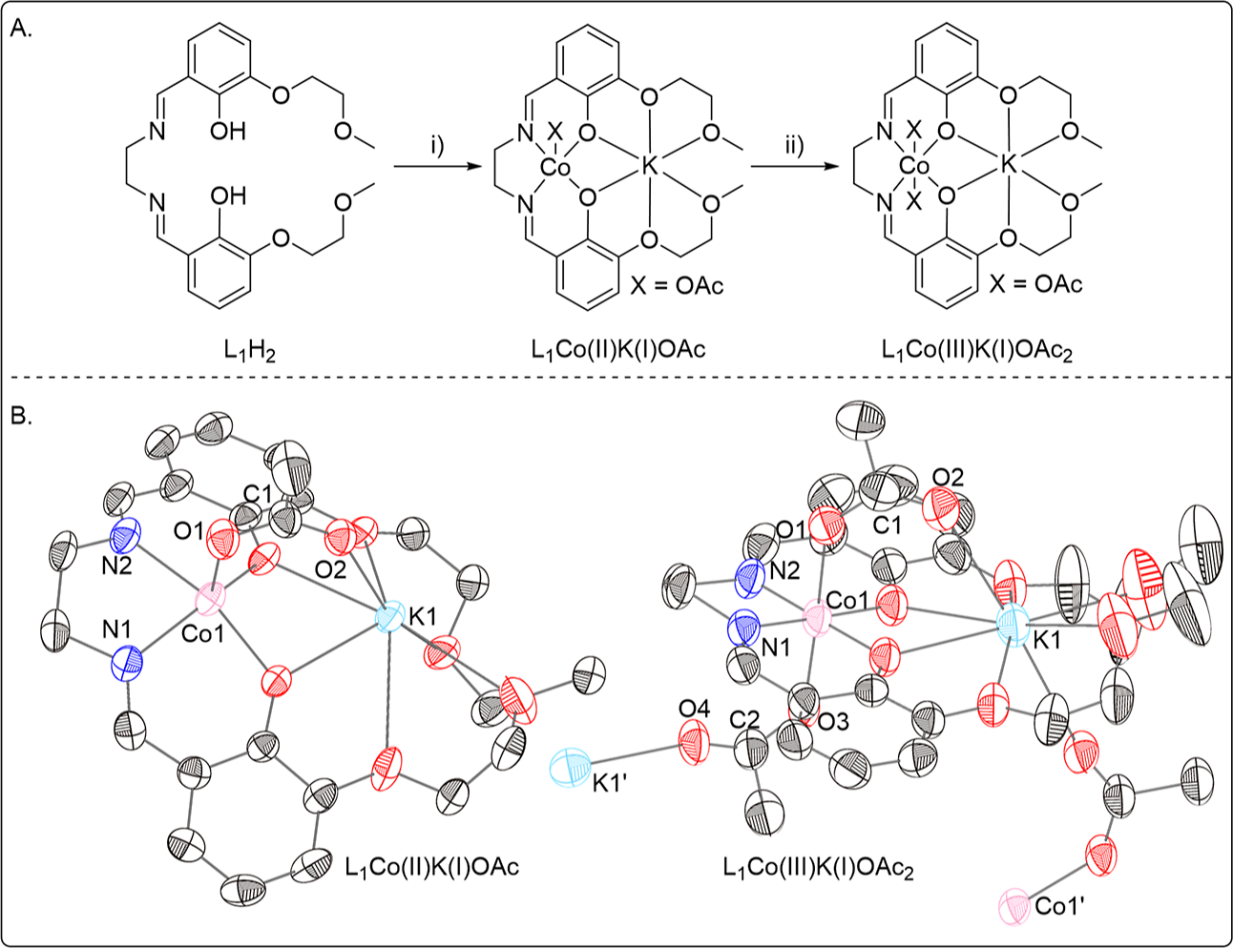
(A) Synthesis of L_1_Co­(II)­K­(I) and L_1_Co­(III)­K­(I), where (i) Co­(II)­(OAc)_2_, KOAc, MeCN (99% conversion, 30% isolated yield (180 mg)) (ii) 2 Equiv. AcOH, MeCN, air (57% isolated yield (870 mg)). (B) Solid state structures of L_1_Co­(II)­K­(I)­(OAc) and L_1_Co­(III)­K­(I)­(OAc)_2_ obtained by single crystal X-ray diffraction. Thermal ellipsoids are shown at a probability of 50%. Hydrogens are omitted for clarity. See Tables S9 and S10 for details.

Successful formation of both L_1_Co­(III)­K­(I) and L_1_Co­(II)­K­(I) was confirmed by single crystal X-ray diffraction (Figure 4, Tables S9 and S10) and UV–vis spectroscopy (Figure S5). The spectra obtained for L_1_Co­(II)­K­(I) and L_1_Co­(III)­K­(I) are in good agreement with previous reports of Co­(II) and Co­(III)­(salen) complexes.^48,49^ The spectrum of L_1_Co­(II)­K­(I) shows three clear transitions at 270 nm, 367 nm and 415 nm. In line with previous reports, these transitions are assigned as a ligand based π → π* transition at 267 nm, a n → π* transition at 367 nm and a d → π* transition at 415 nm (metal-to-ligand charge transfer, Figure S5).^48,49^ For the octahedral L_1_Co­(III)­K­(I), only two clear peaks are present in the UV–vis spectrum. The transition at 292 nm is assigned as a ligand based π → π* transition, while the peak at 397 nm is assigned as a d → π* transition (metal-to-ligand-charge transfer, Figure S5).^50^ The diamagnetic L_1_Co­(III)­K­(I) catalyst was also characterized using multinuclear NMR spectroscopy (Figures S6 and S7) and the novel paramagnetic L_1_Co­(II)­K­(I) complex using IR spectroscopy (Figure S8). Purity of both complexes was confirmed using elemental analysis. The magnetic susceptibility of L_1_Co­(II)­K­(I) was investigated, using Evans NMR spectroscopy method in CD_2_Cl_2_, where the paramagnetic contribution Χ_m_^para^ was estimated as 8.49 × 10^–3^ emu mol^–1^ (Figure S10).^51^ Applying the spin only approximation, the effective magnetic moment, μ_eff_, was estimated as 4.3 μ_B_, which agrees well with the predicted μ_eff_ of 3.9 μ_B_, expected for a square pyramidal, high spin d^7^ complex (see below).^51^

Single crystals of L_1_Co­(II)­K­(I) and L_1_Co­(III)­K­(I) suitable for X-ray diffraction were obtained by slow diffusion of pentane into chloroform solutions of the respective complexes. Structural elucidation confirmed the successful formation of heterodinuclear complexes, in both cases, with the cobalt center coordinated in the phenoxy imine coordination sites and the K­(I) coordinated by the two ether groups. The structure of L_1_Co­(III)­K­(I) catalyst is analogous to that previously reported, with Co­(III) adopting an octahedral coordination geometry.^35^ L_1_Co­(III)­K­(I) is polymeric in the solid state, with two different acetate binding modes: one acetate bridges between the Co­(III) and K­(I) centers bound in the same ligand framework and one bridges a Co­(III) and K­(I) center bound in two different ligand frameworks. In contrast, L_1_Co­(II)­K­(I) is monomeric in the solid state, with only one acetate bridging between the Co­(II) and K­(I) center bound within the same ligand (Figure 4B). As predicted, Co­(II) is five coordinate, adopting a square pyramidal coordination geometry, with an open axial coordination site. The free coordination site is essential since it enables investigation of epoxide binding at the cobalt center.

Before studying the interaction between the epoxide and the model L_1_Co­(II)­K­(I) complex, it is also important to ensure that no catalysis occurs as this would obscure any investigation of the epoxide binding event. Therefore, a series of ^1^H NMR spectroscopy and microkinetic experiments were conducted, the latter using a recently reported kinetic analysis method using differential scanning calorimetry (DSC).^52^ In order to ensure that no reaction occurs between the epoxide and either the L_1_Co­(III)­K­(I) species or the L_1_Co­(II)­K­(I), both complexes were studied (Figure 4, Supporting Information). Using ^1^H NMR spectroscopy, no reaction occurred between 1 equiv. L_1_Co­(III)­K­(I) and either 1 or 2 equiv. of CHO, at room temperature, over 20 h (Figures S11 and S12). In line with this result, no catalysis was observed when combining either the L_1_Co­(III)­K­(I) or L_1_Co­(II)­K­(I) complexes with CHO either in a stoichiometric mixture (1:1) or even when using with a large excess of CHO, as would be used in a binding study (1:4000) over 2 h, at 25 °C (Figures S13–S16). Subsequent analysis of the reaction mixtures by ^1^H NMR spectroscopy confirmed that all the starting equivalents of CHO remained unreacted (vs. mesitylene as an internal standard, Figures S13–S16). These results strongly support prior observations that the L_1_Co­(III)­K­(I) catalysts are not able to form ether linkages and confirm that no catalysis occurs between epoxide and either the Co­(II/III)­K­(I) complexes, at room temperature under the conditions relevant to the epoxide binding study.^31,35^ At higher temperatures (*T* ≥ 50 °C), as generally used in the copolymerizations, initiation (epoxide-binding to the Co­(III)-center and ring opening) is expected to occur.^31,35^

Here, the aim is to study the interaction of epoxides with L_1_Co­(II)­K­(I) complex to form an epoxide bound species that is structurally similar to the key catalytic intermediate (Figure 3B). As discussed, prior experiments and DFT calculations indicate that the L_1_Co­(III)­K­(I)­(OAc)_2_ catalyst cannot be used since epoxide coordination is expected at the K­(I) center, forming a species that is not catalytically relevant.^19,32,47^ Three common epoxides (propene oxide, PO, butene oxide, BO, and cyclohexene oxide, CHO) often used as benchmarks in copolymerization catalysis were selected. Each of these epoxides was reacted with the L_1_Co­(III)­K­(I) catalyst and the L_1_Co­(II)­K­(I) complex, with epoxide coordination investigated using UV–vis spectroscopy titration experiments.

In each experiment, a 0.125 mM solution of L_1_Co­(III)­K­(I) or L_1_Co­(II)­K­(I), in MeCN, was titrated with increasing equivalents of the target epoxide. To allow for any binding events to occur, the solution was agitated after each addition and then left to equilibrate, for 1 min, before the UV–vis spectrum was recorded. Titrations were performed with increasing equivalents of epoxide until saturation was reached. Saturation refers to the observation of no further changes in the spectrum, normalized for catalyst concentration, upon addition of increasing equivalents of epoxide. If no saturation was observed, a maximum of 11,400 equiv. of epoxide were added.

First, the L_1_Co­(III)­K­(I) complex was reacted with increasing equivalents of each of the epoxides (up to 11,400 equiv. of CHO, PO or BO). In each case, there was a hypsochromic shift of the d → π* transition and an increase in intensity (extinction coefficient) at 361 nm (Figures S17–S19). For all three epoxides, a saturation in both, the hypsochromic shift of the d → π* transition as well as the increased absorbance at 361 nm was observed upon addition of around 4000 equiv. of epoxide. However, while saturation of the spectrum is observed at higher wavelength (361 nm), the ligand based π → π* transition at 292 nm continued to linearly increase in intensity with increasing equivalents of epoxide (Figures S17–S19).

No saturation of this transition was observed, even upon the addition of up to 11,400 equiv. (Figures S17–S19, unfilled squares). Previously, similar observations (one peak saturating while another one continues to change in intensity), were attributed to minor quantities of a 1:2 complex at high substrate (epoxide) concentrations.^54^ This lack of saturation of the π → π* transition precludes the estimation of a 1:2 binding constant using easily accessible nonlinear regression modeling.^54^ Therefore, no epoxide binding constant was determined for the interaction between L_1_Co­(III)­K­(I) and BO, PO or CHO (Figures S17–S19). However, it is worth noting, that all three epoxides result in comparable shifts in the UV–vis spectrum of L_1_Co­(III)­K­(I). It is hypothesized that under ambient conditions the epoxide coordination at L_1_Co­(III)­K­(I) occurs primarily at the K­(I) center, while under polymerization conditions coordination is proposed to also occur at the Co­(III) center.^19,32,47^ Since, K­(I) can accommodate high coordination numbers up to 12, the weak coordination of more than one molecule of epoxide is consistent with the continuous changes in the L_1_Co­(III)­K­(I) UV–vis spectrum during epoxide addition.

In contrast, using the L_1_Co­(II)­K­(I) complex and adding around 4000 equiv. of each of the three epoxides (CHO, PO, BO) resulted in significant changes to all the peak intensities (extinction coefficients), and importantly the saturation of all peaks in the UV–vis spectrum. These data are fully consistent with a 1:1 epoxide binding to the L_1_Co­(II)­K­(I) complex (Figures 5, 6, and S21). The titration between the L_1_Co­(II)­K­(I) complex and either propene (PO) or butene oxide (BO) resulted in saturation of all peaks after addition of 3000–4000 equiv. of the epoxide (Figures 5 and S21). From the change in intensity of the d → π* transition at 415 nm, 1:1 association constants were determined as *K*_1_ = 12.1 ± 1.1 M^–1^ for PO and *K*_1_ = 11.7 ± 1.5 M^–1^ for BO. The binding constants were determined using a nonlinear regression model, accessed through the online Bindfit calculator (available at supramolecular.org/bindfit/, Figures 5A,B and S21, Table S2).^53,55^ For CHO, saturation of all peaks was observed at significantly lower equivalents, specifically at 200–250 equiv. (Figure 6A,B). The 1:1 association constant was determined from the change in intensity of the d → π* transition, at 415 nm, over the range of 0–250 equiv. of CHO (Figure 6A,B). The resulting epoxide-complex association constant, *K*_1_(CHO) = 80.99 ± 10.5 M^–1^, is significantly higher than that obtained for either BO or PO.

**Figure 5:**
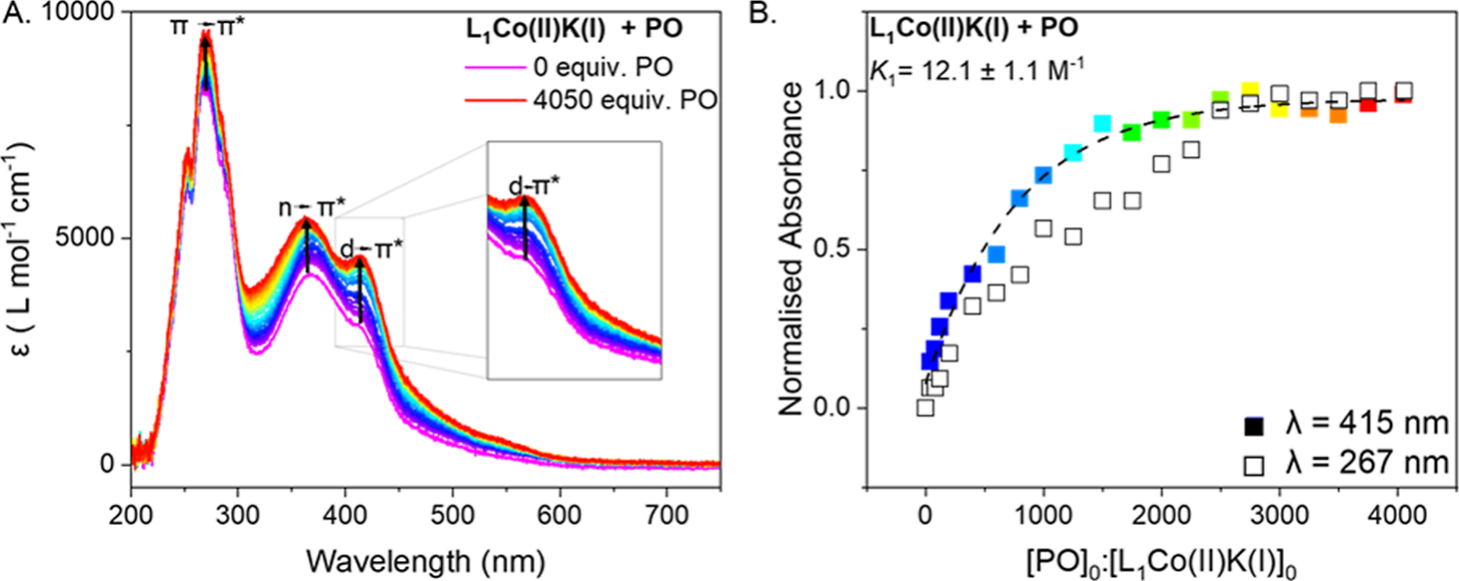
(A) UV–vis spectra obtained by titrating L_1_Co­(II)­K­(I) with increasing equivalents of PO. Increasing equivalents of epoxide are represented by changing colors from purple to blue to yellow to orange and red. Additions were performed in 40 equiv. increments from 0 to 120 equiv. PO, in 200 equiv. increments between 200 and 1000 equiv. PO and in 250 equiv. increments from 1000 to 4050 equiv. PO. (B) Plot showing the change in normalized absorbance of the peak 415 nm (d → π* transition, represented with filled squares) and the peak at 267 nm (π → π* transition, represented with unfilled squares) with increasing equivalents of PO. The association constant *K*_1_ was obtained from the fit of the change in absorbance of the d → π* transition, at 415 nm, between 0 and 4050 equiv. PO using nonlinear regression modeling accessed through the Bindfit calculator.^53^ The fit of the data is accessible through the link provided in Table S2.

**Figure 6:**
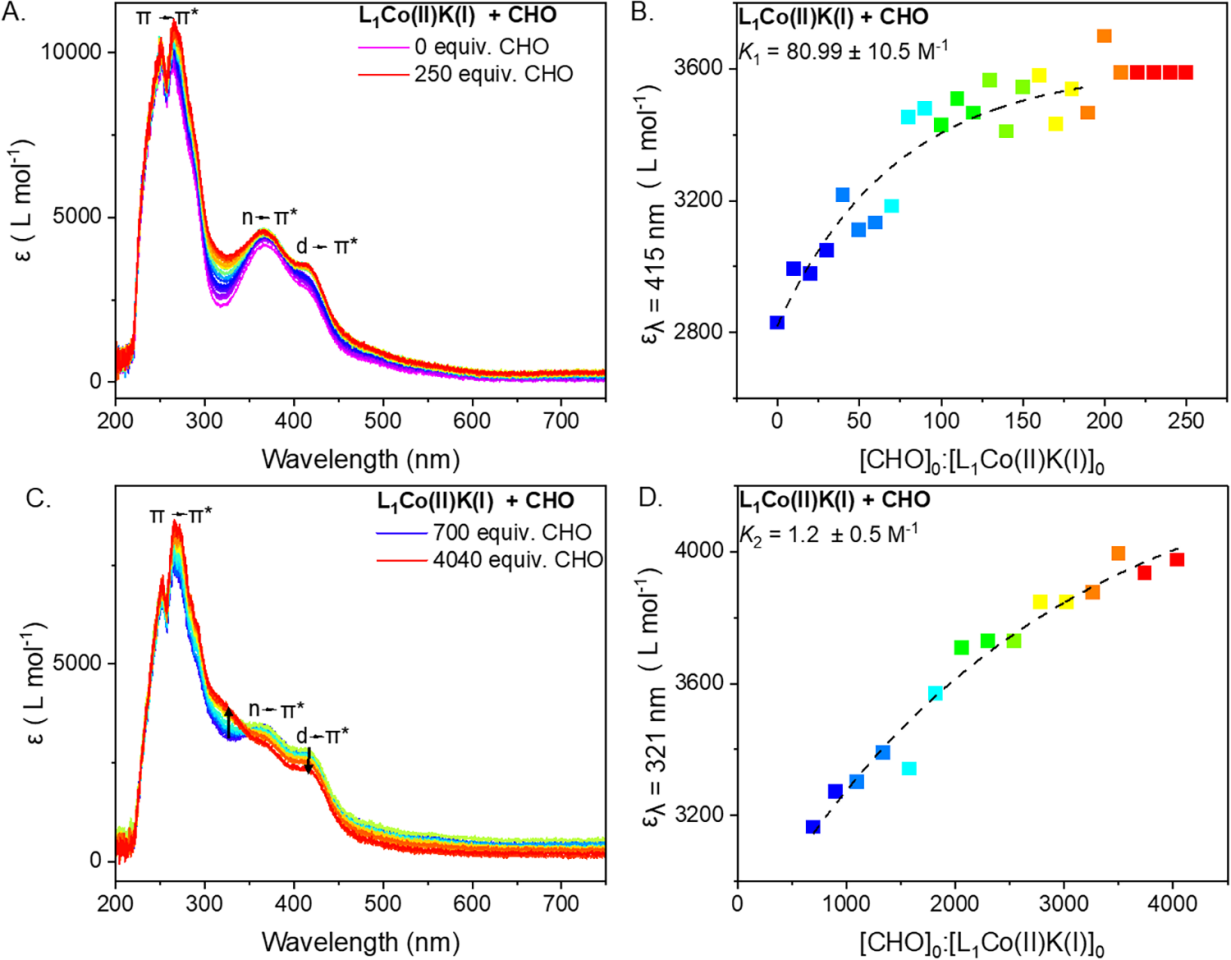
(A) UV–vis spectra obtained by titrating L_1_Co­(II)­K­(I) with increasing equivalents of CHO, demonstrating that saturation occurs at around 200 equiv. of [CHO]_0_/[L_1_Co­(II)­K­(I)]_0_. Increasing equivalents of epoxide are represented by changing colors from purple to blue to yellow to orange and red. Titrations were conducted in 10 equiv. increments. (B) Plot showing the increase in the extinction coefficient at λ = 415 nm with increasing equivalents of CHO. The association constant *K*_1_ was obtained by fitting the increased extinction coefficient between 0 and 250 equiv. of CHO, using supramolecular.org/bindfit/. The fit is accessible through the link provided in Table S2. (C) UV–vis spectra obtained by titrating L_1_Co­(II)­K­(I) with increasing equivalents of CHO, demonstrating that a second binding event occurs between 700–4040 equiv. of CHO. Increasing equivalents of epoxide are represented by changing colors from purple to blue to yellow to orange and red. Titrations were conducted in 200 equiv. increments between 700 and 1100 equiv. of CHO and in 240 equiv. between 1100 and 3740 equiv. of CHO, the final data point was obtained by adding a further 300 equiv. of CHO. (D) Fitting of the UV–vis spectroscopy data shown in (C), specifically at 321 nm, to obtain an association constant *K*_2_, using supramolecular.org/bindfit/. The fitting data is accessible through the link provided in Table S2.

Of the three epoxides, CHO showed the strongest initial association constant, leading to a saturation of the Co­(II) binding site at less than 250 equiv. The experiments using L_1_Co­(III)­K­(I) indicated that when the cobalt center is not available epoxide coordination at the K­(I) center occurs only at higher loadings (of epoxide). To understand whether the Co­(II)­K­(I) complex could also show other epoxide binding events, the addition of more than 250 equiv. of CHO to L_1_Co­(II)­K­(I) was investigated (Figures 6C,D and S20). Upon the addition of 250–500 equiv. of CHO, no change in the UV–vis spectrum was observed (Figure S20). This further supports the interpretation of the first binding event as the formation of a 1:1 adduct between CHO and L_1_Co­(II)­K­(I).

The addition of 700–4040 equiv. of CHO resulted in a decrease in intensity of both the d → π* and n → π* transitions and the formation of a new feature at 321 nm (Figure 6C,D). Given the clear saturation of the first binding event (between 200 and 500 equiv.), this data is interpreted as a second CHO molecule coordinating to the previously formed 1:1 epoxide–L_1_Co­(II)­K­(I) complex (Figure 7B). As the new feature at 321 nm was only observed upon the addition of more than 700 equiv. of CHO, it is proposed to be unique to the formed adduct, and was therefore used to follow this second binding event. Using the change in absorbance at 321 nm and Bindfit, a second epoxide association constant *K*_2_ was determined from the increase of the absorbance at 321 nm over the range of 700–4040 CHO equivalents (Figure 6C,D). As the spectrum only begins to saturated at around 3050 equiv. CHO, *K*_2_ can only be considered an estimate. However, compared with the first CHO binding constant, *K*_1_ = 80.99 ± 10.5 M^–1^, *K*_2_ is significantly smaller, *K*_2_ = 1.2 ± 0.5 M^–1^.

**Figure 7:**
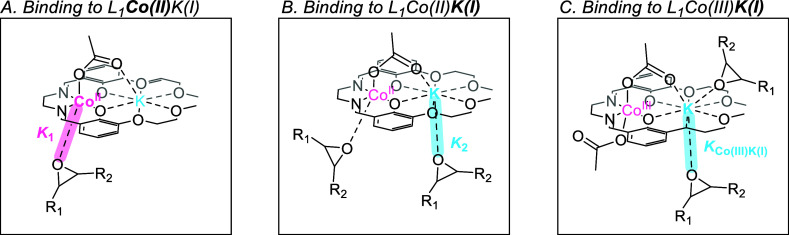
Proposed binding modes and relative interaction strength, as described by *K*_1_, *K*_2_ and *K*_Co(III)K(I)_, of epoxide binding to L_1_Co­(II)­K­(I) and L_1_Co­(III)­K­(I). Initial epoxide coordination is proposed to occur at the Co­(II) center in L_1_Co­(II)­K­(I) (A), followed by coordination of a second epoxide molecule at the K­(I) center in L_1_Co­(II)­K­(I) (B). Binding at the L_1_Co­(III)­K­(I) catalyst is proposed to only occur at the K­(I) center (C).

Based on the experimental data, it is proposed that the initial epoxide coordination occurs at different metals for the L_1_Co­(II)­K­(I) and L_1_Co­(III)­K­(I) complexes (Figure 7A vs C). For all three epoxides, the UV–vis titrations suggested a 1:1 binding event between the L_1_Co­(II)­K­(I) complex and each epoxide. In these experiments, different amounts of CHO, PO and BO were required to reach saturation, consistent with the epoxides having different binding strengths (Figures 5, 6, and S21). In contrast, for L_1_Co­(III)­K­(I) similar changes to the UV–vis spectra were observed for all three epoxides (Figures S17–S19). Based on these observations, it seems unlikely that the epoxides are coordinated to the K­(I) center in both complexes, since there are significant differences in the two complexes’ UV–vis spectra. Consequently, it is proposed that the majority of the epoxide is coordinated at the Co­(II) center in L_1_Co­(II)­K­(I) and at K­(I) in L_1_Co­(III)­K­(I) (Figure 7). Thus, the octahedral Co­(II)–epoxide adduct is an interesting model for the unstable but catalytically relevant Co­(III)–epoxide adduct required for carbon dioxide/epoxide copolymerization (Figures 3 and 7A).

Based on the observed second binding event in the titration of L_1_Co­(II)­K­(I) with CHO, it is proposed that at sufficiently high epoxide concentrations, a second molecule of epoxide coordinates at the K­(I) center of L_1_Co­(II)­K­(I) (Figure 7B). The significant difference in magnitude of *K*_1_ and *K*_2_, is consistent with the epoxide coordination occurring at different metals. If both CHO molecules were coordinated at K­(I), as proposed for L_1_Co­(III)­K­(I), a continuous change in the spectra with increasing epoxide concentrations would be expected. Instead, two distinct binding events are observable in the UV–vis spectra. Furthermore, since binding to Co­(II) should be highly directional (due to the octahedral geometry expected for the transition metals), the strong initial association constant (*K*_1_) is expected. In contrast, epoxide coordination to K­(I) is not geometrically constrained, resulting in a weaker second association constant (*K*_2_, Figures 6 and 7).

Next, the reactions between the L_1_Co­(II)­K­(I) complex and three additional, structurally diverse epoxides was investigated. The epoxides are allyl glycidyl epoxide (AGE), cyclopentene oxide (CPO), and *tert*-butyl glycidyl epoxide (^*t*^BGE) and are selected as the poly­(carbonates) show interesting and distinctive properties. The UV–vis spectroscopy titrations of L_1_Co­(II)­K­(I) with either AGE or CPO yielded similar results to those for PO or BO (Figures S22 and S23). As such, the addition of 0–4000 equiv. of CPO, or 0–1250 equiv. of AGE, caused an initial increase in the intensity (extinction coefficient) of all peaks until saturation occurred. After the absorption was saturated, the addition of further equivalents of CPO did not change the UV–vis spectrum (Figure S22). Adding an even larger excess of AGE, i.e. 2250–4050 equiv., caused a small decrease in the absorbance of the n → π* and d → π* transitions. These results are similar to the putative second binding of CHO to L_1_Co­(II)­K­(I). However, for AGE, the second epoxide coordination resulted in spectral changes that are too small to quantify a second association constant, *K*_2_ (Figure S23).

Upon the addition of ^*t*^BGE to L_1_Co­(II)­K­(I), two distinct binding events were observed (Figure S24), similar to CHO. Following an increase in all peak absorbances between 0 and 240 equiv. of ^*t*^BGE, saturation was observed (Figure S24). Upon the addition of a large excess, i.e. 900–4050 equiv. of ^*t*^BGE, a decrease in the absorbance of the d → π* transition was observed and a new feature formed at 321 nm (Figure S24). This data is interpreted by the initial formation an Co­(II)–epoxide complex, followed by the coordination of the second epoxide molecule at the K­(I) center. Next, the changes in the intensity of the d → π* transitions were used to determine epoxide association constants for AGE, CPO and ^*t*^BGE using Bindfit (Table 1). In line with the saturation at around 200 equiv. of epoxides added, ^*t*^BGE showed the second highest association constant of all epoxides examined with *K*_1_ = 48.6 ± 7.0 M^–1^ (Table 1). The lowest association constant was determined for CPO, with *K*_1_ = 9.3 ± 1.5 M^–1^, and AGE showed an intermediate association constant of *K*_1_ = 29.0 ± 6.9 M^–1^. For ^*t*^BGE, an additional, second association constant was obtained from the change in absorbance associated with the formation of a new feature at 321 nm upon the addition of 900–4050 equiv. of ^*t*^BGE. In line with the results for the binding of CHO, this second association constant for ^*t*^BGE was also significantly smaller than the first one, *K*_2_ = 2.09 ± 0.1 M^–1^, further supporting the proposed binding modes at the L_1_Co­(II)­K­(I) complex (Figure 7).

**Table 1:**
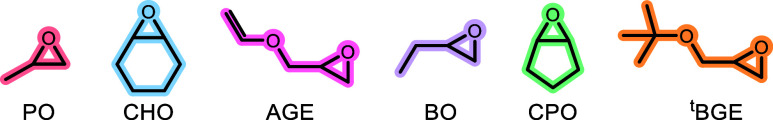
Overview of the Binding Data and Polymerization Data, Obtained from UV–Vis Spectroscopy Studies, for Six Epoxides

Epoxide	*K*_1_/M^–1^ ^a^	*q*_UV/vis_/%^b^	*K*_*q*=1_/M^–1^ ^c^	TOF/h^–1^ ^d^	*k*_p_/×10^–3^ M^–1^ s^–1^ ^e^
CHO	80.99 ± 10.5	71	114.1 ± 14.7	328 ± 8	16.7 ± 0.26
PO^f^	12.1 ± 1.1	85	14.2 ± 1.3	445 ± 24 ^35^	11.2 ± 0.002 ^35^
AGE	29.0 ± 6.9	81	35.6 ± 8.5	234 ± 5	8.21 ± 0.2
BO	11.7 ± 1.5	85	13.8 ± 1.8	255 ± 5	7.99 ± 0.0005
CPO	9.3 ± 1.5	81	11.4 ± 1.9	153 ± 2	4.59 ± 0.05
^*t*^BGE	48.6 ± 7.0	59	83.0 ± 12.1	229 ± 14	9.79 ± 0.35

a *K*_1_ as calculated using supramolecular.org/bindfit/, using a 1:1 binding model, from the titration experiments of L_1_Co­(II)­K­(I) complex with increasing equivalents of epoxide, up to the first saturation point. All fits can be accessed via the links available in Table S2.

b *q* was calculated from *K*_1_ as detailed in eqs S1–S4.

c *K*_*q*=1_ was determined by dividing *K*_1_/*q*. Polymerizations were conducted using L_1_Co­(III)­K­(I) as the catalyst; polymerization conditions: [1]/[*trans*-1,2-cyclohexene diol]/[epoxide] = 1:20:4000, 6 mL neat epoxide, 20 bar CO_2_, 50 °C.

d TOF was determined by dividing turnover number (TON) by the reaction time, where TON was determined by dividing the moles of epoxide consumed, as determined from the ^1^H NMR spectrum of the polymer vs mesitylene as an internal standard (10 equiv mesitylene/4000 equiv of epoxide); all reactions show >99% CO_2_ selectivity and >90% poly­(carbonate) selectivity, for details see Table S3.

e *k*_p_ was determined by dividing *k*_obs_ by the concentration of catalyst used, where *k*_obs_ was determined from the plot of ln­([epoxide])/ln­([epoxide]_0_) against time.

f Entry taken from ref 35.

Furthermore, from the association constants (*K*_1_) and the known initial epoxide ([epoxide]_0_) and catalyst concentrations ([L_1_Co­(II)­K­(I)]_0_), the percentage of the 1:1 catalyst–epoxide adduct (denoted as [L_1_Co­(II)­K­(I):epoxide]) vs free catalyst, from here onward denoted as *q*, was obtained (eqs S1–S4). Across the series of epoxides, *q* was generally high at 60–80% (Table 1). All binding experiments were conducted under highly dilute conditions in MeCN; however, the copolymerizations are all conducted in neat epoxide. It is, therefore, expected that under copolymerization conditions most of the catalyst will exist in the epoxide-bound form. The association constants obtained by UV–vis spectroscopy are, therefore, normalized to the extent of binding in all subsequent discussions and analyses (Table 1, *K*_*q*=1_).

Following the successful application of UV–vis spectroscopy to quantify epoxide binding to the isolated L_1_Co­(II)­K­(I), it was hypothesized that the L_1_Co­(II)­K­(I) complex could also be obtained in situ from L_1_Co­(III)­K­(I) using cyclic voltammetry (CV). Inspired by a recent report by Yang and co-workers, in which the binding of different solvents to a vanadyl complex was studied using CV, we wondered whether epoxide binding to the L_1_Co­(II)­K­(I) complex could be studied using electrochemistry.^56^ To investigate the proposed epoxide binding, the electrochemistry of the L_1_Co­(III)­K­(I) catalyst was first investigated in the absence of any epoxide. All electrochemical experiments were performed with 1 mM solutions of the L_1_Co­(III)­K­(I) catalyst, in MeCN, using 0.1 M tetrabutylammonium hexafluorophosphate (TBAPF_6_) as the electrolyte, in a N_2_-filled glovebox. All cyclic voltammograms were individually referenced to the ferrocenium/ferrocene couple (denoted as Fc^+^/Fc^0^). The cyclic voltammogram of L_1_Co­(III)­K­(I) exhibits a reversible feature at *E*_1/2_ = −1.66 V (vs Fc^+^/Fc^0^), which, in line with previous reports on Co­(II)­(salen) complexes, is assigned to a Co­(II/I) redox couple (Figure S31B).^49^ In addition to this clearly reversible redox event, a second reduction peak at *E*_red_ = −1.52 V (vs Fc^+^/Fc^0^) and an oxidation at *E*_ox_ = −0.35 V (vs Fc^+^/Fc^0^) were observed. Further investigations failed to clearly assign these irreversible redox events, including experiments to uncover their scan rate dependence and comparison to a mono-Co­(III) complex (see Supporting Information for further details). Indeed, prior reports of related Co­(III)­(salen) complexes also show similar peaks but without clear redox process assignments.^49,57,58^

To establish whether, in the future, electrochemistry could help estimate the extent of epoxide binding to cobalt catalysts, preliminary binding experiments were conducted, using L_1_Co­(III)­K­(I). A 1 mM solution of L_1_Co­(III)­K­(I) was titrated with increasing concentrations of the target epoxide and the CV was measured after each addition and the position of the reduction peak at −1.52 V (vs Fc^+^/Fc^0^) was monitored. Each experiment was stopped once the number of equivalents of epoxide that led to saturation in the UV–vis spectroscopy experiment were reached, i.e., after addition of 4000 equiv. of PO and BO, 2000 equiv. of AGE and 200 equiv. of CHO. Upon the addition of increasing concentrations of each epoxide a significant shift in the position of this reduction peak was observed (Figure S33). Plotting the shift in the reduction potential (at the maximum epoxide concentration) to the epoxide binding constants, determined by UV–vis spectroscopy titrations, showed a linear correlation (*R* ^2^ = 0.932 for the linear fit, Figure S34). This apparent correlation highlights that future electrochemical binding measurements should be investigated where transition metal redox transitions are reversible and easily assigned.

### DFT Calculations on the Co­(III)­K­(I) Catalyst

In order to further contextualize the experimental binding studies, we conducted a DFT investigation of the catalytic cycle for the L_1_Co­(III)­K­(I) catalyst in PO/CO_2_ ROCOP (Figure 8, Supporting Information for details). Initiation is the first step and involves an acetate coligand, bound to the L_1_Co­(III)­K­(I) catalyst, ring opening the first molecule of epoxide to form an alkoxide intermediate. In the initiation step, the L_1_Co­(III)­K­(I) catalyst must coordinate the first molecule of PO either at the Co­(III) or the K­(I) center. The calculated free enthalpy of formation of the Co­(III)-bound PO adduct is significantly higher at +6.8 kcal mol^–1^ (i.e., endergonic) than the formation of the K­(I)-bound PO adduct at −1.4 kcal mol^–1^ (i.e., exergonic).

**Figure 8:**
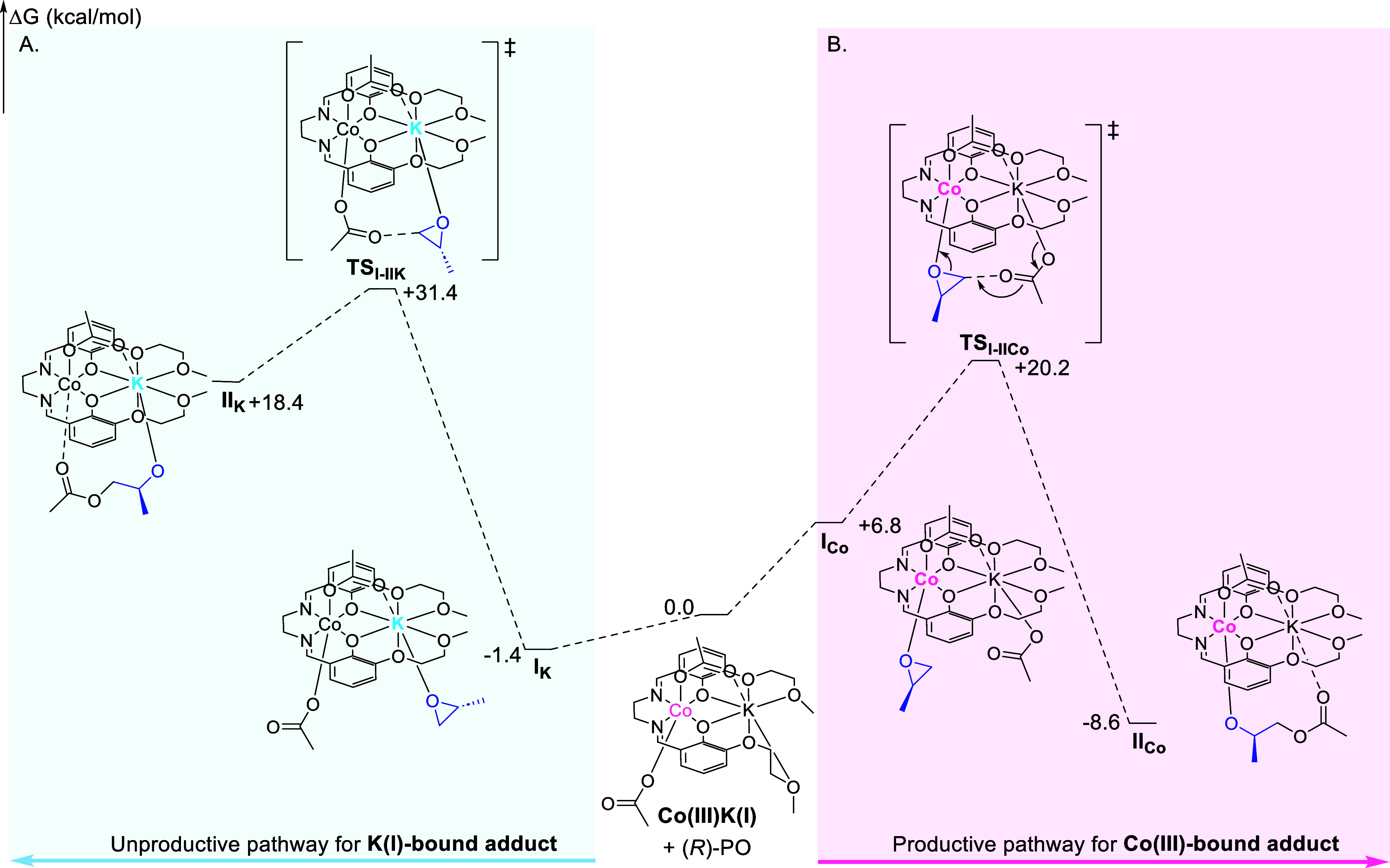
Illustration of the DFT calculated potential energy surface for the initiation and first transition state occurring during the copolymerization of PO with CO_2_ using the L_1_Co­(III)­K­(I) catalyst, where (A) PO coordination occurs at the K­(I) center, and (B) PO coordination occurs at the Co­(III) center. The split valence 6-31 + g­(d,p) basis sets were used for carbon and hydrogen. This lower basis set was chosen as these elements do not bind directly to either catalytic metal center, but extra diffuse functions were added to capture more mid- and long-range interactions, for instance with growing polymer chains. The triple-ζ 6-311 + g­(d) basis set was used for potassium and all heteroatoms. Cobalt centers were described with the Stuttgart SDD ECP and associated basis sets.

This difference in the free enthalpy barrier for epoxide binding is fully consistent with the experimental observation that weak binding occurs between PO and the L_1_Co­(III)­K­(I) catalyst. It is proposed that at room temperature the Co­(III)–epoxide adduct is not formed, and instead only the weak binding of PO to K­(I) is experimentally observed. Furthermore, DFT modeling of the following transition states to alkoxide formation (TS_I–II_) demonstrates that the barrier from the thermodynamic K­(I)–epoxide adduct is significantly higher than that from the Co­(III)–epoxide adduct (ΔΔ*G* = 31.4 kcal mol^–1^ vs ΔΔ*G* = 20.2 kcal mol^–1^). Similar free enthalpy profiles are calculated for a model propagation step, confirming that the productive and unproductive pathways remain the same throughout the polymerization (see Supporting Information).

The Co­(III)–epoxide adduct is, therefore, the key catalytic intermediate for a productive copolymerization pathway. This finding underlines the need to experimentally study cobalt–epoxide adducts, as the K­(I)–epoxide adduct is not a catalytically relevant intermediate. The calculated differences in free enthalpy to access the different metal adducts demonstrates that isolating the Co­(III)–epoxide adduct would be not feasible. The computational study further underlines the benefits of using the Co­(II)­K­(I) complex to model the cobalt–epoxide adduct.

### Epoxide Structure–Catalyst Activity Correlations

Considering both the experimental and computational results, it is proposed that epoxide binding to the L_1_Co­(II)­K­(I) model complex, informs upon the catalytically relevant Co­(III)–epoxide adduct. Comparison of the experimental association constants obtained for epoxide binding to L_1_Co­(II)­K­(I) across the six different epoxides reveals very different binding strengths. Based on the difference in epoxide binding strength, and previous reports on the effect of epoxide structure on the rate of polymerization, a wide range of polymerization rates might be expected across the series.^20,38^

To investigate the effect of the epoxide binding strength on the rate of copolymerization, ring opening copolymerizations were conducted using the L_1_Co­(III)­K­(I) catalyst and each of the six epoxides. Demanding conditions of [catalyst]/[1,2-*trans* cyclohexene diol]/[epoxide] = 1:20:4000 in neat epoxide, 20 bar CO_2_ pressure and 50 °C were applied (Tables 1 and S3). All reactions were performed in an autoclave fitted with an in situ IR spectroscopy probe, allowing for the monitoring of the formation of both poly­(carbonate) (1750 cm^–1^) and any cyclic carbonate byproduct (1810 cm^–1^). Epoxide conversion was determined at the reaction completion from an aliquot which was analyzed using ^1^H NMR spectroscopy (using mesitylene as an internal standard). The propagation rate coefficients were obtained by dividing the observed pseudo first order rate coefficient (*k*_obs_) by catalyst concentration, where *k*_obs_ is the gradient of the linear fit of ln­([epoxide])­([epoxide]_0_) vs time data (exemplified in Figure S25). The *k*_p_ values revealed that CHO polymerized with the highest rate of *k*_p_ = 16.7 × 10^–3^ ± 0.26 s^–1^ M^–1^. All other epoxides exhibited intermediate rates, ranging between *k*_p_ = 5–11 × 10^–3^ s^–1^ M^–1^. For all copolymerizations quantitative CO_2_ uptake (>99%) and high selectivity for poly­(carbonate) vs the cyclic carbonate was observed (>90%, Table S3).

Plotting the propagation rate constant *k*_p_, normalized to the concentration of each epoxide vs the observed epoxide–Co­(II)­K­(I) association constant, *K*_*q*=1_, for all six epoxides, reveals an exponential relationship (Figure 9A). Plotting the log­(*k*_p_) vs log­(*K*) shows a linear relationship (Figure 9B). These results indicate that there is a true LFER between the rate of copolymerization and the epoxide–catalyst association constant. This LFER highlights that the variation in the rates of polymerization between different epoxides is affected by the binding strength of those epoxides to the catalyst. It has previously been proposed that epoxide binding to Lewis acidic metals, might affect its polarization, specifically that it may weaken the O–C bond.^44,45,59^ In the catalytic rate determining step ring opening of a cobalt bound epoxide (by attack of a carbonate nucleophile) occurs.^32,35^ Stronger epoxide binding, e.g. as observed for CHO should lead to greater bond polarization and, hence, faster rates of copolymerization. In contrast, weak binding, e.g. as observed for CPO, is expected to result in much weaker C–O bond polarization which correlates with the lower rates of copolymerization (Figure 9C).

**Figure 9:**
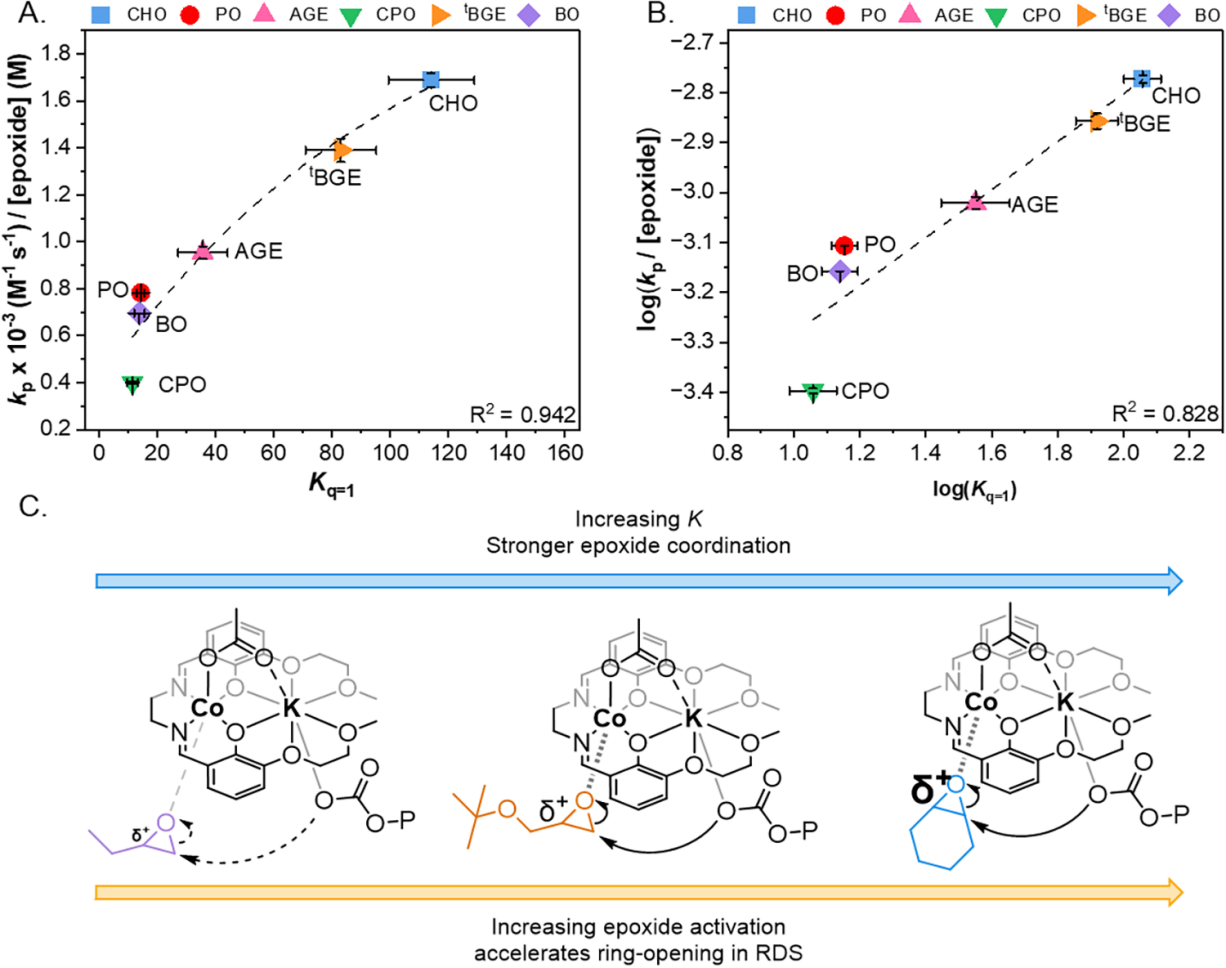
(A) Exponential plot of *k*_p_ normalized to the neat concentration of each epoxide vs *K*_*q*=1_ for epoxide/CO_2_ ROCOP catalyzed by L_1_Co­(III)­K­(I), where *K* was obtained from UV–vis spectroscopy binding experiments. Error bars on polymerization data represent the standard error of the mean obtained from *n* = 2, error bars on the binding constant were obtained from the nonlinear fitting of the UV–vis spectroscopy data (Table S2). (B) Linear plot of log­(*k*_p_/[epoxide]) vs log­(*K*), showing that the observed correlation is a true LFER. (C) Structure of the proposed key reaction intermediate and the effect of epoxide binding on the rate determining step (epoxide ring opening).

DFT calculations on ring opening barriers (TS_I–IICo_, Figures 8 and S28) for different epoxides using the Co­(III)­K­(I) catalyst, reveal activation barriers in the range of 20 - 22.5 kcal mol^–1^ for the different epoxides. Given the relatively small differences between the calculated activation barriers, the computed barriers are expected to be error prone, especially with respect to the effect of conformational entropy. Despite these limitations, plotting the experimentally determined binding constants, *K*_*q*=1_ and polymerization rates against the calculated barriers to epoxide ring opening reveals two weak linear correlations (Figures S29 and S30). These correlations further support the proposed mechanistic interpretation of the experimentally discovered LFER, as the proposed difference in epoxide binding strength and hence, epoxide polarization is expected to affect the barrier to epoxide ring opening, and hence rate of reaction, as observed by DFT. This agreement between calculated and experimental measures, further underlines that the L_1_Co­(II)­K­(I) model complex can be used to determine epoxide binding strengths and these values inform upon thepolymerization mechanism using the L_1_Co­(III)­K­(I) catalyst.

Previously, differences in epoxide structures such as steric hindrance (alkyl substituted epoxides) or ring strain (cyclic epoxides) were proposed to rationalize different copolymerization activity data (using the same catalyst).^24,31,34^ Only Darensbourg and co-workers had hypothesized that epoxide basicity or binding strength could affect rates of copolymerization rates.^36–38^ This work reveals the first evidence for a direct correlation between epoxide–catalyst binding strength and polymerization propagation rate coefficient. It demonstrates that rather than a single driving force, such as ring strain, the reactivity of an epoxide may be directly dependent on its catalytic active site binding strength. Epoxide binding strength is likely dependent on a combination of factors, including contributions from ring strain and substituents. Importantly, the strength of the binding interaction between an epoxide and the catalyst is dependent on both the epoxide and the electronic and steric properties of the catalytic metal center where coordination occurs. Considering epoxide binding strength as one of the key factors determining epoxide reactivity, explains why the same epoxides show different absolute and relative rates with different catalysts (Figures 1 and 2).

The UV–vis spectroscopy methodology presented in this work allows investigation of a model complex of a key intermediate in the catalytic cycle and direct quantification of the epoxide binding strength. The method may be applicable to other catalysts containing redox active metal centers. Given the high number of Co­(III) and Cr­(III) catalysts in the literature, it is envisaged that this method could be generally used to quantify the effect of epoxide structure on the polymerization rate for other widely used catalysts.^4,18^

To test this hypothesis of more general applicability, we conducted a further, preliminary study using a previously reported dinuclear L_2_Co­(III)­K­(I) catalyst, known to be active in epoxide/CO_2_ ROCOP for a range of structurally diverse epoxides.^31^ Both the previously reported L_2_Co­(III)­K­(I) and the corresponding L_2_Co­(II)­K­(I) complex were isolated and characterized using NMR spectroscopy, IR spectroscopy and elemental analysis (Supporting Information, Figures S35–S42). Single XRD data obtained for L_2_Co­(II)­K­(I) confirmed a free coordination site at the Co­(II) center (Figure S39). Next, the binding of six epoxides (CHO, PO, BO, AGE, CPO and ^*t*^BGE) to L_2_Co­(II)­K­(I) was studied using the UV–vis spectroscopy titration method (Figures S43 and S44, Table S7). In line with the results obtained for L_1_Co­(II)­K­(I), the strongest binding was observed for CHO while the lowest binding constant was obtained for CPO (Figures S43 and S44, Table S7). Next, the performance of L_2_Co­(III)­K­(I) was studied for the copolymerization of BO, AGE, CPO and ^*t*^BGE with CO_2_ and compared to previously reported data for the ROCOP of PO/CO_2_ and CHO/CO_2_ under the same reaction conditions (1:20:4000, [cat]/[1,2-*trans*-cyclohexane diol]/[epoxide], neat epoxide, 20 bar CO_2_, 50 °C, Table S8). While CPO, BO, PO and CHO lead to the formation of over 99% poly­(carbonate), when using AGE and ^*t*^BGE around 20% of cyclic carbonate formation was observed. When plotting the polymerization rate constant, normalized by epoxide concentration, over the binding constants, determined by UV–vis spectroscopy, a correlation between the two parameters becomes apparent (Figure S45). However, it is important to note that AGE and ^*t*^BGE show rates lower than expected, from the LFER. This is attributed to the lower poly­(carbonate) selectivity using these two epoxides.

The preliminary study of epoxide binding to the second cobalt catalyst illustrates that the generality of the LFER between polymerization activity and epoxide binding constant. The data for L_2_Co­(III)­K­(I) indicates that these correlations may be very sensitive to catalyst selectivity and may not be applicable to systems that have a low selectivity for the polymer product. Testing the methodology more broadly is a future priority.

If generally applicable, this method may also be useful to investigate the effect of varying ligand or metal centers on the binding strength of a particular epoxide, thereby helping to select the best “epoxide–catalyst” combination for the synthesis of a desired poly­(carbonate). In contrast to polymerization experiments, the measurements of epoxide binding strength can be performed with very small quantities of catalyst and epoxide. Quantification of epoxide binding strength prior to catalysis experiments could help to improve the polymerization, as understanding the binding strength should indicate the optimum conditions under which to conduct the copolymerization. For example, if a low binding strength is observed in the spectroscopic experiments, the polymerization reaction could be run at higher catalyst loadings to increase rates of reaction. Furthermore, binding experiments could help to narrow epoxide choice when considering the design of new materials: if two epoxides are expected to give similar, desired material properties, the new method could be used to determine which epoxide will exhibit the faster rate of polymerization. For example, often either CHO or CPO are used to introduce rigidity into block polymer structures.^60^ When considering which of the two epoxides to choose, the new method reveals very different binding to the catalyst (*K*_CPO_ = 11.4 ± 1.9 M^–1^ vs *K*_CHO_ = 114.1 ± 14.7 M^–1^) and indicates that CHO will exhibit a significantly faster rate of polymerization than CPO (*k*_p_ = 16.7 ± 0.26 × 10^–3^ M^–1^ s^–1^ for CHO vs *k*_p_ = 4.59 ± 0.05 × 10^–3^ M^–1^ s^–1^ for CPO). Thus, the selection of CHO would be preferable for this L_1_Co­(III)­K­(I) catalyst. While similar predictions of differences in polymerization rates could be made using DFT calculations for more extreme rate differences, as observed for CHO and CPO (Table 1, Figures S29 and S30), smaller differences in rate, for example, between AGE and BO, are less clearly predictable using DFT calculations. This highlights that the experimental method allows for more accurate predictions than computational methods.

Finally, it should be noted that related chiral Co­(III) catalysts show outstanding activity and iso-selectivity in epoxide ring opening polymerization to produce isotactic polyethers.^61^ Large differences in rate between PO and BO enchainment were also observed (TOF_PO_ = 5440 h^–1^ vs TOF_BO_ = 880 h^–1^) and it may be worthwhile to understand whether related catalyst–epoxide binding chemistry accounts for the differences in rates. Another question that warrants further attention is whether differences in epoxide binding strength also translate to differences in the binding strength of other intermediates, i.e., whether an alkoxide formed by ring opening of CHO binds more strongly than an alkoxide formed by PO ring opening. Similar UV–vis spectroscopy titrations could be used to investigate this by monitoring the binding of different alkoxides to the Co­(II)­K­(I) complex.

## Conclusion

A known highly active L_1_Co­(III)­K­(I) catalyst was used to investigate the effect of epoxide binding strength on the rate of epoxide/CO_2_ copolymerization. Epoxide binding was studied using a L_1_Co­(II)­K­(I) model compound which is a mimic of the key Co­(III) reaction intermediate. The binding of six epoxides to the model compound was studied using UV–vis spectroscopy, allowing for the quantification of the epoxide–catalyst interaction strength through an association constant (*K*). The new method is proposed to be widely applicable to study the interaction of substrates with other M­(III) catalysts, thereby furthering the understanding of the interplay between catalyst and epoxide structure.

Following the quantification of binding strength, all six epoxides were tested in the ring opening copolymerization using a previously reported L_1_Co­(III)­K­(I) catalyst and a linear free energy relationship between the propagation rate coefficient and the association constant for all six epoxides was observed. For the first time, this study establishes an experimentally quantified relationship between epoxide binding strength and copolymerization rate. This result indicates that epoxide binding strength is a key factor in determining the reactivity of a particular epoxide with a specific catalyst. This hypothesis is further supported by an additional (weaker) correlation between calculated barriers to epoxide ring opening and experimentally determined association constants, which indicate that epoxide binding also affects the transition state of the rate determining step. This study provides the first quantitative insights into the relationship between epoxide structure and polymerization rate. It is hypothesized that epoxide binding strength is dependent on both epoxide and catalyst structure. This highlights that, in the future, catalysts should be specifically targeted for one epoxide, as the steric and electronic requirements for the catalyst may vary drastically depending on the epoxide chosen.

## Supplementary Material



## Notes

The spectroscopic data that supports the characterization of all complexes is available at Oxford University Research Archive, ORA, DOI: https://dx.doi.org/10.5287/ora-w4qq2ezmo. The Supporting Information is available free of charge at https://pubs.acs.org/doi/10.1021/jacs.5c09088.

Characterization data of catalysts (1D and 2D NMR spectra, infrared spectra, cyclic voltammograms, and X-ray crystallography); DFT calculations (PDF)

## ∥

David H Koch Institute for Integrative Cancer Research, Massachusetts Institute of Technology, Cambridge, MA 02139, USA

## Abbreviations

CV: cyclic voltammetry
ROCOP: ring opening copolymerization
PO: propene oxide
CHO: cyclohexene oxide
AGE: allyl glycidyl ether
BO: butene oxide
CPO: cyclopentene oxide
^*t*^BGE: *tert* butyl glycidyl ether
UV–vis: UV–visible
RDS: rate determining step
