# Supplementary material for: Quantifying the Influences of Epoxide Binding in Epoxide/CO2 Ring Opening Copolymerization Catalysis
Source: J Am Chem Soc. 2026 Feb 11;148(7):6826–39. doi: 10.1021/jacs.5c09088 (PMC12951444; doi:10.1021/jacs.5c09088)
Supplement: Supplementary file 1 [file ja5c09088_si_001.pdf]

Supporting Information

Quantifying the Influences of Epoxide Binding in Epoxide/CO_2 Ring Opening Copolymerization Catalysis

Katharina H.S. Eisenhardt,[a] Francesca Fiorentini,[a] Jae Elise L. Payong,[b] Ute L. Petri,[b] Antoine Buchard,[c] Jenny Yang,[b]* Charlotte K. Williams[a]*

[a]Department of Chemistry, Chemistry Research Laboratory, University of Oxford, Oxford OX1 3TA, UK

[b]Department of Chemistry, University of California, Irvine, Irvine, California 92697, USA

[c]Department of Chemistry, Green Chemistry Centre of Excellence, University of York YO10 5DD, UK

Table of Contents

Materials and Methods 6

NMR spectroscopy 6

FT-IR measurements 6

Gel permeation chromatography (GPC): 6

CV studies 6

UV-Vis spectroscopy studies 6

Differential scanning calorimetry (DSC) 6

Elemental Analysis 7

General Epoxide/CO_2 ROCOP Procedure 8

Synthesis of the ligand (H_2L_1)[1] 8

L_1Co(II)K(I) synthesis 8

L_1Co(III)K(I) synthesis[1] 8

L_2Co(II)K(I) synthesis 9

L_2Co(III)K(I) synthesis[3] 9

Additional Information 10

Table S1 Data for the bar chart shown in Fig. 2. 10

Fig. S2 General mechanism of epoxide/CO_2 ROCOP for a metal acetate catalyst, including initiation and chain transfer 11

Fig. S3 ^1H NMR spectrum of the previously reported ligand H_2L_1 reported in CD_2Cl_2. 12

Fig. S4 $^{13}C\{^1H\}$ NMR spectrum of the previously reported ligand H_2L_1 recorded in CD_2Cl_2. 12

Fig. S5 UV-Vis spectra of A. Co(II)K(I) and B. Co(III)K(I). Spectra were collected at 0.125 mM catalyst, in MeCN, under an inert N_2 atmosphere in a glovebox. 13

Fig. S6 ^1H NMR spectrum of Co(III)K(I) measured in CD_2Cl_2 14

Fig. S7 $^{13}C\{^1H\}$ NMR of Co(III)K(I) measured in CD_2Cl_2 14

Fig. S8 IR spectrum of the Co(II)K(I) complex. 15

Fig. S9 ^1H NMR of Co(II)K(I) measured in CD_2Cl_2 15

Fig. S10 Evans NMR Method to determine the magnetic susceptibility of L_1Co(II)K(I)OAc 16

Fig. S11 ^1H NMR study of Co(III)K(I) in the presence of 1 or 2 equivalents of CHO at t = 0 min. 17

Fig. S12 ^1H NMR study of Co(III)K(I) in the presence of 1 or 2 equivalents of CHO at t = 18.5 h. 18

Fig. S13 DSC thermogram measured on a stoichiometric mixture of Co(III)K(I) + CHO held at an isotherm of 25 °C for 120 min, showing no reaction. 19

Fig. S14 DSC thermogram measured on a stoichiometric mixture of Co(II)K(I) + CHO held at an isotherm of 25 °C for 120 min. 19

Fig. S15 A DSC thermogram measured on a 1:4000 mixture of Co(III)K(I):CHO (1 mM Co(III)K(I), 4 M CHO, 40 mM mesitylene as internal standard in MeCN, with a V_{total} = 20 µL), held at an isotherm of 25 °C for 120 min. 20

Fig. S16 A. DSC thermogram measured on 1:4000 of Co(II)K(I) + CHO (1 mM Co(II)K(I), 4 M CHO, 40 mM mesitylene as internal standard in MeCN, with a V_{total} = 20 μL), held at an isotherm of 25 °C for 120 min. 21

UV-Vis Spectroscopy Binding Studies 22

Fig. S17 A. UV-Vis spectroscopy data obtained from the titration of L_1Co(III)K(I) with increasing equivalents of CHO. B. Plot showing the normalized change in extinction coefficient at λ = 361 nm (d→π* transition, filled squares) and at λ = 291 nm (π→π* transition, unfilled squares), which do not shift uniformly, precluding the determination of a 1:1 association constant. 22

Fig. S18 A. UV-Vis spectra obtained from the titration of L_1Co(III)K(I) with increasing equivalents of PO. B. Plot showing the normalized change in extinction coefficient at λ = 361 nm (d→π* transition, filled squares) and at λ = 291 nm (π→π* transition, unfilled squares), which do not shift uniformly, precluding the determination of a 1:1 association constant...... 23

Fig. S19 A. UV-Vis spectra obtained from the titration of L_1Co(III)K(I) with increasing equivalents of BO. B. Plot showing the normalized change in extinction coefficient at λ = 361 nm (d→π* transition, filled squares) and at λ = 291 nm (π→π* transition, unfilled squares), which do not shift uniformly, precluding the determination of a 1:1 association constant...... 24

Fig. S20 UV-Vis spectra obtained from the titration of L_1Co(II)K(I) with 200 – 700 equivalents CHO, demonstrating that no changes are observed upon the addition of 220 - 500 equivalents but the spectrum begins to change upon the addition of around 700 equivalents........ 25

Fig. S21 A. UV-Vis spectra obtained from the titration of Co(II)K(I) with increasing equivalents of BO. Increasing equivalents of epoxide are represented by changing colours from purple to blue to yellow to orange and red. B. Key region (300 – 500 nm) of the spectra shown in A. that were used to obtain the association constant K_1. C. Fitting of the UV-Vis data shown in A. K_1 was obtained using supramolecular.org/Bindfit/. The fit and all fitting parameters are accessible through the link listed in Table S2. 25

Fig. S22 A. UV-Vis spectra obtained from the titration of L1Co(II)K(I) with increasing equivalents of CPO........ 26

Fig. S23 A. UV-Vis spectra obtained from the titration of L_1Co(II)K(I) with increasing equivalents of AGE (0-1250 equiv.). 27

Fig. S24 UV-Vis spectra obtained by titrating L_1Co(II)K(I) with increasing concentrations of ^{t}BGE 28

Table S2 Links to the fits to the UV-Vis spectroscopy data, used to determined association constants, describing binding of epoxides to L_1Co(II)K(I) using supramolecular.org/Bindfit/. 29

Calculation of q for UV-vis studies........ 29

Fig. S25 Example plot of a semi-logarithmic plot of ln([epoxide]/[epoxide]$_0$) vs time, where k_{obs} is the slope of the plot, [cat]:[1,2-*trans* cyclohexane diol]:[epoxide] = 1:20:4000 (neat epoxide, 6 mL, 20 bar CO_2 pressure, 50 °C). 30

Table S3 Polymerization data for the polymerization of PO, CHO, BO, AGE, CPO and ^{t}BGE...... 31

Density Functional Theory Calculations 32

Computational Methods........ 32

Modelling of the initiation step of the copolymerization of (*R*)-PO and CO_2 catalyzed by L_1Co(III)K(I)(OAc)$_2$ 33

Table S4 Computed Free Gibbs Energies of intermediates and transition states for the first ring-opening of (R)-propylene by $L_1Co(III)K(I)(OAc)_2$ (see Fig S26). 33

Fig. S26 Illustration of the Free Gibbs energy surface for the initiation step and first transition state of the copolymerization of (R)-PO with CO_2 using the Co(III)K(I) catalyst ($L_1Co(III)K(I)(OAc)_2$), where A. Epoxide coordination occurs at the K(I) center, and B. Epoxide coordination occurs at the Co(III) center. 34

Modelling of the propagation step of the copolymerization of PO and CO_2 catalyzed by $L_1Co(III)K(I)$ 35

Table S5 Computed Free Gibbs Energies of intermediates and transition states for the ring-opening of (R)-propylene oxide by $L_1Co(III)K(I)(OAc)(O_2COR)$ (see Fig S27). 35

Fig. S27 Illustration of the Free Gibbs energy surface for a model propagation step of the copolymerization of (R)-PO with CO_2, and the (*R*)-PO ring-opening transition state, using the Co(III)K(I) catalyst ($L_1Co(III)K(I)(OAc)(O_2COR)$), where A. Epoxide coordination occurs at the K(I) center, and B. Epoxide coordination occurs at the Co(III) center. 36

Influence of the epoxide on the ring-opening activation barrier 37

Table S6 Computed Free Gibbs Energies of intermediates and transition states for the first ring-opening of epoxides by L_1 $Co(III)K(I)(OAc)_2$ (see Fig S28). 37

Fig. S28 Illustration of the Gibbs Free energy surface for the initiation step and first transition state of the copolymerization of epoxide with CO_2 using the Co(III)K(I) catalyst 38

Fig. S29 Correlation between the experimentally determined binding constant $K_{q=1}$ and the calculated initiation barrier to epoxide ring opening as illustrated in Fig. S28. 39

Fig. S30 Correlation between the experimentally determined polymerisation rate constant (k_p), normalized to epoxide concentration, and the calculated initiation barrier to epoxide ring opening as illustrated in Fig. S28. 39

Preliminary Cyclic Voltammetry Studies on Epoxide Binding to $L_1Co(III)K(I)$ 40

Fig. S31 A. CV of ferrocene, shown as background for B and C. B. CV of $L_1Co(III)K(I)$, C. CV of $L_1Co(III)$: All CVs were collected at approximately 1 mM analyte conditions, using 0.1 M $TBAPF_6$ in MeCN, scan rate = 0.1 V s^{-1}. 40

Fig. S32 Investigation of electrochemical behaviour of $L_1Co(III)K(I)$ with scan rate. A. Cyclic Voltammograms showing the decrease in peak distance for the Co(III/II) redox event with decreasing scan rate. B: Plot of the peak distance with decreasing scan rate. 41

Fig. S33 Voltammograms of $L_1Co(III)K(I)$ with increasing equivalents of epoxide: A. CHO, B. PO, C. AGE, D. BO. 41

Fig. S34 Plot showing the shift in E_{red} upon epoxide addition compared to $L_1Co(III)K(I)$ in the absence of any epoxide vs the binding constant, K_1, determined by UV-Vis spectroscopy. 42

Preliminary Study of Epoxide Binding to $L_2Co(III)K(I)$ 43

Synthesis and Characterization 43

Fig. S35 Synthesis of $L_2Co(II)K(I)$ and $L_2Co(III)K(I)$, where i) Ethylenediamine, $Co(II)OAc_2$, KOAc, MeCN (99% conversion, 38 % isolated yield (220 mg)) ii) 2 equivalents AcOH, MeCN, air (20 % isolated yield (120 mg)). 43

Fig. S36 UV-Vis spectrum of $L_2Co(II)K(I)$ (0.125 mM in MeCN). 43

Fig. S37 IR spectrum of $L_2Co(II)K(I)$. 44

Fig. S38 ^1H NMR spectrum of $L_2Co(II)K(I)$ in $CDCl_3$. 44

Fig. S39 Solid state structure of $L_2Co(II)K(I)$ obtained by single XRD. 45

Fig. S40 ^1H NMR spectrum of $L_2Co(III)K(I)$ in $CDCl_3$. 45

Fig. S41 $^{13}C\{^1H\}$ NMR spectrum of the previously reported ligand $L_2Co(III)K(I)$ in $CDCl_3$. 46

Fig. S42 IR spectrum of $L_2Co(III)K(I)$ 46

Fig. S43 UV-Vis spectra obtained by titrating $L_2Co(II)K(I)$ with increasing equivalents of A. CHO, C. AGE and E. ^tBGE 47

Fig. S44 UV-Vis spectra obtained by titrating $L_2Co(II)K(I)$ with increasing equivalents of A. PO, C. CPO and E. BO. 48

Table S7 Links to the fits to the UV-Vis spectroscopy data, used to determined association constants 49

Table S8 Polymerization data for the polymerization of PO, CHO, BO, AGE, CPO and ^tBGE. 50

Fig. S45 A. Exponential plot of k_p normalized to the neat concentration of each epoxide vs $K_{q=1}$ for epoxide/CO_2 ROCOP catalyzed by $L_2Co(III)K(I)$ 51

Crystallographic Details **52**

Table S9 Selected geometric parameters for $L_1Co(II)K(I)$ and $L_1Co(III)K(I)$. 52

Table S10 Summary of crystallographic refinement data for $L_1Co(II)K(I)$, $L_2Co(II)K(I)$ and $L_1Co(III)K(I)$ 53

References **56**

Materials and Methods

The spectroscopic data that supports the characterization of all complexes is available at Oxford University Research Archive, ORA, DOI: https://dx.doi.org/10.5287/ora-w4qq2ezmo.

All experimental manipulations were performed using a dual-manifold nitrogen-vacuum Schlenk line or in a nitrogen filled glovebox. All solvents and reagents were obtained from commercial sources and used as received, unless stated otherwise. Acetonitrile, pentane, toluene and chloroform were obtained from an SPS system, degassed by several freeze-pump-thaw cycles, further dried with 3 Å molecular sieves and stored under N_2. All epoxides were dried over two nights, over calcium hydride, and purified by fractional distillation, and stored under N_2. Research-grade CO_2 (BOC, CP grade, 99.995%) was dried by passing it through two drying columns (VICI Metronics carbon dioxide purifier) in series, at 50 bar pressure, before use at lower pressures in the co-polymerizations. Both di-aldehyde pro-ligands were obtained commercially from Manchester Organic and used as received.[1]

NMR spectroscopy were performed using a Bruker AV 500 MHz spectrometer, at 298 K, unless stated.

FT-IR measurements were performed using a Shimadzu IRSpirit spectrometer (installed inside the glove box) using a single reflection ATR accessory.

Gel permeation chromatography (GPC): was performed using a Shimadzu LC-20AD instrument, with two mixed bed PSS SDV linear S columns, in series, at 40 °C. THF was used as eluent, at a flow rate of 1 mL/min. Molar mass calibration was performed using narrow molar mass polystyrene standards.

CV studies were carried out using a Pine Wavedriver 10 potentiostat. Cyclic voltammetry experiments were performed, in a N_2 glovebox, using a three-electrode configuration, with an carbon disk (1.0 mm^2) as the working electrode, a glassy carbon rod as the counter electrode and an Ag wire as the pseudo-reference electrode. Experiments were performed using a sample solution, containing 0.1 M of tetrabutylammonium hexafluorophosphate as supporting electrolyte and the Co(III)K(I) catalyst (1 mM unless stated) in dry, degassed acetonitrile. Experiments were performed using a 100 mV s^{-1} scan rate, unless otherwise state. All experiments were performed in the presence of ferrocene (ca 1 mM) as internal standard.

UV-Vis spectroscopy studies were conducted using an Ocean optics FLAME-S-UV-VIS spectrometer, connected to an Ocean optics DH-2000-BAL UV-VIS-NIR light source, using a 1 cm quartz cuvette, at room temperature. Measurements were taken inside a nitrogen filled glovebox, using an ocean optics cuvette holder. All UV-Vis spectroscopy experiments were conducted on sample solutions, with a starting concentration of 0.125 mM analyte in acetonitrile, obtained by addition of stock solutions to 2 mL acetonitrile (C_{stock}(Co(II)K(I)) = 0.025 M, C_{stock}(Co(III)K(I)) = 0.01 M in acetonitrile). Titration experiments were conducted using 5 M stock solutions of epoxides, except in the case of titrations using ^{t}BGE and CHO below 1000 equiv., which were conducted using 1 M epoxide stock solutions. All additions were conducted using Hamilton micro syringes. All peaks were picked in OriginPro 2024b, using the screen reader tool.

Differential scanning calorimetry (DSC) was performed using a TA Discovery 25-Auto. DSC measurements were performed in hermetic DSC pans, prepared in a nitrogen filled glovebox according to a previously reported method[2]: All reaction solutions (0.1 mL) were prepared from stock solutions of Co(III)K(I) (0.04 M), Co(II)K(I) (0.04 M) and CHO (4 M for experiments with excess CHO and 0.1 M for stoichiometric experiments) in acetonitrile. Mesitylene was added as internal standard to all reactions. For each DSC measurement, 20 μL of the reaction solution was transferred into the pre-weighed DSC pan, which was sealed in the glovebox and subsequently

transferred to the DSC instrument. For isothermal measurements, the DSC cell was heated to the reaction temperature, before automated loading of the reference pan and sample pan. After the experiment, pans were recovered and reweighed to confirm no weight loss had occurred. Subsequently, each pan was pierced with a needled and the entire residual was dissolved in $CDCl_3$ to obtain a sample for NMR spectroscopy.

Elemental Analysis was carried out by the London Metropolitan University (166-220 Holloway Road, London, N7 8DB). Elemental Analysis of $L_2Co(II)K(I)$ was carried out by the Microanalysis Team at the University of York.

General Epoxide/CO_2 ROCOP Procedure

A solution of catalyst, *trans*-1,2-cyclohexane diol and mesitylene (internal standard) in 6 mL of neat epoxide, where [catalyst]:[*trans*-1,2-cyclohexane diol]:[epoxide] = 1:20:4000, was prepared in a nitrogen filled glovebox. Under a stream of dry CO_2, the prepared solution was injected into a 100 mL Parr reactor, fitted with a DiComp sentinel probe, attached to an ATR-IR spectrometer. The CO_2 pressure was controlled using a Bronkhorst EP-Flow select Mass Flow Meter, which was set to 20 bar CO_2 for all reactions. After pressurization the reactor was heated to 50 °C. Throughout the experiment, the reaction was left open to the CO_2 line and a constant pressure of 20 bar CO_2 was maintained through automated CO_2 injections by the Flow Meter. Using the *in situ* IR spectrometer, the reaction was monitored using the poly(carbonate) signal at 1750 cm^{-1}. Upon reaction completion, the reactor vessel was cooled to room temperature and depressurized. A 1 M solution of benzoic acid in $CHCl_3$ was used to quench the catalyst, and an aliquot of the crude reaction mixture was removed for ^1H NMR spectroscopy and GPC analysis.

Synthesis of the ligand (H_2L_1)[1]

H_2L_1 was prepared by a literature procedure[1] by the condensation of 2-hydroxy-3-(2-methoxyethoxy)benzaldehyde (1000 mg, 5.2 mmol) with ethylene diamine (186 µL, 2.56 mmol). The 2-hydroxy-3-(2-methoxyethoxy)benzaldehyde was dissolved in the minimum volume of MeOH (10 mL) and ethylene diamine was added. The bright yellow solution was left to stir for an hour, or until H_2L_1 was formed as a yellow precipitate. The solution was filtered and the precipitate was dried under high vacuum for 16 h, affording H_2L_1 as a yellow solid in good yield (1920 mg, 90 % yield).

^1H NMR (500 MHz, CD_2Cl_2) δ 13.46 (s, 2H, O-<u>H</u>, i), 8.37 (s, 2H, N=C-<u>H</u>, b), 6.92 (ddd, *J* = 19.5, 7.9, 1.5 Hz, 4H, Ar-$\underline{H}_{m}$, c, e), 6.77 (t, *J* = 7.9 Hz, 2H, Ar-$\underline{H}_{p}$, d), 4.20 - 4.07 (m, 4H, CH_3-O-$C\underline{H}_2$, g), 3.95 (s, 4H, $C\underline{H}_2$-O-CH_2, f), 3.7980 – 3.70 (m, 4H, N-$C\underline{H}_2$, a), 3.41 (s, 6H, $C\underline{H}_3$, h). ^{13}C NMR (126 MHz, CD_2Cl_2) δ 167.10 (N=C, b), 152.29 (Ar-<u>C</u>-OH, h), 147.82 (Ar-C_{ortho}, c) , 124.15 (Ar-C_{meta}, d/f), 119.36 (Ar-$\underline{C}_{ortho}$-O-CH_2, g) 118.35 (Ar-$\underline{C}_{para}$-H, e), 116.97 (Ar-C_{meta}, d/f), 71.32 (Ar-O-$\underline{C}H_2$, i), 68.94 (CH_2-$\underline{C}H_2$-O, j), 59.97 (N-$\underline{C}H_2$, a), 59.22 ($\underline{C}H_3$, k)

L_1Co(II)K(I) synthesis

Under an inert N_2 atmosphere, H_2L_1 (500 mg, 1.2 mmol), KOAc (118 mg, 1.2 mmol) and $Co(OAc)_2$ (213 mg, 1.2 mmol) were stirred in dry acetonitrile (10 mL) for 6 h. The solvent was then removed *in vacuo* and the six azeotropic washes were performed (toluene (3x 10 mL), pentane (3x 10 mL)). The resulting red solid was precipitated from dichloromethane using pentane (10 mL), under an inert N_2 atmosphere to afford Co(II)K(I). The complex was then dried under high vacuum for 16 h to afford Co(II)K(I) as an orange / light red powder (isolated yield: 180 mg, 30 % yield). v_{max}/cm^{-1} 2904 cm^{-1} (C(sp^2)-H), 1635 cm^{-1}(C=N). EA found: C 48.26, H: 5.13, N: 4.43, which corresponds to $(Co(II)K(I))_4 \cdot (CHCl_3)$ which is calculated as: C: 48.43, H: 4.90, N: 4.66. EA was performed on a crystalline sample, in which Co(II)K(I) co-crystalised with $CHCl_3$.

L_1Co(III)K(I) synthesis[1]

Co(III)K(I) was synthesized according to the previously reported literature procedure.[1] Under an inert N_2 atmosphere, H_2L_1 (1000 mg, 2.4 mmol), KOAc (235 mg, 2.4 mmol) and $Co(OAc)_2$ (425 mg, 2.4 mmol) were stirred in dry acetonitrile (10 mL) for 16 h. The solution was then opened to air and acetic acid (247 µL, 4.8 mmol) were added. The solution was stirred for 16 hour in air, and the solvent was removed under vacuum. Six azeotropic washes were performed ((toluene (3x 10 mL), pentane (3x 10 mL)), and Co(III)K(I) was precipitated from dichloromethane using pentane

(20 mL). The resulting brown powder was dried under high vacuum to afford Co(III)K(I) in good yields (870 mg, 57 % yield).

^{1}H NMR (500 MHz, CD_2Cl_2) δ 7.73 (s, 2H, N=C-$\underline{H}$, b), 6.90 (dd, *J* = 7.9 Hz, 1.6 Hz, 2H, Ar-$\underline{H}_{m,}$ c/e), 6.82 (dd, *J* = 7.7 Hz, 1.6 Hz, 2H, Ar-$\underline{H}_{m,}$ c/e), 6.46 (t, *J* = 7.8 Hz, 1H, Ar-$\underline{H}_{p,}$ d), 4.28 (s, 4H, N-C$\underline{H}_2$, a), 4.23 – 4.17 (m, 4H, C$\underline{H}_2$-O-CH_2, f), 3.91 – 3.86 (m, 4H, CH_3-O-C$\underline{H}_2$, g), 3.50 (s, 6H, C$\underline{H}_3$, h), 1.37 (s, 6H, O-Ac, i). ^{13}C NMR (126 MHz, $CDCl_3$) δ 178.58 (C=O (OAc), l), 165.11(N=C, b), 157.62 (Ar-$\underline{C}$-OH, g), 152.29 (Ar-$\underline{C}$-O, h) 126.76 (Ar-$\underline{C}_{ortho}$-O-CH_2, h), 119.46 (Ar-C_{meta}, d/f), 114.53 (Ar-C_{ortho}, c), 112.70 (Ar-C_{meta}, d/f), 71.04 (CH_2-$\underline{C}H_2$-O, j), 66.97 (Ar-O-$\underline{C}H_2$, i), 59.52 (N-$\underline{C}H_2$, a), 58.51 ($\underline{C}H_3$, k), , 24.66 ($H_3\underline{C}$-C=O (OAc), l). v_{max}/cm^{-1} 2914 (C(sp^2)-H). EA: Found: C: 48.51, H: 5.08, N: 4.19, which corresponds to $(Co(III)K(I))_6\cdot(CHCl_3)$, calculated as: C: 48.32, H: 4.98, N: 4.31. The EA was run on a crystalline sample and the co-crystallisation of $CHCl_3$.is consistent with the structure obtained for Co(III)K(I) (see XRD section, local code: 049ke24).

L_2Co(II)K(I) synthesis

Under an inert N_2 atmosphere, the benzaldehyde precursor ligand (400 mg, 1.0 mmol), KOAc (100 mg, 1.0 mmol) and $Co(OAc)_2$ (181 mg, 1.0 mmol) were stirred in dry acetonitrile (5 mL) for 1 h. Subsequently, ethylene diamine was added (68 µL, 1.0 mmol), and the solution was stirred for 16 hours. The solvent was removed under vacuum. Six azeotropic washes were performed under an inert N_2 atmosphere ((toluene (3x 5 mL), pentane (3x 5 mL)), and L_2Co(II)K(I) was precipitated from dry dichloromethane using dry pentane (10 mL). The resulting red/orange powder was dried under high vacuum to afford L_2Co(III)K(I) (220 mg, 30 % yield). EA found: C 48.78, H: 5.15, N: 4.39, which corresponds to Co(II)K(I)·(H_2O) which is calculated as: C: 49.06, H: 4.98, N: 4.77.

L_2Co(III)K(I) synthesis[3]

L_2Co(III)K(I) was synthesized according to the previously reported literature procedure.[3] Under an inert N_2 atmosphere, the benzaldehyde precursor ligand (400 mg, 1.0 mmol), KOAc (100 mg, 1.0 mmol) and $Co(OAc)_2$ (181 mg, 1.0 mmol) were stirred in dry acetonitrile (5 mL) for 1 h. Subsequently, ethylene diamine was added (68 µL, 1.0 mmol), and the solution was stirred for 16 hours. The solution was then opened to air and acetic acid (119 µL, 1.0 mmol) was added. The solution was stirred for 16 hour in air, and the solvent was removed under vacuum. Six azeotropic washes were performed ((toluene (3x 5 mL), pentane (3x 50 mL)), and L_2Co(III)K(I) was precipitated twice from dichloromethane using pentane (20 mL). The resulting brown powder was dried under high vacuum to afford Co(III)K(I) (120 mg, 20 % yield).

^{1}H NMR (500 MHz, $CDCl_3$) δ 7.70 (s, 2H, N=C-$\underline{H}$, b), 6.85 (dd, *J* = 8.0, 1.5 Hz, 2H, Ar-$\underline{H}_{m,}$ c/e), 6.69 (dd, *J* = 7.7, 1.5 Hz, 2H, Ar-$\underline{H}_{m,}$ c/e), 6.40 (t, *J* = 7.8 Hz, 2H, Ar-$\underline{H}_{p,}$ d), 4.30 (s, 4H, N-C$\underline{H}_2$, a), 4.23 – 4.14 (m, 4H, C$\underline{H}_2$-O-CH_2, f), 3.97 – 3.88 (m, 4H, CH_3-O-C$\underline{H}_2$, g), 3.80 (s, 4H, C$\underline{H}_3$, h), 1.45 (s, 6H, O-Ac, i).^{13}C NMR (126 MHz, $CDCl_3$) δ 179.55 (C=O (OAc), l), 164.72 (N=C, b), 157.30 (Ar-$\underline{C}$-OH, g), 152.17 (Ar-$\underline{C}$-O, h), 126.10 (Ar-$\underline{C}_{ortho}$-O-CH_2, h), , 119.11 (Ar-C_{meta}, d/f), 112.64, (Ar-C_{ortho}, c), 112.35 (Ar-C_{meta}, d/f), 70.34 (CH_2-$\underline{C}H_2$-O, j), 69.77 (Ar-O-$\underline{C}H_2$, i), 66.02 (N-$\underline{C}H_2$, a), 59.32 ($\underline{C}H_3$, k), 24.86 ($H_3\underline{C}$-C=O (OAc), l). v_{max}/cm^{-1} 2909 (C(sp^2)-H).

Additional Information

Table S1 Data for the bar chart shown in Fig. 2.

Catalyst	Epoxide	Temperature (°C)	Pressure (bar)	$TOF_{Polycarbonate}$ $(h)^{-1}$	$TOF_{Normalized}$ (h^{-1})
Cr(III)SalenCl (Catalyst A)[4a] [catalyst A]:[PPN_3]:[epoxide] = 1:2:500.	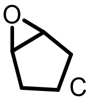 CPO	80	35	0	0
	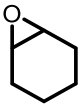 CHO	80	35	205	1
Fe(III)Corrole (Catalyst B) [5b] [catalyst B]:[PPNCl]:[epoxide] = 1:0.5:4000.	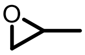 PO	60	20	171	1
	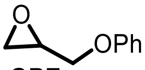 GPE	60	20	36	0.4
Organoboron (Catalyst C)[6c] [catalyst C]:[PPNCl]:[epoxide] = 1:1:500	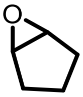 CPO	25	20	3	0.1
	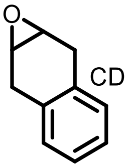 CDO	25	20	1	0.03
	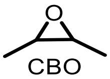 CBO	25	20	12	0.4
	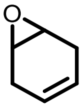 CEO	25	20	4	0.13
	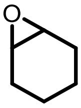 CHO	25	20	30	1
Al(III)porphyrin (Catalyst D)[7d] [catalyst D]: [PPNCl]:[epoxide] = 1:0.5:10000	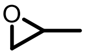 PO	70	40	3200	1
	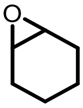 CHO	70	40	490	0.15
	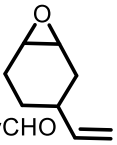 vCHO	70	40	350	0.11

	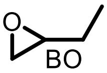 BO	70	40	1300	0.39
	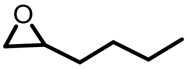 HO	70	40	870	0.26
	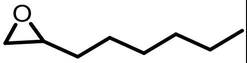 OO	70	40	720	0.21

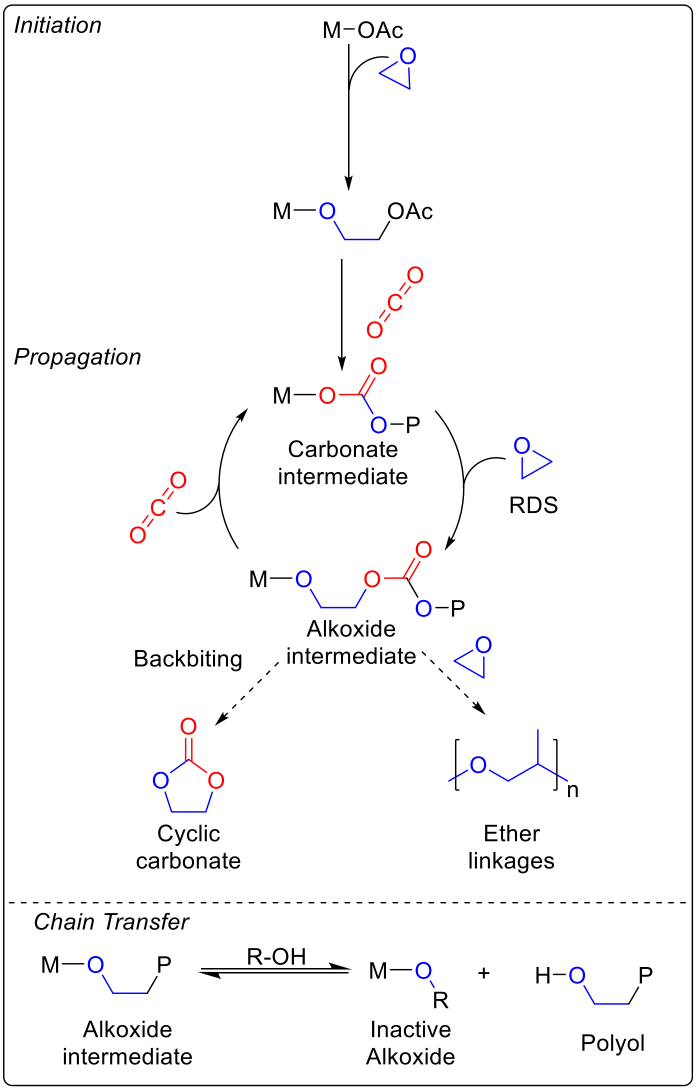

Fig. S2 General mechanism of epoxide/CO_2 ROCOP for a metal acetate catalyst, including initiation and chain transfer.

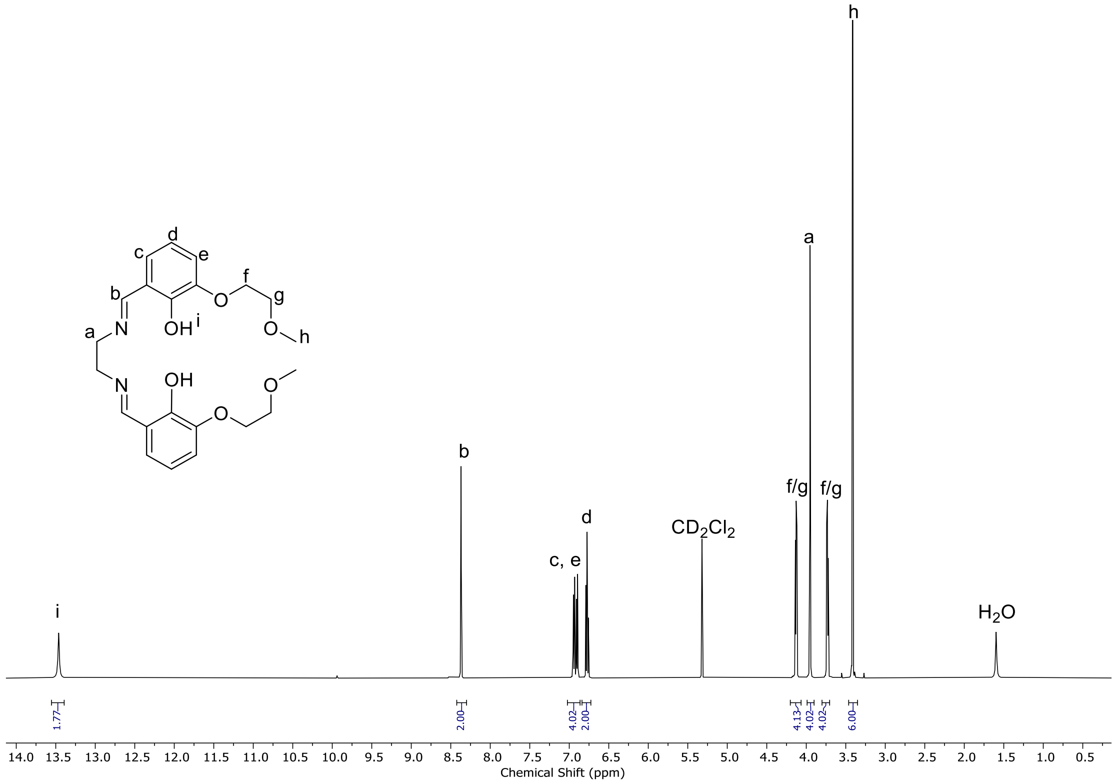

Fig. S3 ^{1}H NMR spectrum of the previously reported ligand H_2L_1 reported in CD_2Cl_2.

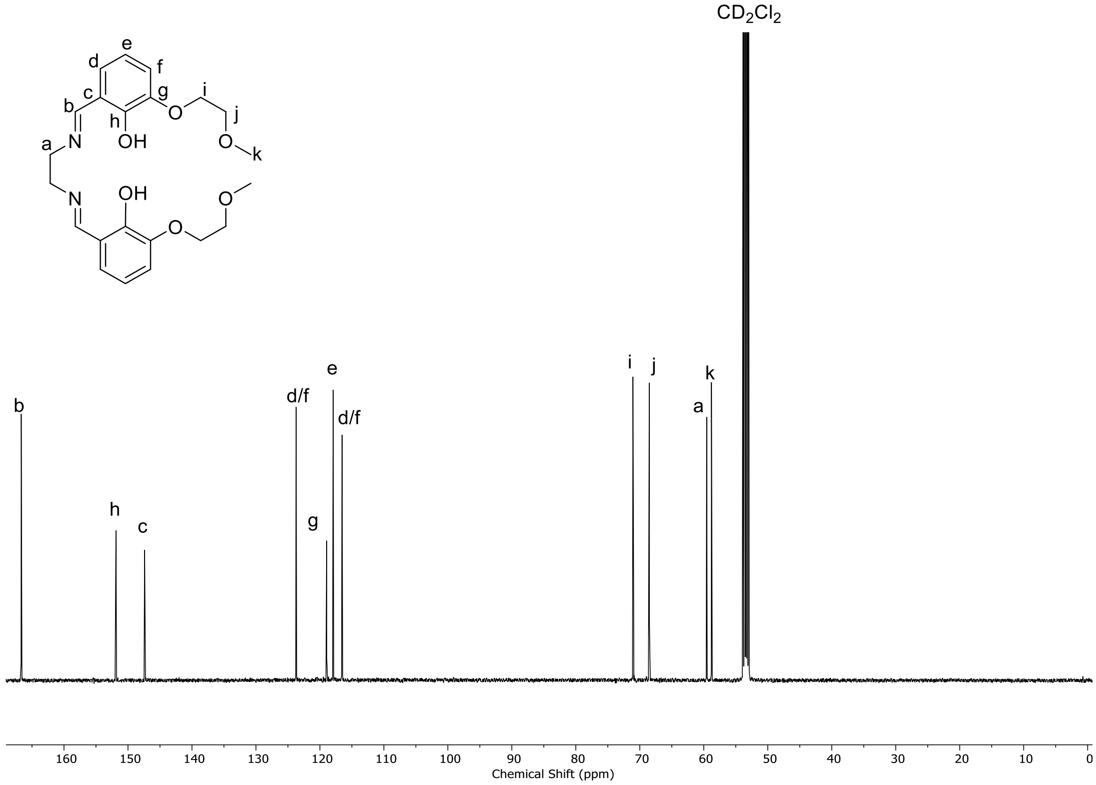

Fig. S4 $^{13}C\{^{1}H\}$ NMR spectrum of the previously reported ligand H_2L_1 recorded in CD_2Cl_2.

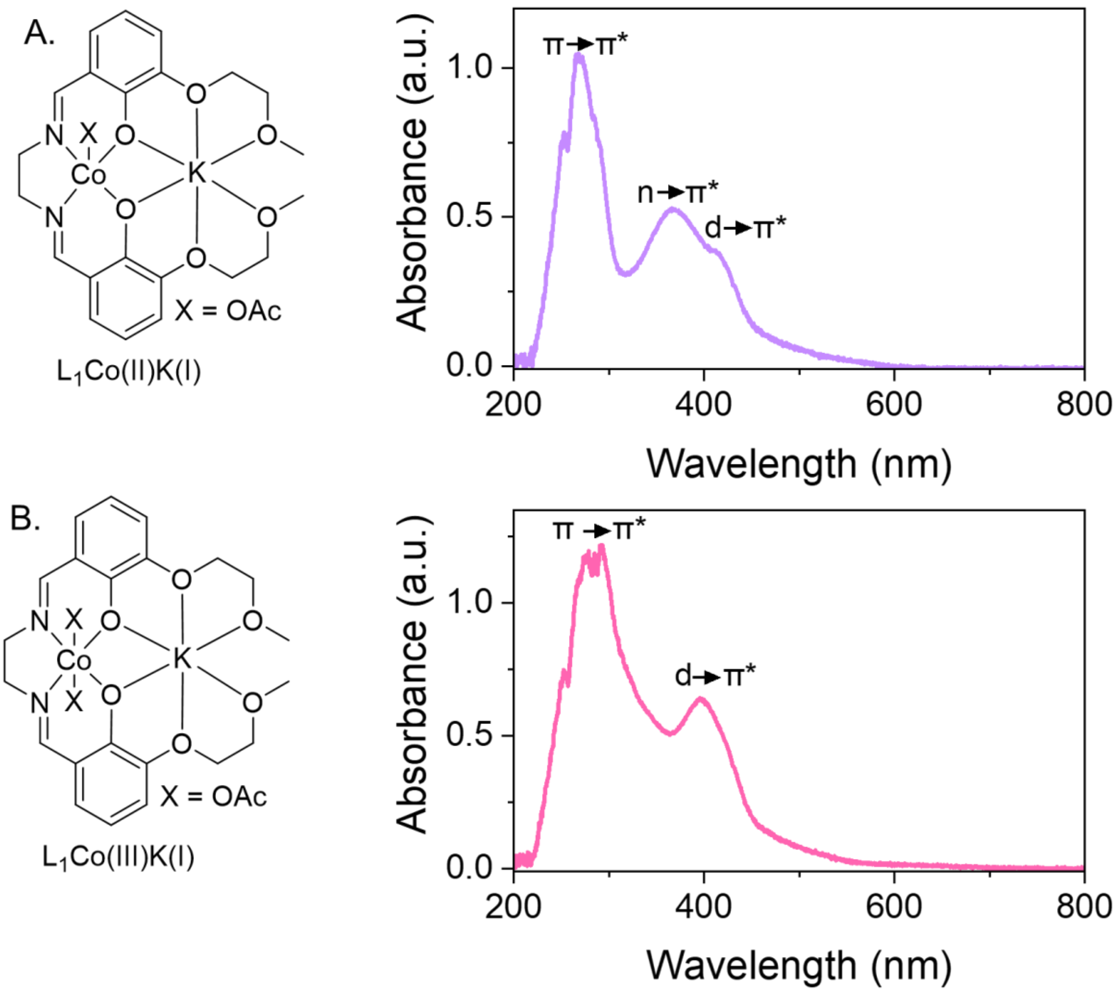

Fig. S5 UV-Vis spectra of A. Co(II)K(I) and B. Co(III)K(I). Spectra were collected at 0.125 mM catalyst, in MeCN, under an inert N_2 atmosphere in a glovebox.

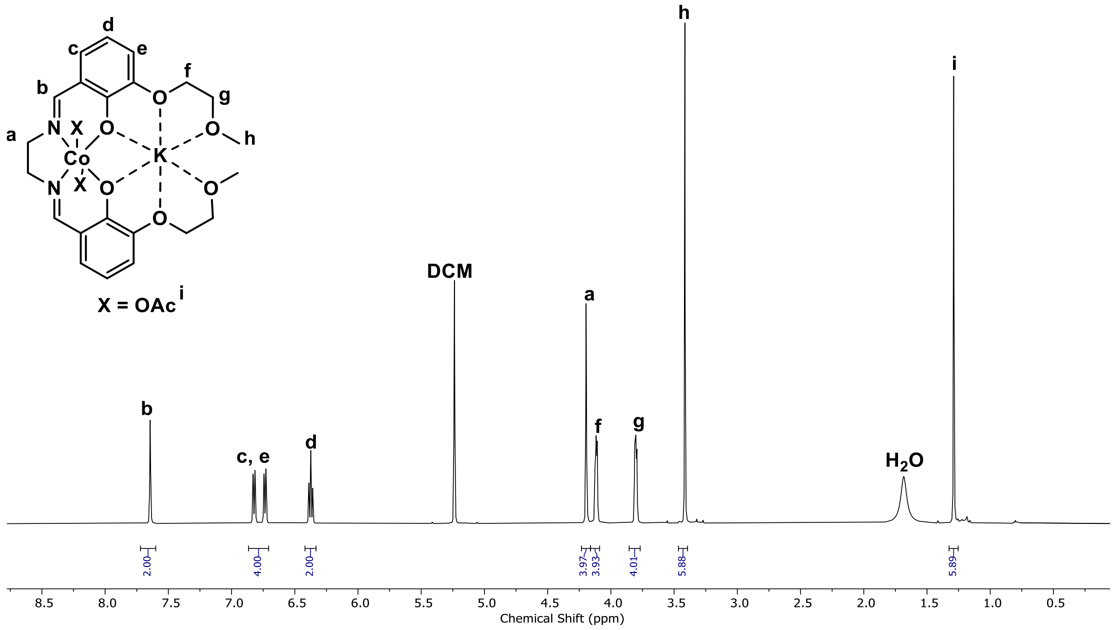

Fig. S6 ^{1}H NMR spectrum of Co(III)K(I) measured in CD_2Cl_2.

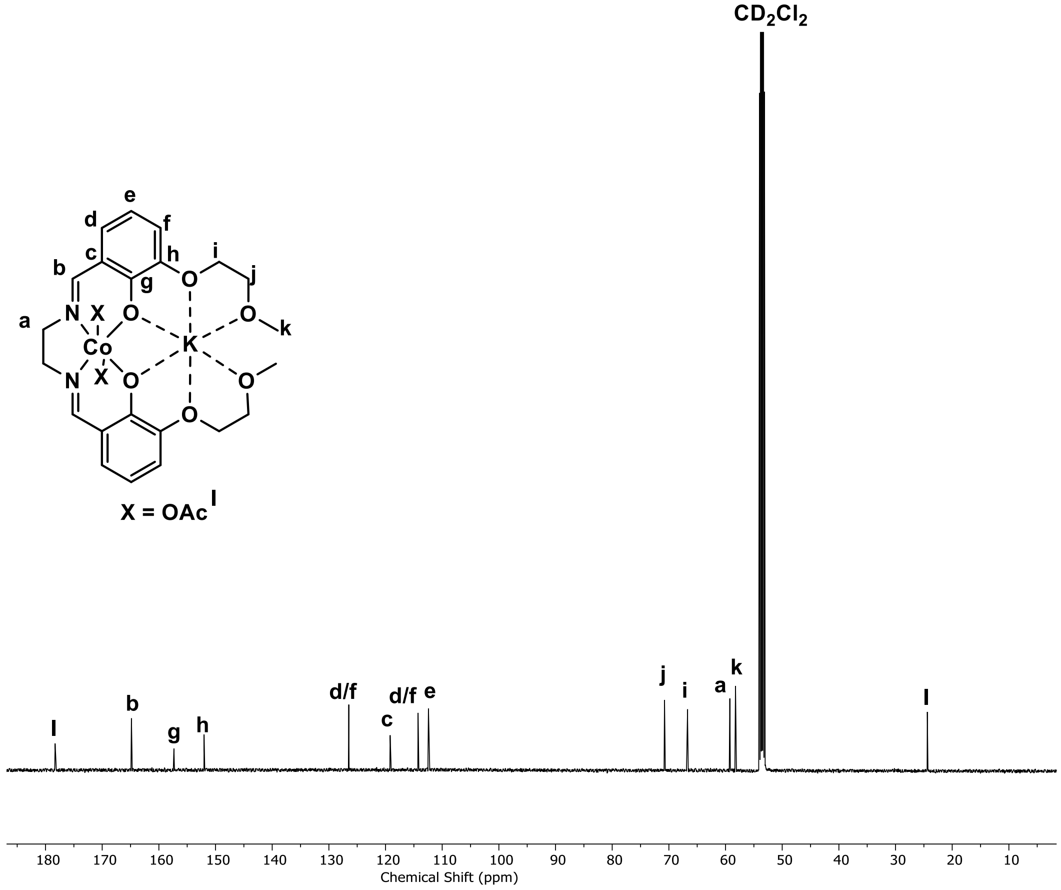

Fig. S7 ^{13}C{^{1}H} NMR of Co(III)K(I) measured in CD_2Cl_2

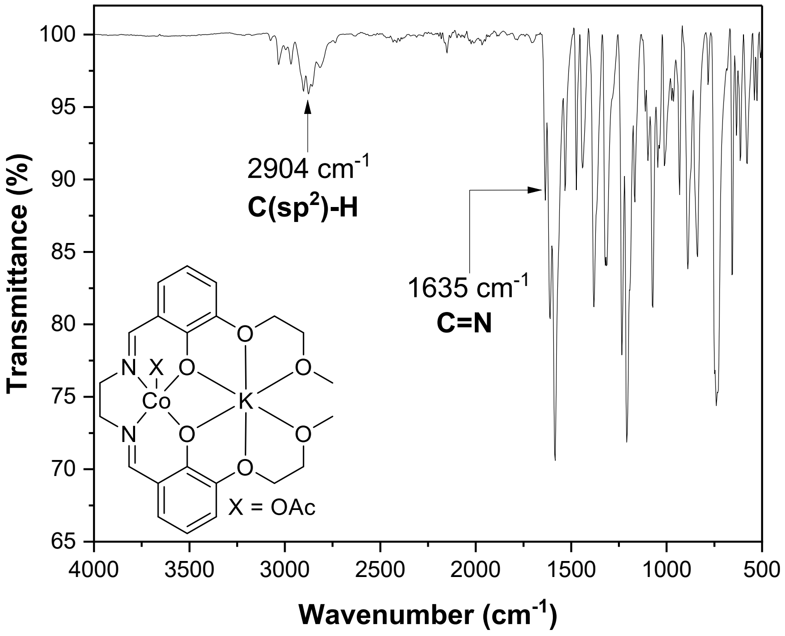

Fig. S8 IR spectrum of the Co(II)K(I) complex.

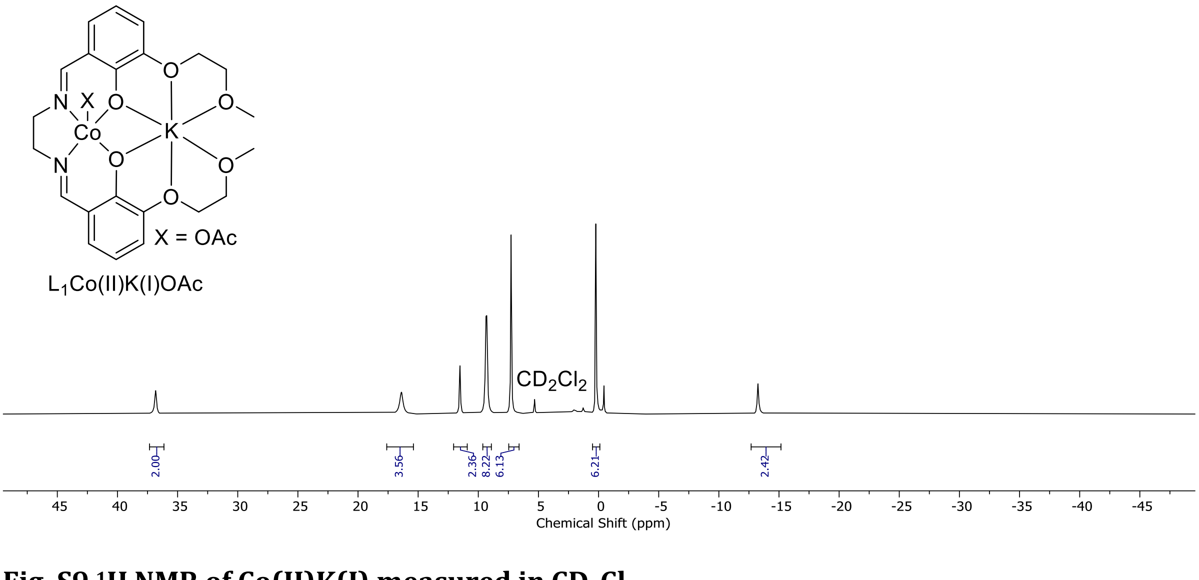

Fig. S9 ^{1}H NMR of Co(II)K(I) measured in CD_2Cl_2

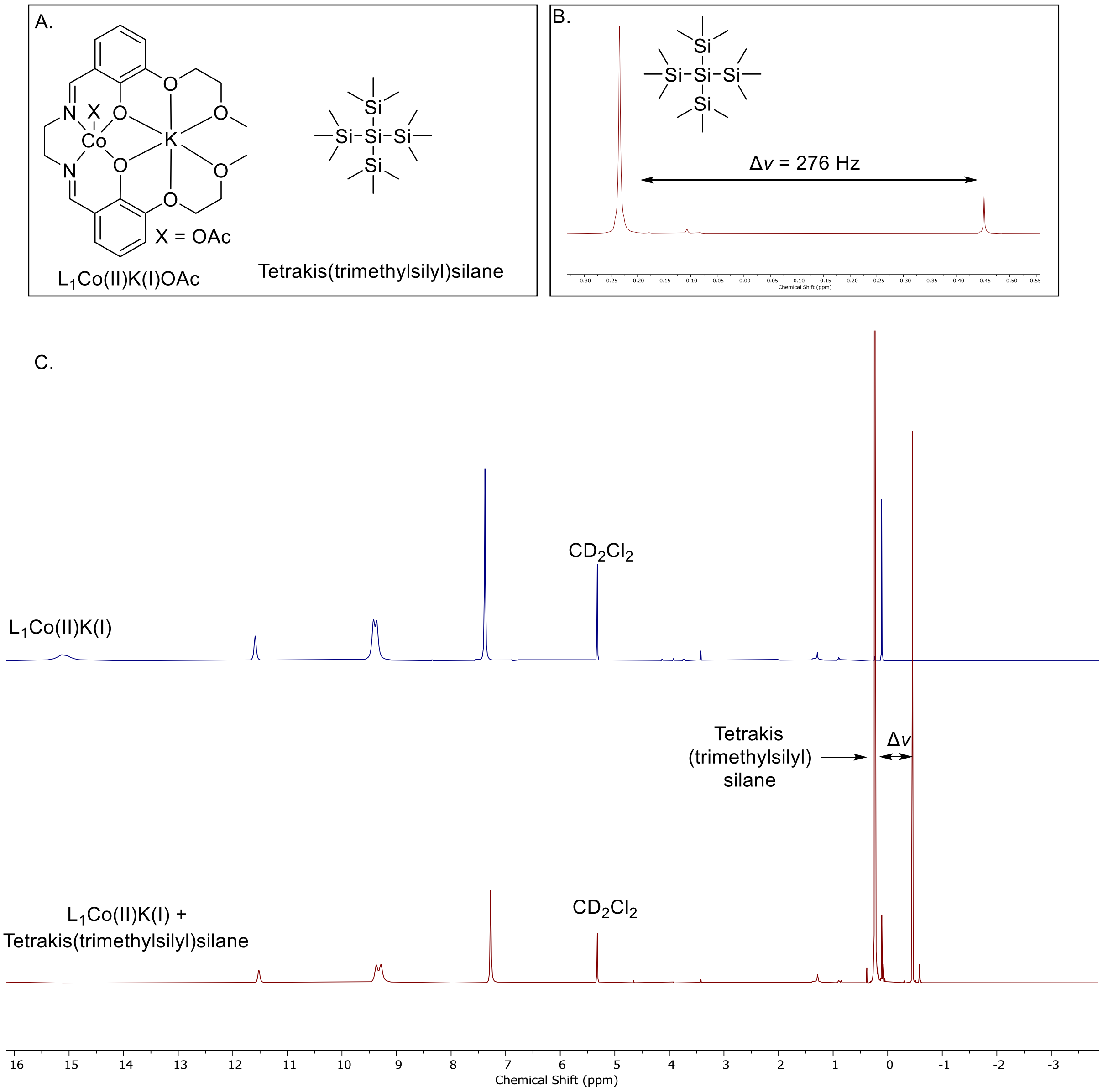

Fig. S10 Evans NMR Method to determine the magnetic susceptibility of $L_1Co(II)K(I)OAc$. A. Structure of $L_1Co(II)K(OAc)$ and tetrakis(trimethylsilyl)silane, which was used as the internal standard. B. Key region of the ^1H NMR spectrum used to determine the magnetic susceptibility of $L_1Co(II)K(OAc)$: The signal for tetrakis(trimethylsilyl)silane is shifted by 276 Hz in the presence of $L_1Co(II)K(OAc)$. The spectrum was obtained by inserting a sealed innertube containing tetrakis(trimethylsilyl)silane in CD_2Cl_2 into a Young's tab NMR tube containing $L_1Co(II)K(OAc)$ in CD_2Cl_2.[8] The molar magnetic susceptibility of $L_1Co(II)K(I)OAc$ was determined using $\boldsymbol{X_M} = \frac{\Delta v}{S_f v_0} \times \frac{1000}{c}$, where $\boldsymbol{\Delta v}$ is the shift of tetrakis(trimethylsilyl)silane in the presence of $L_1Co(II)K(OAc)$, S_f is assumed to be $4\boldsymbol{\pi}/3$, v_0 is the frequency of the NMR spectrometer (400 Hz), and c is the analyte concentration, c($L_1Co(II)K(I)$ = 0.02 M). C. Top: ^1H NMR spectrum of $L_1Co(II)KOAc$ in CD_2Cl_2 shown as a reference. Bottom: Full ^1H NMR spectrum of tetrakis(trimethylsilyl)silane in the presence of $L_1Co(II)K(OAc)$ in CD_2Cl_2. The region shown in part B. was taken from the bottom spectrum shown in C.

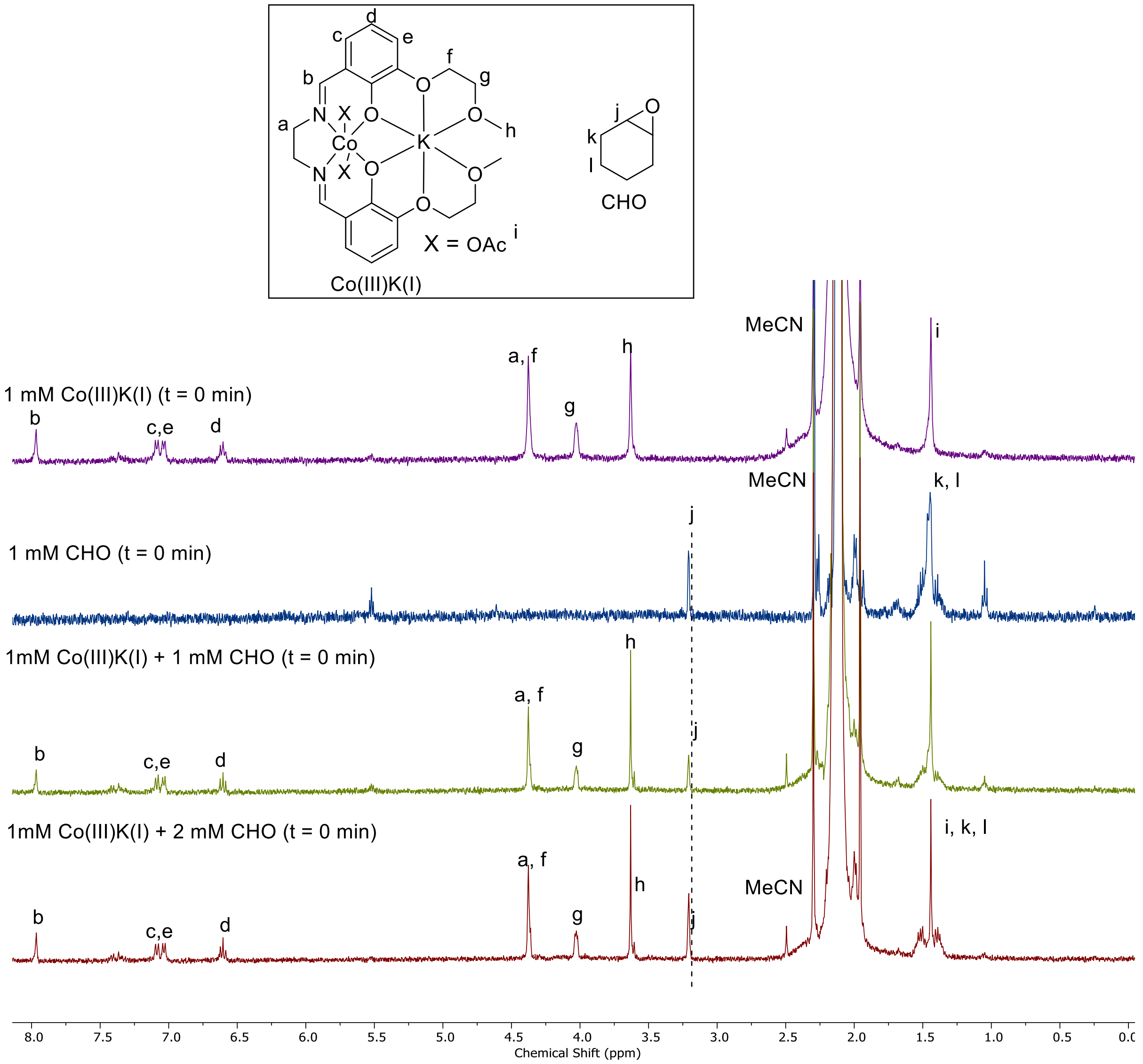

Fig. S11 ^{1}H NMR study of Co(III)K(I) in the presence of 1 or 2 equivalents of CHO at t = 0 min.

Stacked ^{1}H NMR spectra of free Co(III)K(I) and CHO as a control to illustrate the shift unreacted epoxide. The spectra obtained straight after mixing of 1:1 and 1:2 catalyst with epoxide illustrate that no reaction occurs, as peaks remain unshifted compared to the pure Co(III)K(I) and pure CHO spectra.

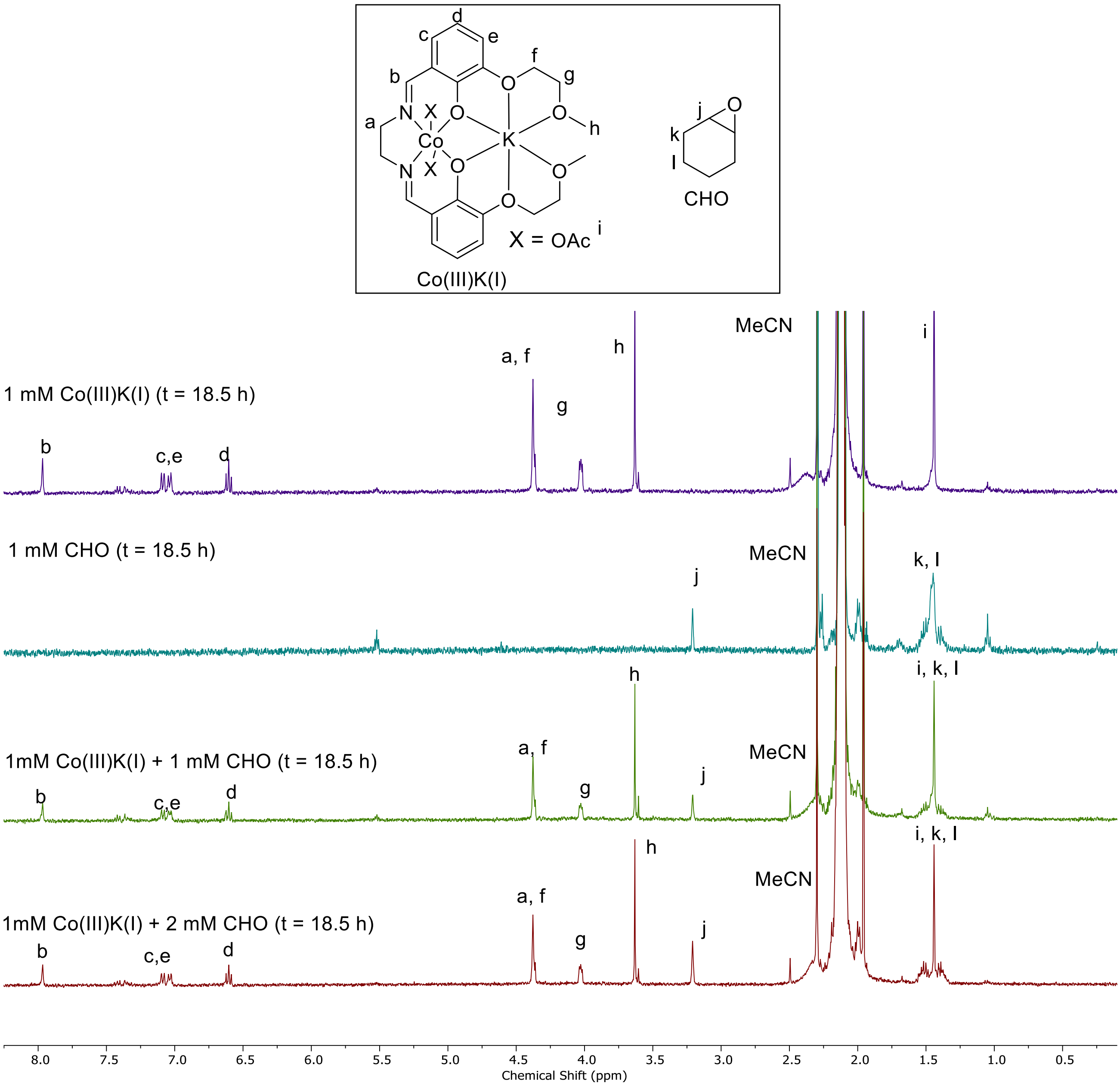

Fig. S12 ^{1}H NMR study of Co(III)K(I) in the presence of 1 or 2 equivalents of CHO at t = 18.5 h.

Stacked ^{1}H NMR spectra of free Co(III)K(I) and CHO after 18. 5 h. The spectra obtained of 1:1 and 1:2 catalyst with epoxide mixtures after 18.5 h illustrate that no reaction occurs, as peaks remain unshifted compared to the pure Co(III)K(I) and pure CHO spectra

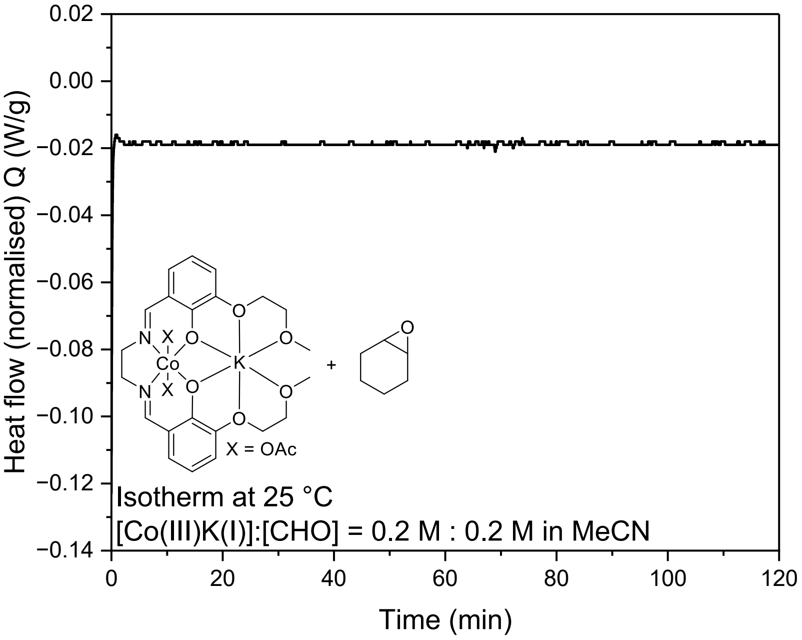

Fig. S13 DSC thermogram measured on a stoichiometric mixture of Co(III)K(I) + CHO held at an isotherm of 25 °C for 120 min, showing no reaction.
The weight of the DSC pan was determined before and after the experiment to ensure that no evaporation occurred ($m_{\text{pre experiment}}$ = 6.62 mg, $m_{\text{post experiment}}$ = 6.62 mg).

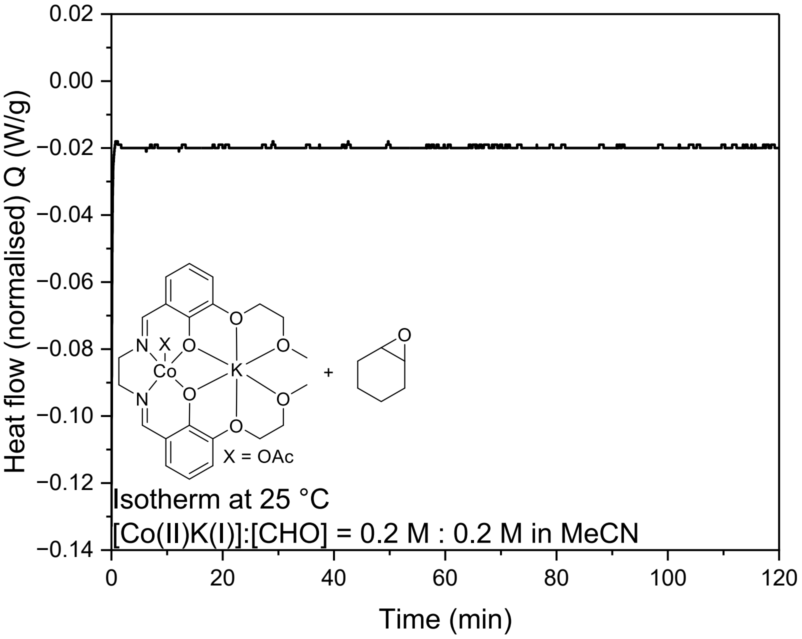

Fig. S14 DSC thermogram measured on a stoichiometric mixture of Co(II)K(I) + CHO held at an isotherm of 25 °C for 120 min.
The weight of the DSC pan was determined before and after the experiment to ensure that no evaporation occurred ($m_{\text{pre experiment}}$ = 5.69 mg, $m_{\text{post experiment}}$ = 5.62 mg).

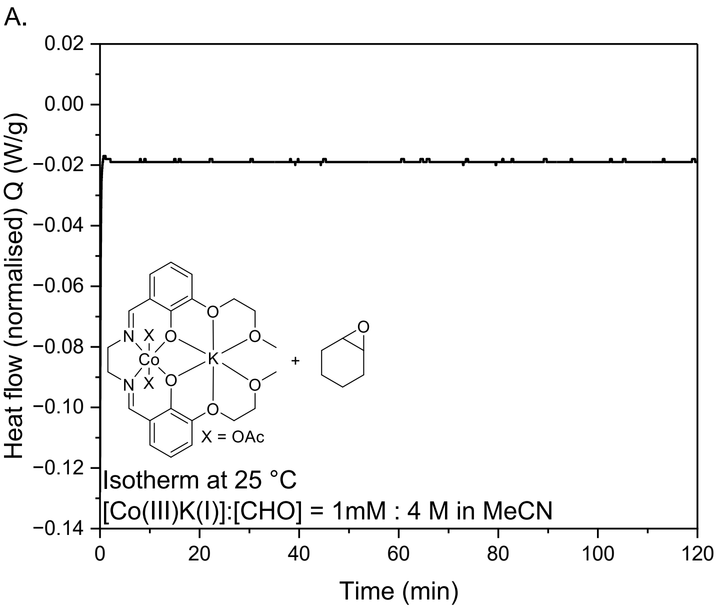

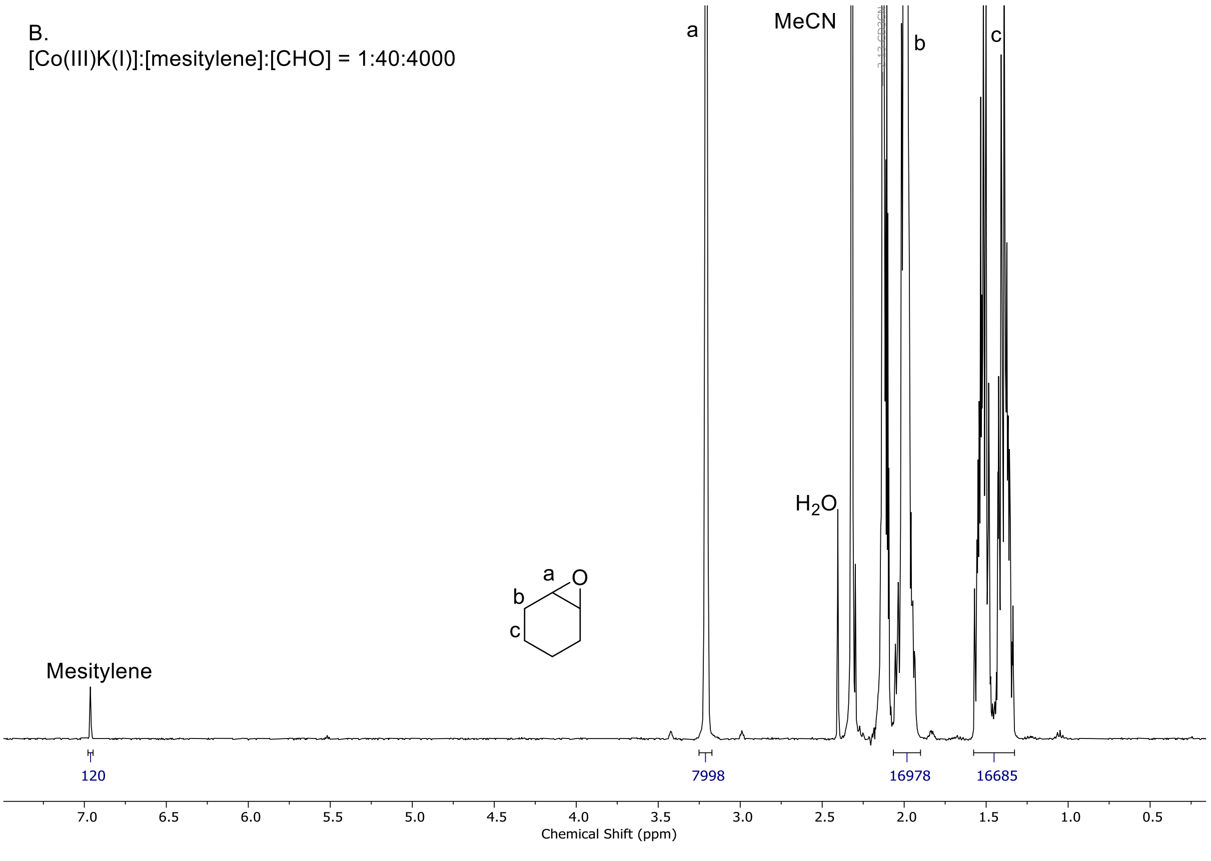

Fig. S15 A DSC thermogram measured on a 1:4000 mixture of Co(III)K(I):CHO (1 mM Co(III)K(I), 4 M CHO, 40 mM mesitylene as internal standard in MeCN, with a V_{total} = 20 µL), held at an isotherm of 25 °C for 120 min.

The DSC pan was weighed before and after the experiment to ensure a constant weight was maintained ($m_{pre\ experiment}$ = 6.70 mg, $m_{post\ experiment}$ = 6.69 mg). B. ^{1}H NMR spectrum of the crude reaction mixture, showing that all initially equivalents of CHO added (4000 equivalents), remain unreacted (vs a mesitylene standard).

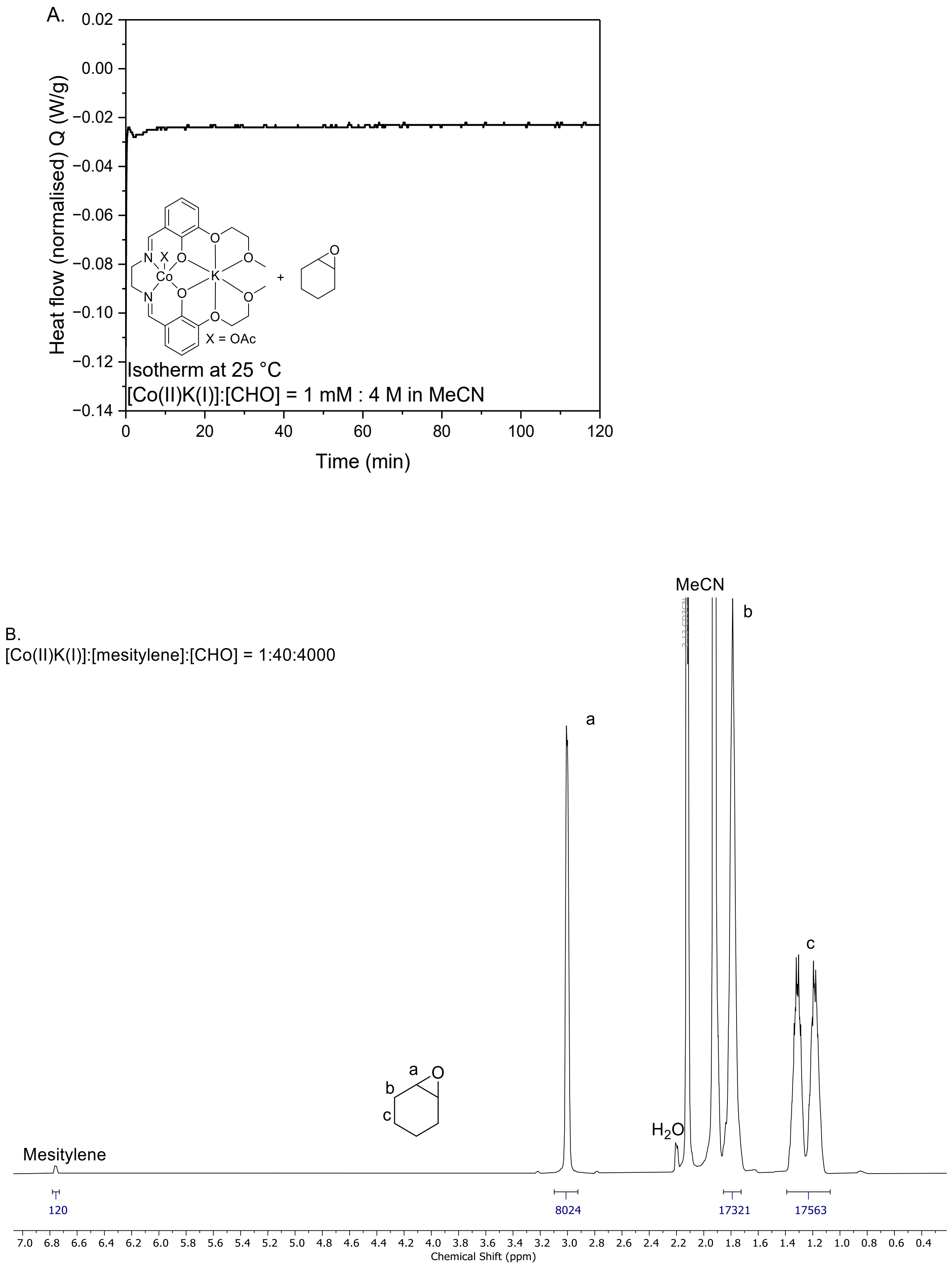

Fig. S16 A. DSC thermogram measured on 1:4000 of Co(II)K(I) + CHO (1 mM Co(II)K(I), 4 M CHO, 40 mM mesitylene as internal standard in MeCN, with a V_{total} = 20 µL), held at an isotherm of 25 °C for 120 min.

The DSC pan was weighed before and after the experiment to ensure a constant weight was maintained ($m_{pre\ experiment}$ = 6.99 mg, $m_{post\ experiment}$ = 7.00 mg). B. ^{1}H NMR spectrum of the crude reaction mixture, showing that all initially equivalents of CHO added, remain unreacted (vs a mesitylene standard).

UV-Vis Spectroscopy Binding Studies

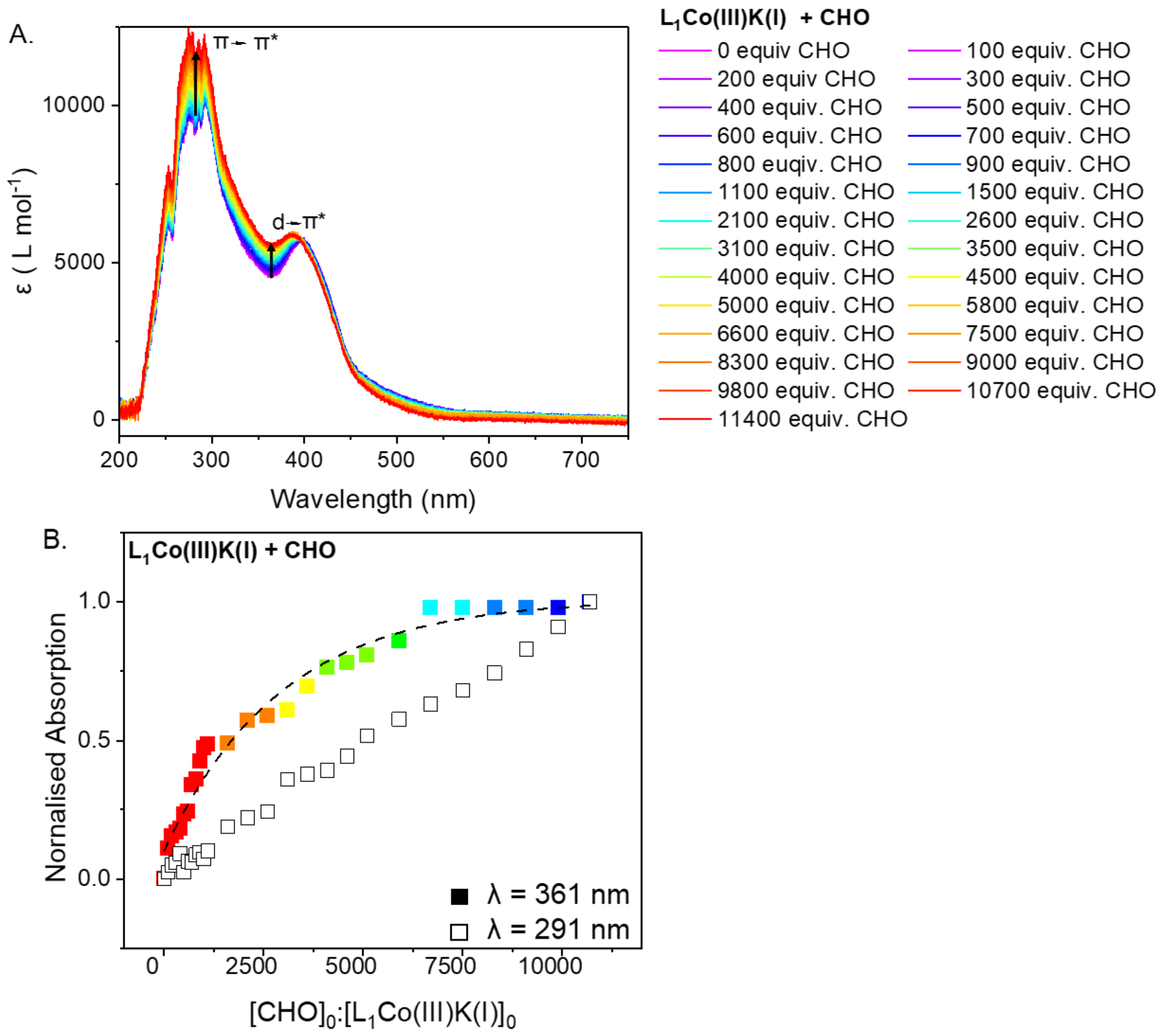

Fig. S17 A. UV-Vis spectroscopy data obtained from the titration of L_1Co(III)K(I) with increasing equivalents of CHO. B. Plot showing the normalized change in extinction coefficient at λ = 361 nm (d→π* transition, filled squares) and at λ = 291 nm (π→π* transition, unfilled squares), which do not shift uniformly, precluding the determination of a 1:1 association constant.

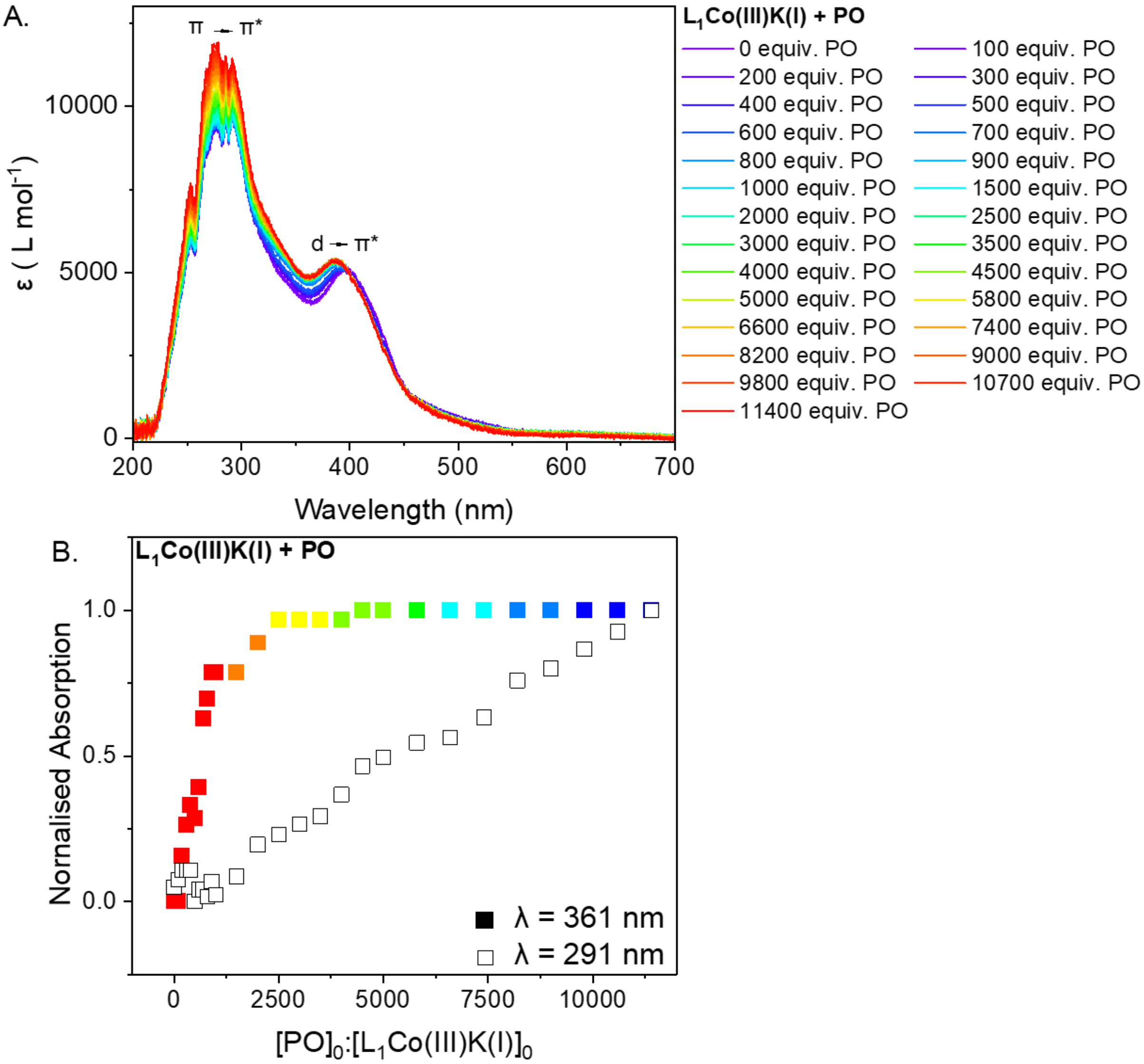

Fig. S18 A. UV-Vis spectra obtained from the titration of $L_1Co(III)K(I)$ with increasing equivalents of PO. B. Plot showing the normalized change in extinction coefficient at λ = 361 nm (d→π* transition, filled squares) and at λ = 291 nm (π→π* transition, unfilled squares), which do not shift uniformly, precluding the determination of a 1:1 association constant.

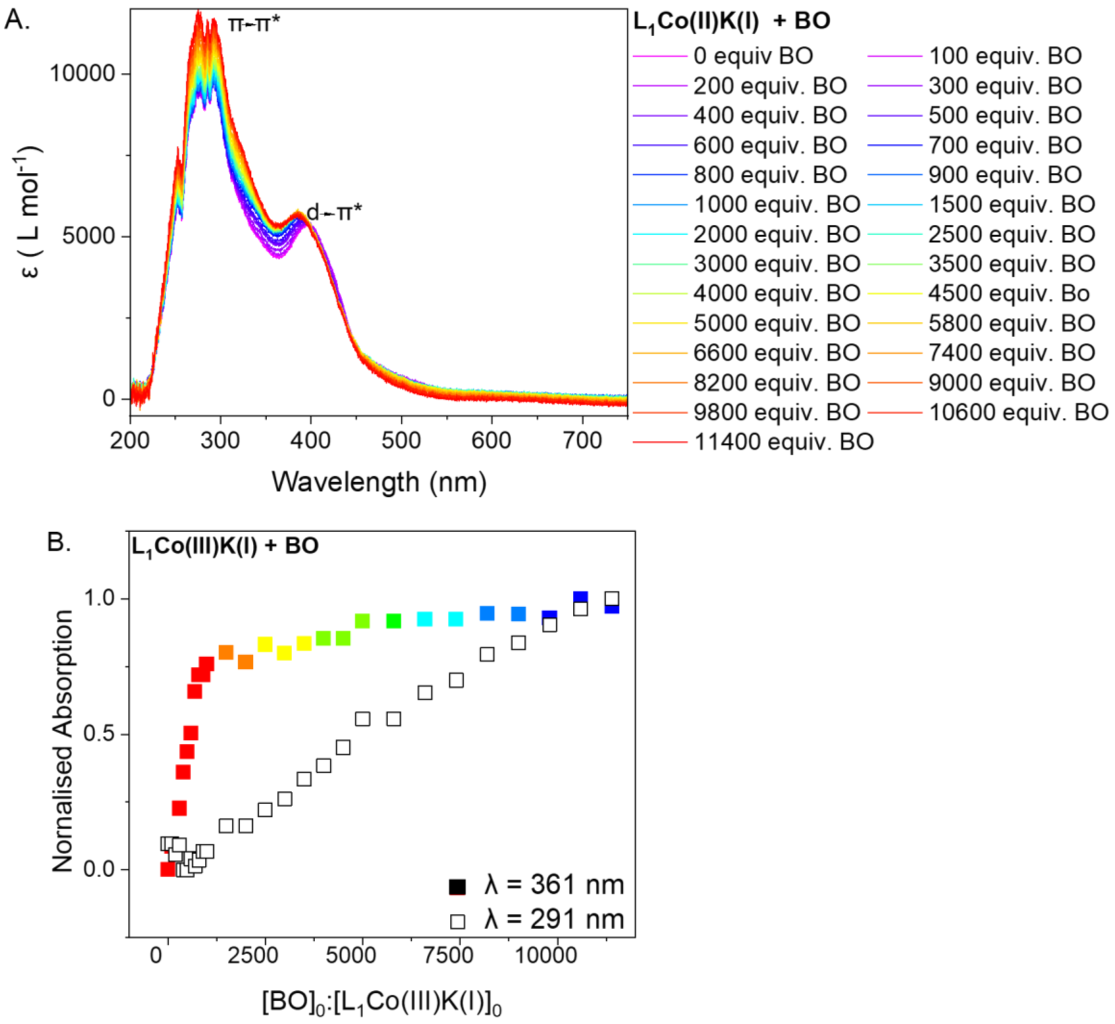

Fig. S19 A. UV-Vis spectra obtained from the titration of $L_1Co(III)K(I)$ with increasing equivalents of BO. B. Plot showing the normalized change in extinction coefficient at λ = 361 nm (d→π* transition, filled squares) and at λ = 291 nm (π→π* transition, unfilled squares), which do not shift uniformly, precluding the determination of a 1:1 association constant.

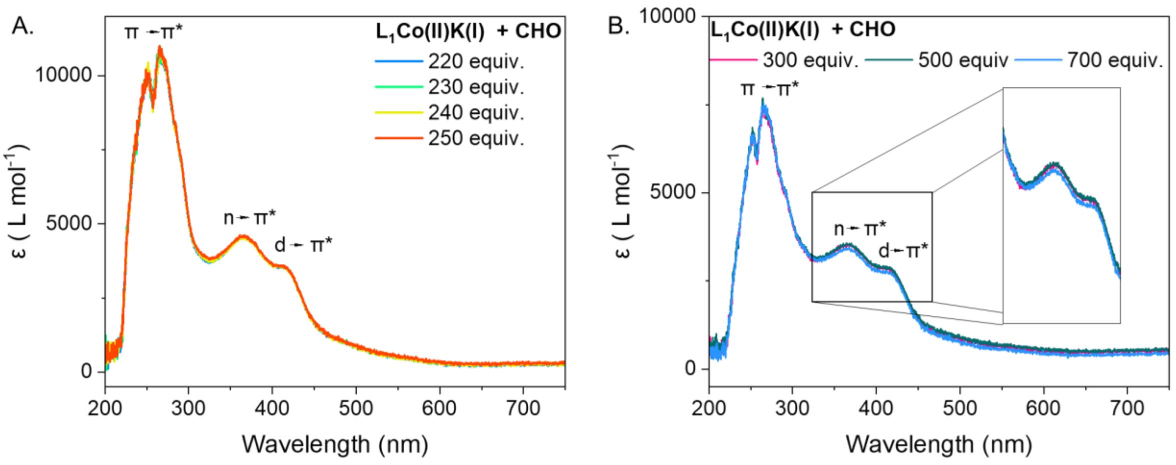

Fig. S20 UV-Vis spectra obtained from the titration of $L_1Co(II)K(I)$ with 200 – 700 equivalents CHO, demonstrating that no changes are observed upon the addition of 220 - 500 equivalents but the spectrum begins to change upon the addition of around 700 equivalents.

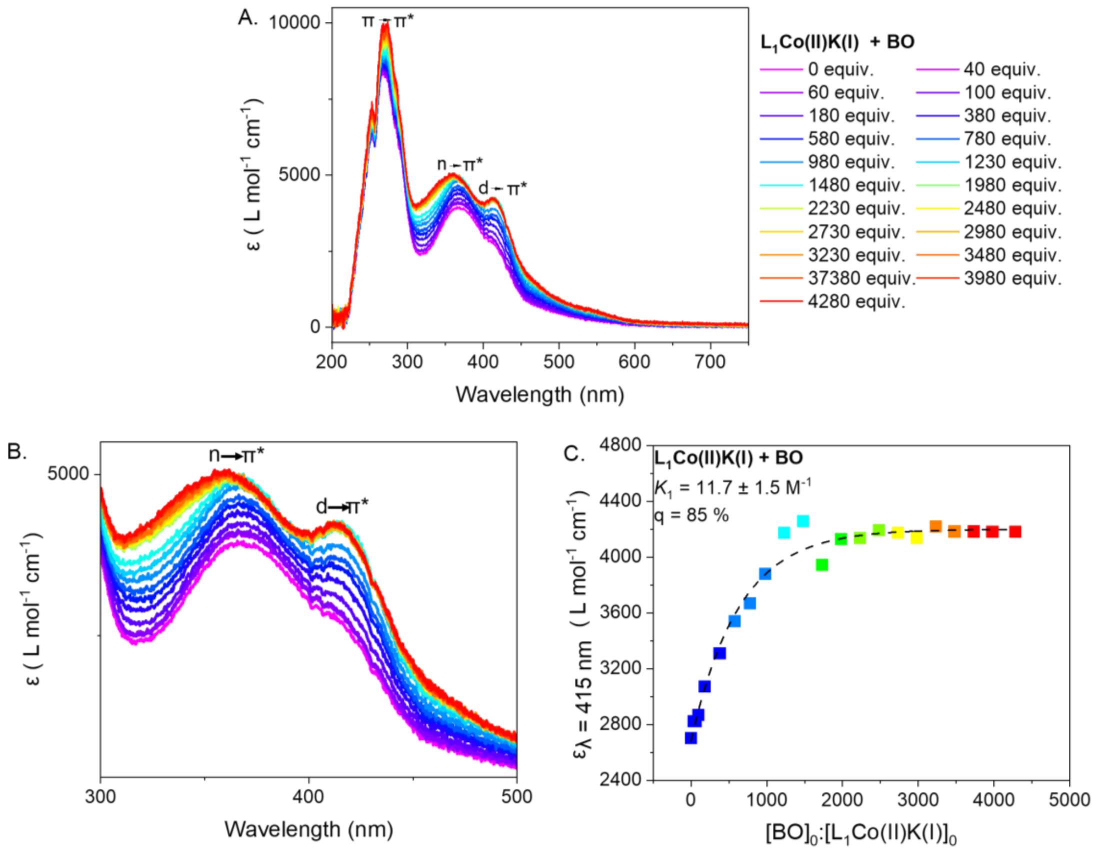

Fig. S21 A. UV-Vis spectra obtained from the titration of Co(II)K(I) with increasing equivalents of BO. Increasing equivalents of epoxide are represented by changing colours from purple to blue to yellow to orange and red. B. Key region (300 – 500 nm) of the spectra shown in A. that were used to obtain the association constant K_1. C. Fitting of the UV-Vis data shown in A. K_1 was obtained using supramolecular.org/Bindfit/. The fit and all fitting parameters are accessible through the link listed in Table S2.

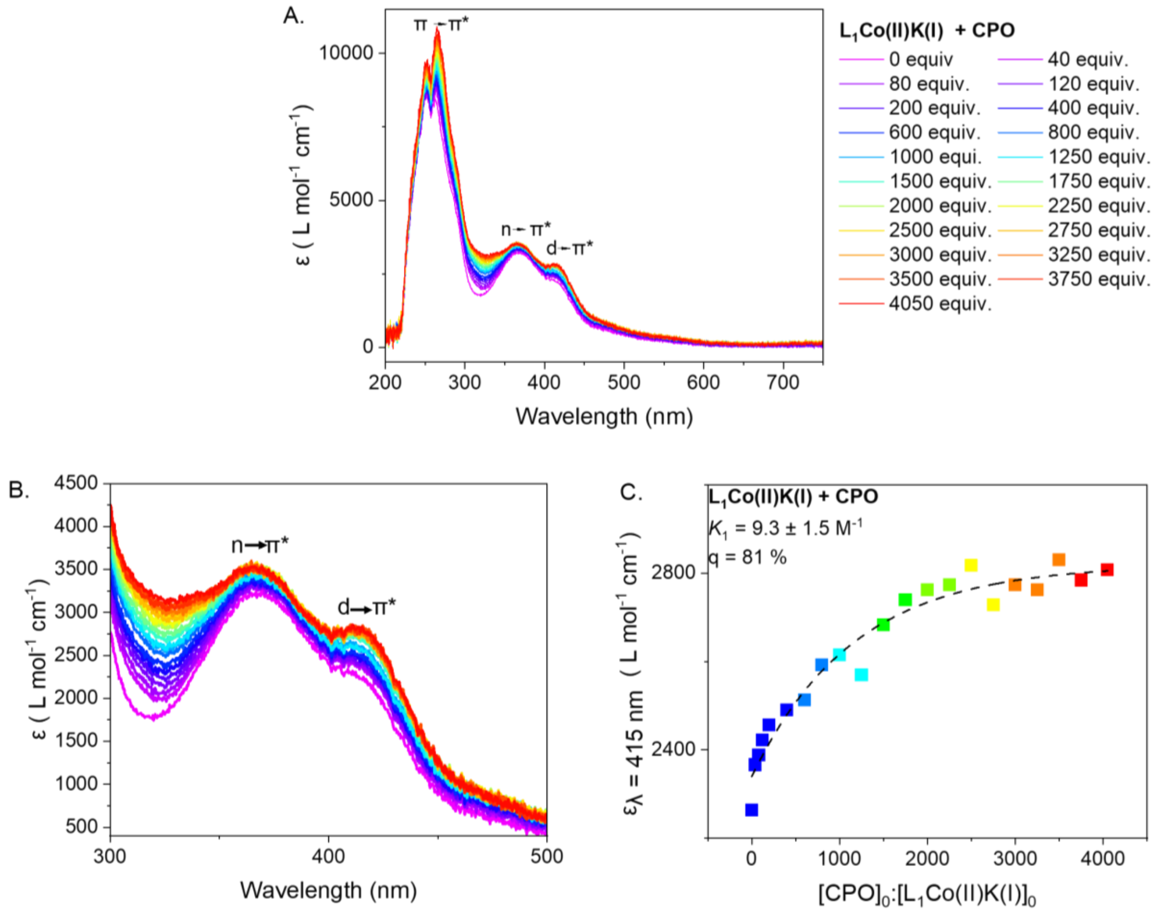

Fig. S22 A. UV-Vis spectra obtained from the titration of L1Co(II)K(I) with increasing equivalents of CPO. Increasing equivalents of epoxide are represented by changing colours from purple to blue to yellow to orange and red. B. Key region (300 – 500 nm) of the spectra shown in A. that were used to obtain the association constant K_1. C. Fitting of the UV-Vis data shown in A. K_1 was obtained using supramolecular.org/Bindfit/. The fit and all fitting parameters are accessible through the link listed in Table S2.

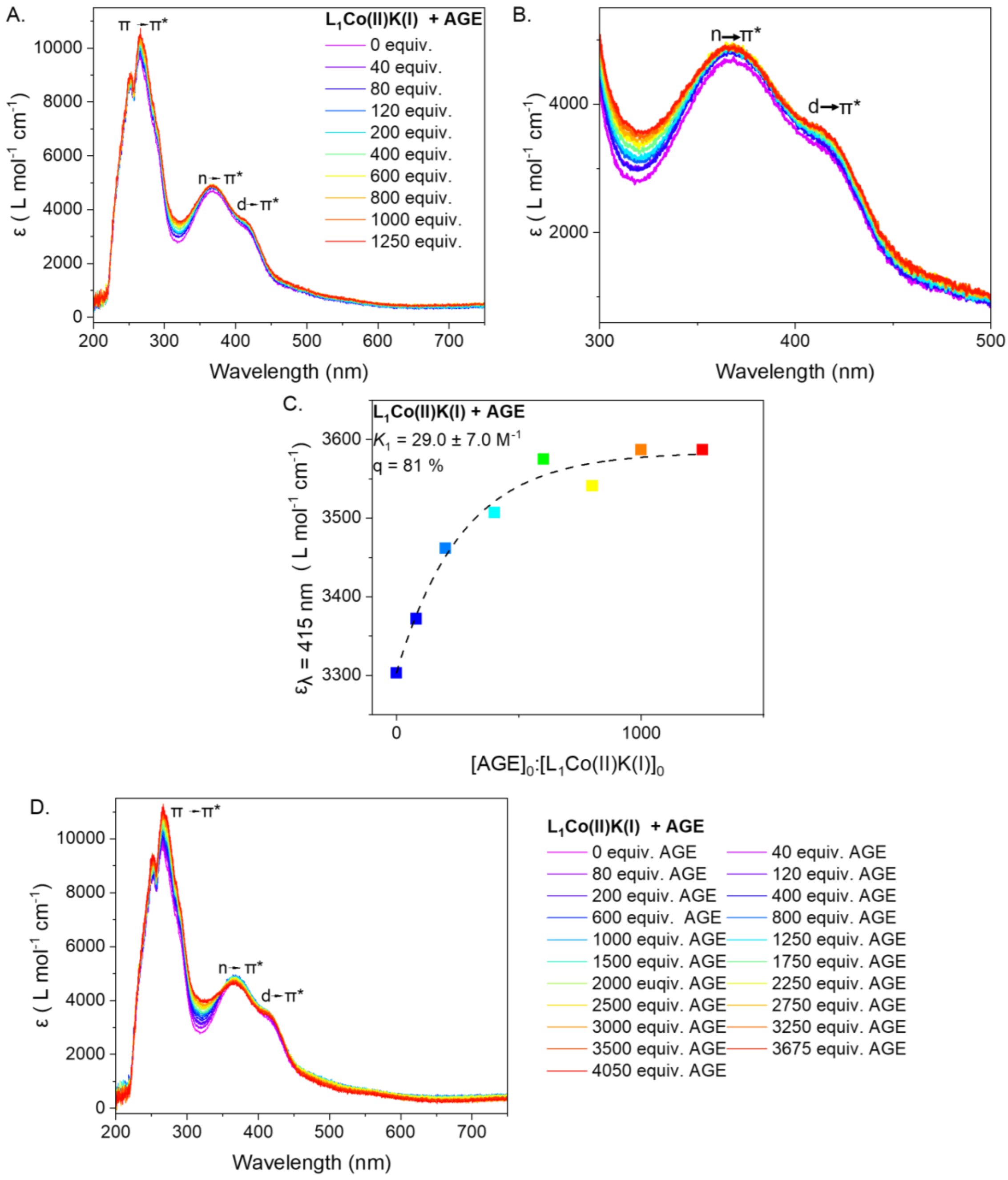

Fig. S23 A. UV-Vis spectra obtained from the titration of L_1Co(II)K(I) with increasing equivalents of AGE (0-1250 equiv.). Increasing equivalents of epoxide are represented by changing colours from purple to blue to yellow to orange and red. B. Key region (300–500 nm) of the spectra shown in A. that were used to obtain the association constant K_1. C. Fitting of the UV-Vis data shown in A. K_1 was obtained using supramolecular.org/Bindfit/. The fit and all fitting parameters are accessible through the link listed in Table S2. C. UV-Vis spectra obtained from the titration of Co(II)K(I) with increasing equivalents of AGE up to 4050 equivalents.

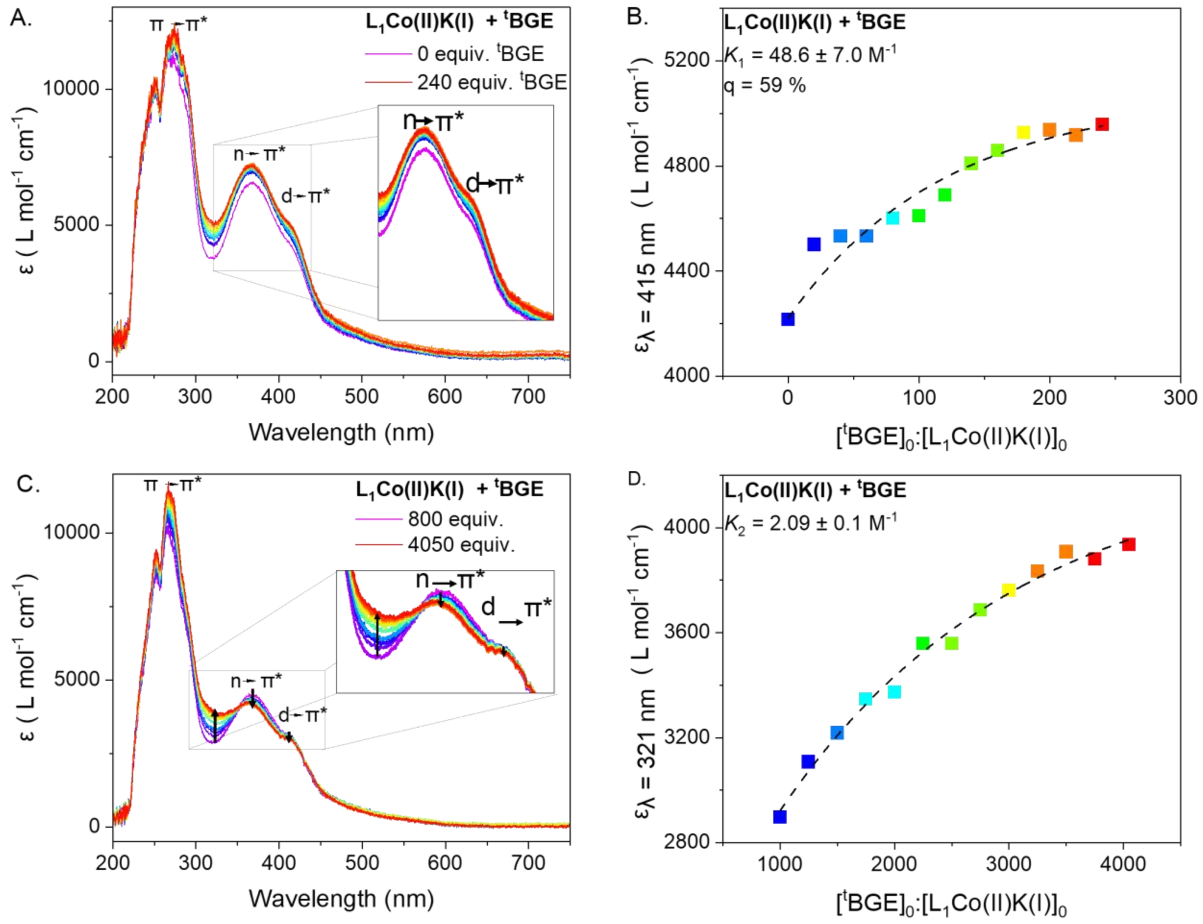

Fig. S24 UV-Vis spectra obtained by titrating L_1Co(II)K(I) with increasing concentrations of ^{t}BGE, demonstrating that A. saturation occurs at around 240 equivalents of [^{t}BGE]$_0$:[L_1Co(II)K(I)]$_0$. Titrations were conducted in 20 equiv. increments; hence, each different spectrum corresponds to an increased [^{t}BGE]$_0$:[L_1Co(II)K(I)]$_0$ of 20. B. Fitting of the UV-Vis spectroscopy data shown in A to obtain an association constant K_1 between 0 and 240 equiv. of ^{t}BGE. K_1 was obtained using bindfit.org, fitting parameters are listed in Table S2. C. UV-Vis spectra obtained by titrating L_1Co(II)K(I) with increasing concentrations of ^{t}BGE, demonstrating that a second saturation event occurs between 1000 – 4050 equivalents of [^{t}BGE]$_0$:[L_1Co(II)K(I)]$_0$. Titrations were conducted in 125 equiv. increments, hence, each different spectrum corresponds to an increased [^{t}BGE]$_0$:[L_1Co(II)K(I)]$_0$ of 125. D. Fitting of the UV-Vis spectroscopy data shown in C to obtain an association constant K_2 which describes a second binding event between 1000 and 4050 equiv. of ^{t}BGE. K_2 was obtained using bindfit.org, fitting parameters are listed in Table S2.

Table S2 Links to the fits to the UV-Vis spectroscopy data, used to determined association constants, describing binding of epoxides to L_1Co(II)K(I) using supramolecular.org/Bindfit/.

Host	Guest	Binding constan t	K determined between XX-YY equiv. epoxide added	Link to fitted data
Co(II)K(I)	PO	K_1	0- 4050 equiv.	http://app.supramolecular.org/bindfit/view/1df14273-7003-4225-8353-d180ad9c59ac
Co(II)K(I)	BO	K_1	0-4280 equiv.	http://app.supramolecular.org/bindfit/view/de7e4538-3542-4f1a-8d7f-6bc0ab034a64
Co(II)K(I)	CHO	K_1	0-250 equiv.	http://app.supramolecular.org/bindfit/view/c17551f1-67b8-4a19-a783-a7d1df4d4657
Co(II)K(I)	CHO	K_2	700-4040 equiv.	http://app.supramolecular.org/bindfit/view/3dbb8528-2fe1-4a68-a6c5-7a244d504305
Co(II)K(I)	AGE	K_1	0-1250 equiv.	http://app.supramolecular.org/bindfit/view/878c3dab-80c9-43d2-8984-1be99adedb8d
Co(II)K(I)	CPO	K_1	0-4050 equiv.	http://app.supramolecular.org/bindfit/view/183446a8-ea88-46f0-bd29-4b9ca9ee4711
Co(II)K(I)	^{t}BGE	K_1	0-240 equiv.	http://app.supramolecular.org/bindfit/view/ded79671-7f19-4f23-847b-a7b3d8ac978f
Co(II)K(I)	^{t}BGE	K_2	1000-4050 equiv.	http://app.supramolecular.org/bindfit/view/5b76de8f-5e94-475c-8c88-bfc303956e94

Calculation of q for UV-vis studies

The percentage of complex bound (denoted as q), was calculated from the fit obtained assuming a 1:1 binding, using bindfit (Table S2). From the so obtained data, the concentration of the epoxide-bound catalyst ([HE]) was determined from:

$[H] = [H]_0 – [HE]$ eq. S1

$[E] = [E]_0 – [HE]$ eq. S2

where $[H]_0$ and $[E]_0$ are the initial catalyst concentration and epoxide concentration.

Considering the association constant $K_{UV\text{-}vis}$ as:

$K = [HE] / [H][E]$ eq. S3

Substitution of eq. 1 and eq. 2 into eq. 3, gives:

$$[HE] = \frac{1}{2}\left\{\left([E]_0 + [H]_0 + \frac{1}{K}\right) - \sqrt{\left([E]_0 + [H]_0 + \frac{1}{K}\right)^2 - 4[[H]_0[E]_0}\right\} \quad \text{eq. S4}$$

From eq. S4 the concentration of epoxide bound catalyst was concentrated and q reported as the percentage [HE] of $[H]_0$.

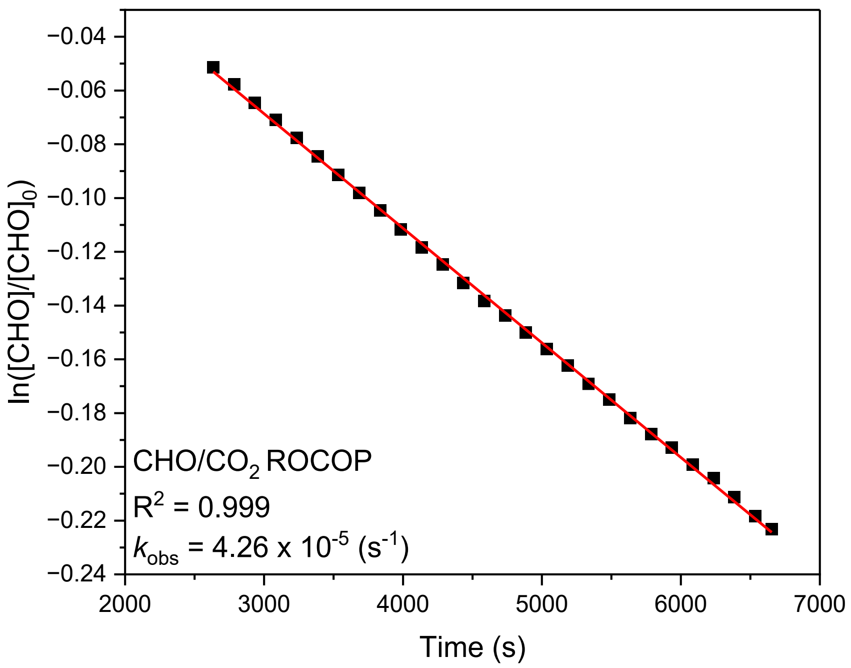

Fig. S25 Example plot of a semi-logarithmic plot of ln([epoxide]/[epoxide]$_0$) vs time, where k_{obs} is the slope of the plot, [cat]:[1,2-*trans* cyclohexane diol]:[epoxide] = 1:20:4000 (neat epoxide, 6 mL, 20 bar CO_2 pressure, 50 °C).

Table S3 Polymerization data for the polymerization of PO, CHO, BO, AGE, CPO and ᵗBGE. All polymerizations were conducted using [cat]:[1,2-trans cyclohexane diol]:[epoxide] = 1:20:4000 (neat epoxide, 6 mL, 20 bar CO_2 pressure, 50 °C). All values are reported as the average from n = 2 runs with the standard error of the mean.

#	Epoxide (Conc. / M)	t / h	Select. CO_2 / %[a]	Select. Poly (carbonate) / %[b]	Activity TOF / h^{-1} [c]	k_{obs} / x 10^{-7} s^{-1} [d]	k_p / x 10^{-3} s^{-1} M^{-1} [e]	Poly(carbonate) molar mass M_n [$Đ$] / g mol^{-1} [f]
1[1]	PO (14.29)	4	>99	>99 ± 0.4	328 ± 8	398 ± 0.7	11.2 ± 0.002	8000 [1.03]
2	CHO (9.89)	3	> 99	>99 ± 0	445 ± 24	416 ± 7	16.7 ± 0.26	4800 [1.12]
3	AGE (8.49)	6	> 99	94 ± 0.4	221 ± 5	175 ± 4	8.10 ± 0.2	8200 [1.10]
4	BO (11.49)	6	> 99	89 ± 0.02	255 ± 5	228 ± 1	7.99 ± 0.0005	9000 [1.04]
5	CPO (11.46)	7	>99	>99	153 ± 2	130 ± 0.002	4.59 ± 0.05	6800 [1.06]
	ᵗBGE (7.04)	6	>99	99 ± 1.1	229 ±14	196 ± 6.9	9.79 ± 0.35	8700 [1.09]

[a]CO_2 uptake was calculated from the ^1H NMR spectrum by dividing the sum of integrals for polycarbonate and cyclic carbonate against the sum of integrals for polycarbonate, cyclic carbonate, and polyether. [b]Poly(carbonate) selectivity was determined ^1H NMR spectrum by dividing the sum of integrals for polycarbonate against the sum of integrals for polycarbonate, cyclic carbonate, and polyether.[c] Turnover frequency (TOF) was calculated by dividing the turnover number (TON) against time, where TON was determined by dividing the moles of epoxide consumed determined by comparison of the sum of integrals by ^1H NMR spectroscopy of poly(carbonate), cyclic carbonate and poly(ether) against mesitylene (0.25 mol %) as an internal standard. [d]k_{obs} determined as the gradient of the plot of ln[epoxide]$_t$/[epoxide]$_0$ vs time. [e] k_p was determined by dividing k_{obs} by [catalyst] according to the rate law deduced previously at 20 bar.[1] [f]Determined by GPC in THF using narrow dispersity polystyrene standards. Representative values are shown for one repeat.

Density Functional Theory Calculations

Computational Methods

The Viking cluster, which is a high performance computing facility provided by the University of York, was used to perform DFT calculations. We are grateful for computational support from the University of York, IT Services and the Research IT team.

DFT calculations were run using Gaussian 16 (Revision C.01).[9] The reaction free energies were calculated using the long-range corrected hybrid exchange-correlation ωB97X-D functional,[10, 11] which also includes D2 dispersion corrections as described by Grimme.[12]

The split valence 6-31+g(d,p) basis sets were used for carbon and hydrogen. This lower basis set was chosen as these elements do not bind directly to either catalytic metal center, but extra diffuse functions were added to capture more mid- and long-range interactions, for instance with growing polymer chains. The triple-z 6-311+g(d) basis set was used for potassium and all heteroatoms. Cobalt centers were described with the Stuttgart SDD ECP and associated basis sets.

Geometry optimisation calculations were performed without symmetry constraints and using an "ultrafine" grid for numerical integration. All structures are optimized using the self-consistent reaction field (SCRF) approach with conductor-like polarisable continuum model (CPCM).[13] In polymerization reactions, the propylene oxide (PO) monomer also serves as the solvent but is not implemented as a standard solvent in Gaussian16. A CPCM model for PO was therefore implemented by using the built-in non-polar parameters of tetrahydrofuran, modified with the polar parameters of propylene oxide (ε=16, n^2=1.867) ([SCRF=(cpcm,solvent=tetrahydrofuran),read] and [eps=16/epsinf=1.867)].

Quasi-harmonic Gibbs free energies using Cramer and Truhlar's quasi-harmonic treatment of entropy[14] and Head-Gordon treatment of enthalpy[15] were extracted using the GoodVibes[16] Python library from the optimised structure files. To account for experimental conditions, a temperature of 323.15 K and concentration values as given experimentally were also applied for each system. For PO copolymerization: 14.2906336 mol L^{-1} (for neat PO), 0.003573 mol L^{-1} (for any metal complex species); for AGE copolymerization: 8.49833538 mol L^{-1} (for neat AGE), 0.002125 mol L^{-1} (for any metal complex species); for BO copolymerization: 11.4963251 mol L^{-1} (for neat BO), 0.002874 mol L^{-1} (for any metal complex species); for CPO copolymerization: 11.4598193 mol L^{-1} (for neat CPO), 0.002865 mol L^{-1} (for any metal complex species); for CHO copolymerization: 9.88353729 mol L^{-1} (for neat CHO), 0.002471 mol L^{-1} (for any metal complex species).

All intermediates and transition states were characterised by normal coordinate analysis revealing either precisely zero or one imaginary frequency, respectively. In the case of transition states, the imaginary frequency corresponds to the mode of the intended reaction step.

Conformational searches were performed with CREST v3.0.2[17], using the GFN2-xTB[18] method in the gas phase on the structures of Table S6. For epoxide ring-opening transition states, constraints were applied to the TS bond forming ($O_{carbonate}$–$C_{epoxide}$; 2.12122, 2.10358, 2.16157, 2.16165, 2.09138 Å for AGE, BO, CHO, CPO and PO, respectively) and breaking distances ($C_{epoxide}$–$O_{epoxide}$, 1.77579, 1.78931, 1.85363, 1.87173, 1.79417 Å for AGE, BO, CHO, CPO and PO, respectively), based on previously DFT-optimised TS structures. All other settings were left at their defaults (energy window 6 kcal mol^{-1}; RMSD threshold of 0.125 Å). DFT geometry optimisations of the resulting conformers and quasi-harmonic Gibbs free energies computations were then performed following the procedure outlined above.

Full coordinates for all DFT-optimised structures (including higher energy conformers), together with computed energies and vibrational frequency data, are available via the corresponding Gaussian 16 output files and calculation spreadsheet, and are stored alongside CREST output and ensemble files in the open-access digital repository: 10.6084/m9.figshare.28818275.

Modelling of the initiation step of the copolymerization of (*R*)-PO and CO_2 catalyzed by L_1Co(III)K(I)(OAc)$_2$

Table S4 Computed Free Gibbs Energies of intermediates and transition states for the first ring-opening of (R)-propylene by L_1Co(III)K(I)(OAc)$_2$ (see Fig. S26).

Structure	G (Hartree)[a]	ΔΔG (kcal mol^{-1})[a]	G (Hartree)[b]	ΔΔG (kcal mol^{-1})[b]
(*R*)-PO	-193.025528		-193.022173	
L_1Co(III)K(I)(OAc)$_2$	-2617.028824		-2617.039445	
Reference (L_1Co(III)K(I)(OAc)$_2$ + (*R*)-PO)	-2617.039445	0.0	-2810.061618	0.0
I_{Co}	-2810.0374	10.6	-2810.050731	6.8
$TS_{I\text{-}IICo}$	-2810.016939	23.5	-2810.029413	20.2
II_{Co}	-2810.064595	-6.4	-2810.075326	-8.6
I_K	-2810.051474	1.8	-2810.064164	-1.3
$TS_{I\text{-}IIK}$	-2809.999072	34.7	-2810.011595	31.4
II_K	-2810.020838	21.0	-2810.032329	18.4

[a]Calculations performed using rωb97xD functional, 6-31+g(d,p)/ 6-311+g(d)/SDD basis set and ECP, and modified cpcm solvation model (solvent=THF, ε=16, n^2=1.867). Default temperature = 298.15 K, concentration = 1.0 mol L^{-1}. [b]Goodvibes quasiharmonic correction applied at 323.15 K and concentration = 0.003573 mol L^{-1} (14.2906336 mol L^{-1} for [PO]).

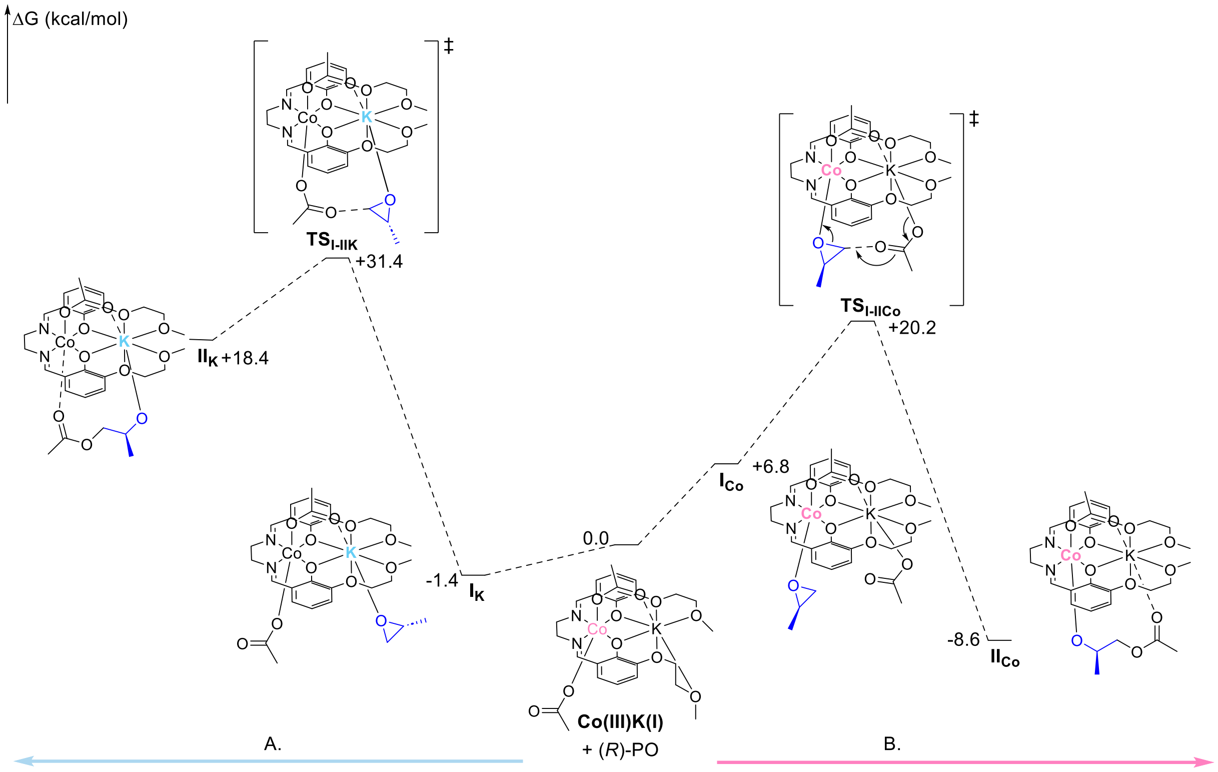

Fig. S26 Illustration of the Free Gibbs energy surface for the initiation step and first transition state of the copolymerization of (R)-PO with CO_2 using the Co(III)K(I) catalyst ($L_1Co(III)K(I)(OAc)_2$), where A. Epoxide coordination occurs at the K(I) center, and B. Epoxide coordination occurs at the Co(III) center.

Modelling of the propagation step of the copolymerization of PO and CO_2 catalyzed by L_1Co(III)K(I)

The starting point of the calculations is the metal complex L_1Co(III)K(I)(OAc)(O_2COR), in which one acetate ligand of 1 is replaced by one polycarbonate ligand (O_2COR, with R = $CH(CH_3)CH_2OCO_2CH_3$), modelling a growing polymer chain (see Fig. S27).

Table S5 Computed Free Gibbs Energies of intermediates and transition states for the ring-opening of (R)-propylene oxide by L_1Co(III)K(I)(OAc)(O_2COR) (see Fig S27).

Structure	G (Hartree)[a]	ΔΔG (kcal mol^{-1})[a]	G (Hartree)[b]	ΔΔG (kcal mol^{-1})[b]
(*R*)-PO	-193.025528		-193.022173	
L_1Co(III)K(I)(OAc)(O_2COR)	-3073.86685		-3073.878888	-3073.86685
Reference (L_1Co(III)K(I)(OAc)(O_2COR)+ (*R*)-PO)	-3266.892378	0.0	-3266.901061	0.0
I'_{Co}	-3266.881004	7.1	-3266.894023	4.4
TS'_{I-IICo}	-3266.850612	26.2	-3266.862095	24.4
II'_{Co}	-3266.889842	1.6	-3266.901737	-0.4
I'_{K}[c]	-3266.882665	6.1	-3266.896732	2.7
I'_{K}bis[c]	-3266.882136	6.4	-3266.894535	4.1
TS'_{I-IIK}	-3266.832609	37.5	-3266.843576	36.1
II'_{K}	-3266.852695	24.9	-3266.863146	23.8

[a]Calculations performed using rωb97xD functional, 6-31+g(d,p)/ 6-311+g(d)/SDD basis set and ECP, and modified cpcm solvation model (solvent=THF, ε=16, n^2=1.867). Default temperature = 298.15 K, concentration = 1.0 mol L^{-1}. [b]Goodvibes quasiharmonic correction applied at 323.15 K and concentration = 0.003573 mol L^{-1} (14.2906336 mol L^{-1} for [PO]). [c]In I'_K, (*R*)-PO is coordinated in equatorial position; in I'_Kbis, (*R*)-PO is coordinated in axial position.

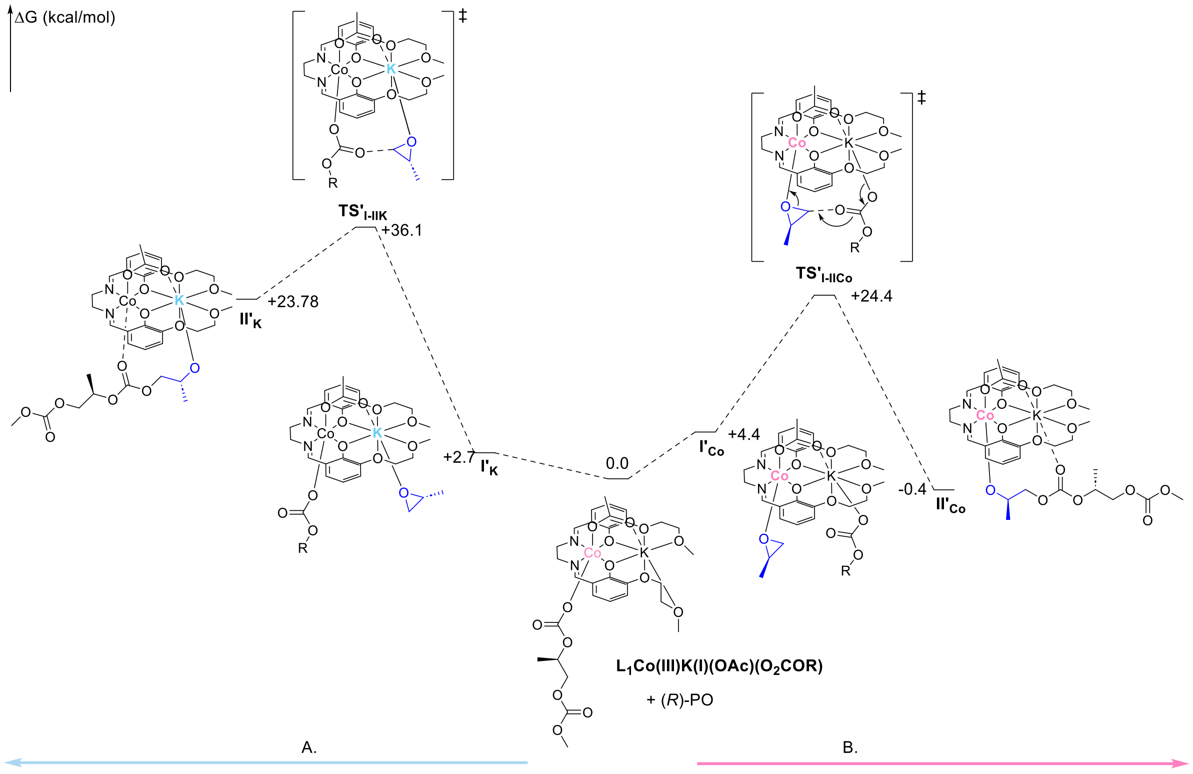

Fig. S27 Illustration of the Free Gibbs energy surface for a model propagation step of the copolymerization of (R)-PO with CO_2, and the (*R*)-PO ring-opening transition state, using the Co(III)K(I) catalyst (L_1Co(III)K(I)(OAc)(O_2COR)), where A. Epoxide coordination occurs at the K(I) center, and B. Epoxide coordination occurs at the Co(III) center.

Influence of the epoxide on the ring-opening activation barrier

Conformational searches were performed with CREST on transition states and related intermediates for the ring-opening of various epoxides by $L_1Co(III)K(I)(OAc)_2$, followed by the optimization by DFT of the conformers found, and the computation of their Free Gibbs Energies, as outlined above (see list of conformers and energies in digital repository). The activation barrier ($\Delta\Delta G^{\ddagger}_{epoxide}$) is taken as the difference in Free Gibbs Energies (in kcal/mol) between the intermediate with the lowest Free Gibbs Energy ($I_{K\text{-}epoxide}$) and the ring-opening transition state with the lowest Free Gibbs Energy ($TS_{I\text{-}IICo\text{-}epoxide}$).

Table S6 Computed Free Gibbs Energies of intermediates and transition states for the first ring-opening of epoxides by $L_1Co(III)K(I)(OAc)_2$ (see Fig S28).

Epoxide	Structure	G (Hartree)[a]	ΔΔG (kcal mol^{-1})[a]	G (Hartree)[b]	ΔΔG (kcal mol^{-1})[b]
CHO	CHO	-309.679553		-309.677053	
	$L_1Co(III)K(I)(OAc)_2$"	-2617.028824		-2617.039823	
	Reference $_{CHO}$	-2926.708377	0.0	-2926.716898	0.0
	$I_{K\text{-}CHO}$	-2926.709378	-0.6	-2926.72141	-2.8
	$I_{Co\text{-}CHO}$[c]	-2926.695132	8.3	-2926.70799	5.6
	$TS_{I\text{-}IICo\text{-}CHO}$	-2926.677564	19.3	-2926.689981	16.9
	Activation Barrier, $\Delta\Delta G^{\ddagger}_{CHO}$				+19.72
(*R*)-PO	(*R*)-PO"	-193.025528		-193.022196	
	$L_1Co(III)K(I)(OAc)_2$"	-2617.028824		-2617.039445	
	Reference $_{PO}$	-2810.054374	0.0	-2810.061641	0.0
	$I''_{K\text{-}PO}$	-2810.051463	1.8	-2810.064149	-1.6
	$I_{Co\text{-}PO}$[c]	-2810.0374	10.6	-2810.050731	6.8
	$TS''_{I\text{-}IICo\text{-}PO}$	-2810.018026	22.8	-2810.029413	20.2
	Activation Barrier, $\Delta\Delta G^{\ddagger}_{PO}$				+21.81
(*R*)-AGE	(*R*)-AGE	-345.573485		-345.571815	
	$L_1Co(III)K(I)(OAc)_2$"	-2617.028824		-2617.039977	
	Reference $_{AGE}$	-2962.602309	0.0	-2962.611792	0.0
	$I_{K\text{-}AGE}$	-2962.599977	0.8	-2962.614048	-1.4
	$I_{Co\text{-}AGE}$[c]	-2962.584787	10.2	-2962.597833	7.7
	$TS_{I\text{-}IICo\text{-}AGE}$	-2962.567170	22.1	-2962.578974	20.6
	Activation Barrier, $\Delta\Delta G^{\ddagger}_{AGE}$				+22.01
(*R*)-BO	(*R*)-BO	-232.304557		-232.302010	
	$L_1Co(III)K(I)(OAc)_2$"	-2617.028824		-2617.039668	
	Reference $_{BO}$	-2849.333381	0.0	-2849.341678	0.0
	$I_{K\text{-}BO}$	-2849.331433	1.2	-2849.331433	-2.0
	$I_{Co\text{-}BO}$[c]	-2849.316479	10.6	-2849.330034	7.3
	$TS_{I\text{-}IICo\text{-}BO}$	-2849.297704	22.4	-2849.309943	19.9
	Activation Barrier $\Delta\Delta G^{\ddagger}_{BO}$				+21.88
CPO	CPO	-270.396736		-270.393838	
	$L_1Co(III)K(I)(OAc)_2$"	-2617.028824		-2617.039671	

	Reference $_{CPO}$	-2887.425594	0.0	-2887.433509	0.0
	$I_{K\text{-}CPO}$	-2887.424473	0.7	-2887.437208	-2.3
	$I_{Co\text{-}CPO}$[c]	-2887.412626	8.17	-2887.425501	5.0
	$TS_{I\text{-}IICo\text{-}CPO}$	-2887.388785	23.1	-2887.401325	20.2
	Activation Barrier, $\Delta\Delta G^{\ddagger}_{CPO}$				+22.52

[a]Calculations performed using rωb97xD functional, 6-31+g(d,p)/ 6-311+g(d)/SDD basis set and ECP, and modified cpcm solvation model (solvent=THF, ε=16, n^2=1.867). Default temperature = 298.15 K, concentration = 1.0 mol L^{-1}. [b]Goodvibes quasiharmonic correction applied at 323.15 K and concentrations: for PO copolymerization: 14.2906336 mol L^{-1} (for neat PO), 0.003573 mol L^{-1} (for any metal complex species); for AGE copolymerization: 8.49833538 mol L^{-1} (for neat AGE), 0.002125 mol L^{-1} (for any metal complex species); for BO copolymerization: 11.4963251 mol L^{-1} (for neat BO), 0.002874 mol L^{-1} (for any metal complex species); for CPO copolymerization: 11.4598193 mol L^{-1} (for neat CPO), 0.002865 mol L^{-1} (for any metal complex species); for CHO copolymerization: 9.88353729 mol L^{-1} (for neat CHO), 0.002471 mol L^{-1} (for any metal complex species). [c]To minimize computational cost, no conformational search was performed for these structures. " denotes structures which have been subjected to further conformational search from those of Table S4.

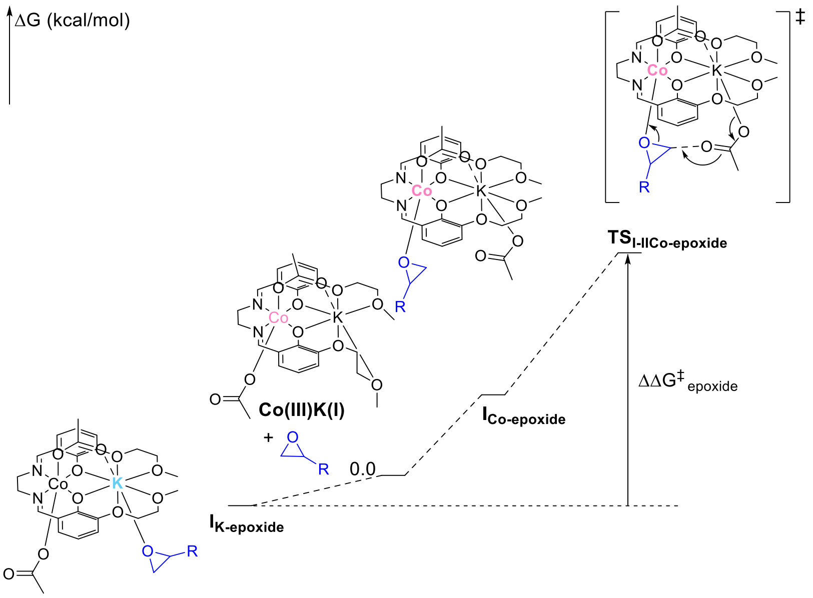

Fig. S28 Illustration of the Gibbs Free energy surface for the initiation step and first transition state of the copolymerization of epoxide with CO_2 using the Co(III)K(I) catalyst

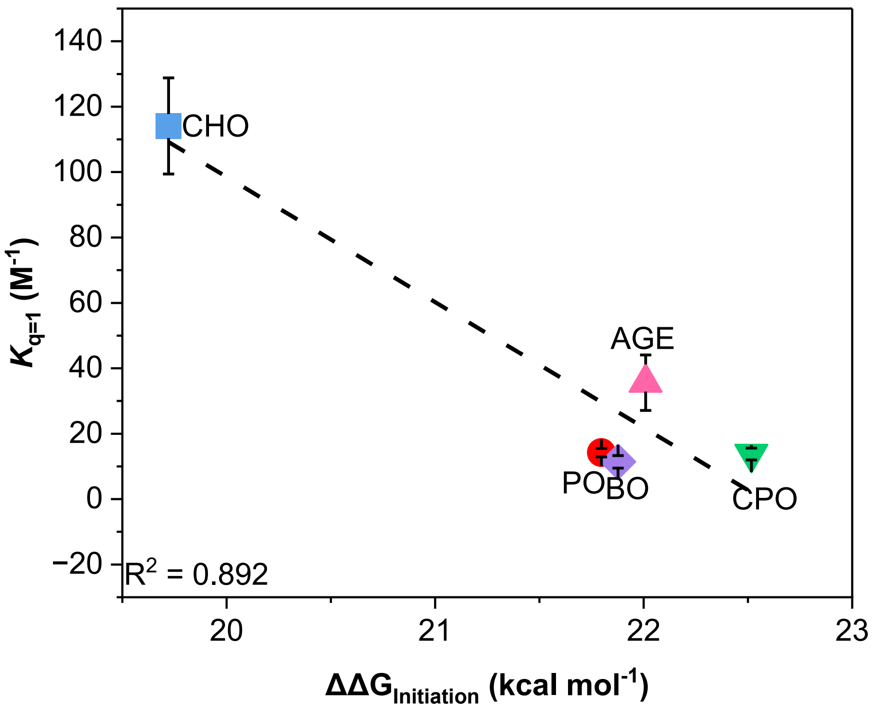

Fig. S29 Correlation between the experimentally determined binding constant $K_{q=1}$ and the calculated initiation barrier to epoxide ring opening as illustrated in Fig. S28.

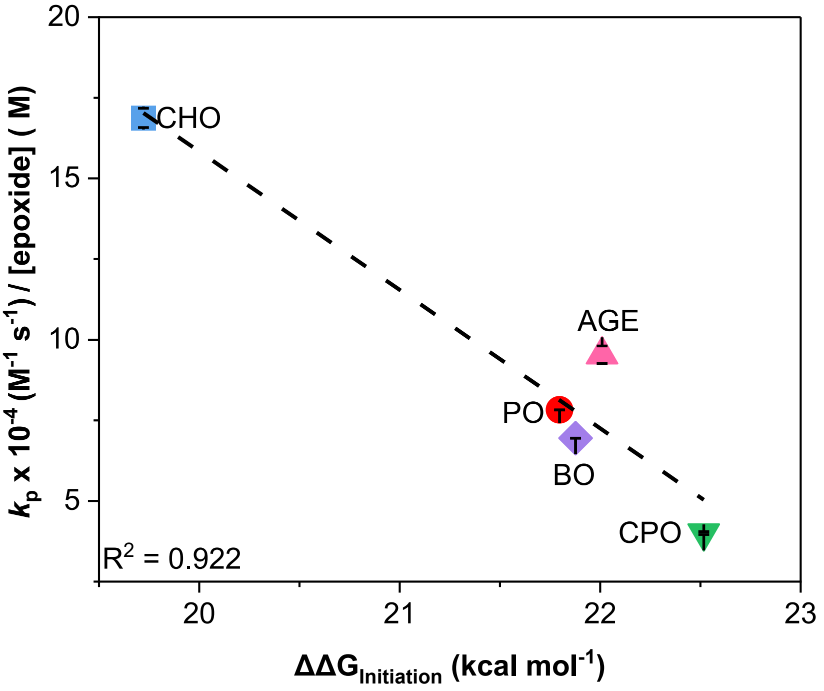

Fig. S30 Correlation between the experimentally determined polymerisation rate constant (k_p), normalized to epoxide concentration, and the calculated initiation barrier to epoxide ring opening as illustrated in Fig. S28.

Preliminary Cyclic Voltammetry Studies on Epoxide Binding to $L_1Co(III)K(I)$

The following section details preliminary CV experiments, which aimed to establish whether electrochemical methods could be used in the future to measure epoxide binding to redox active M(III) catalyst. This data was included as it demonstrates that upon the addition of increasing equivalents of epoxides to $L_1Co(III)K(I)$, significant shifts in a reduction peak can be observed (Fig. S33). However, this data should be treated as preliminary as the assignment of the peak that was monitored remains unclear (Fig.S31).

To study whether the two unassigned peaks are coupled, we systematically varied the scan rate and monitored the peak separation between the two peaks. Upon a reduction in scan rate, the separation of the two peaks decreases, however, we the peak separation remains large (at around 70 mV) even at 6.24 mV s^{-1}. It therefore remains unclear whether the peaks are related (Fig. S32).

To ensure that the additional peaks do not correspond to the K(I) ion falling out under electrochemical conditions, we conducted control experiments with a monometallic $L_1Co(III)$ complex (Fig. S31C). The voltammogram of the monometallic Co(III) complex also displays an additional reduction peak at E_{red} = 1.53 V (vs Fc^+/Fc^0), upon the addition of excess KOAc salt to the solution, this peak disappears, and a second reduction at E_{red} = 1.46 V (vs Fc^+/Fc^0) appears, which is assigned as the dinuclear Co(III)K(I) complex. As an additional reduction peak is also present in the voltammogram of the monometallic Co(III) complex, it is therefore hypothesized that the additional peak observed for $L_1Co(III)K(I)$, does not correspond to the loss of K(I) ions. Based on these data, it remains unclear how to assign the reduction peak at E_{red} = -1.52 V (vs Fc^+/Fc^0) and an oxidation at E_{ox} = -0.35 V (vs Fc^+/Fc^0), and further detailed spectroelectrochemical studies would be necessary to obtain a clearer picture.

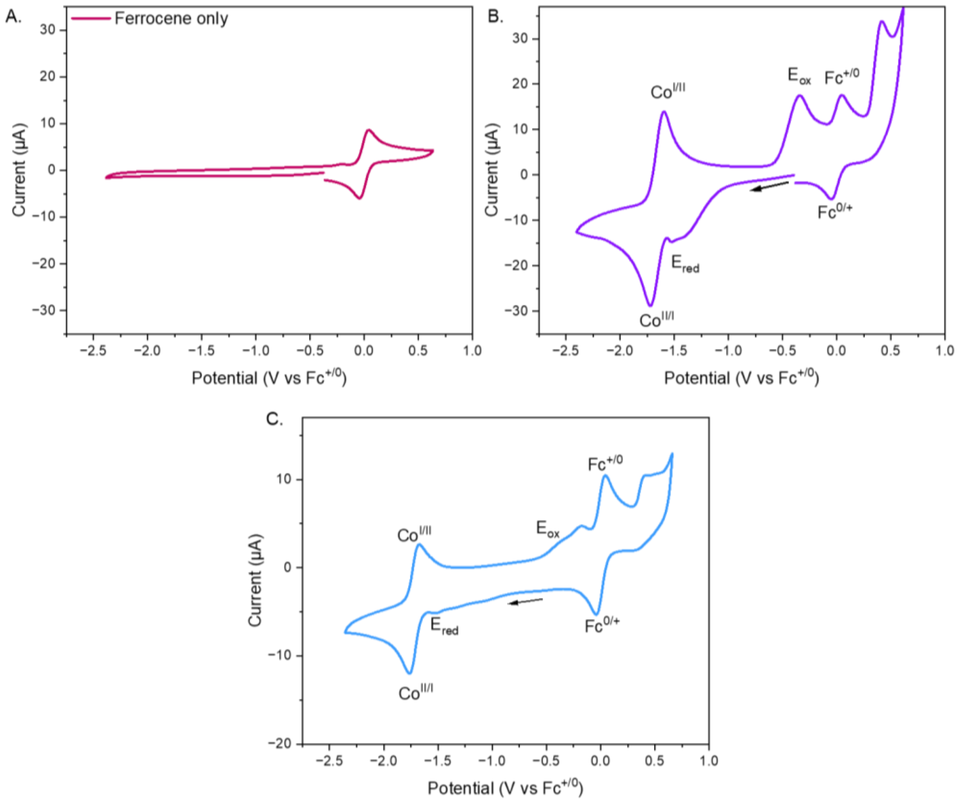

Fig. S31 A. CV of ferrocene, shown as background for B and C. B. CV of $L_1Co(III)K(I)$, C. CV of $L_1Co(III)$: All CVs were collected at approximately 1 mM analyte conditions, using 0.1 M $TBAPF_6$ in MeCN, scan rate = 0.1 V s^{-1}.

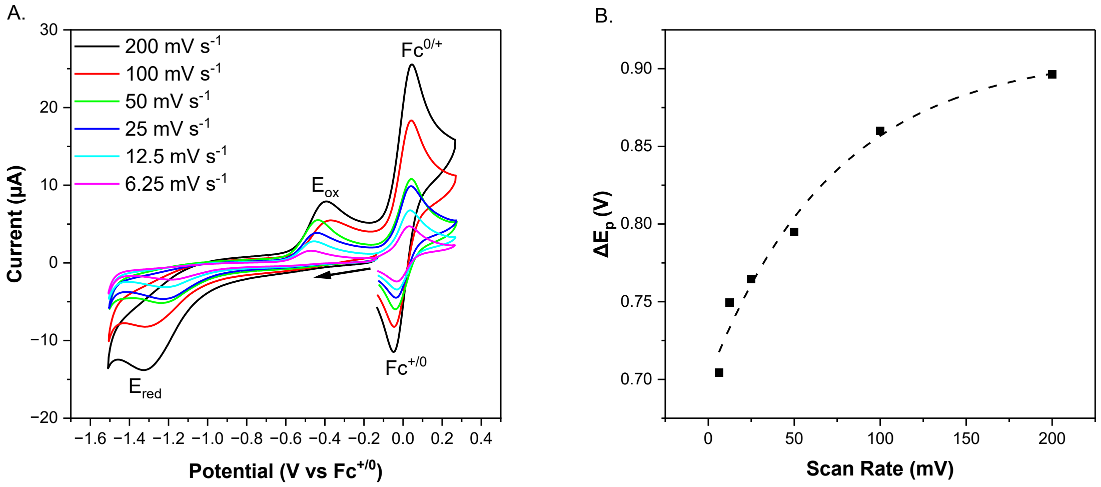

Fig. S32 Investigation of electrochemical behaviour of L_1Co(III)K(I) with scan rate. A. Cyclic Voltammograms showing the decrease in peak distance for the Co(III/II) redox event with decreasing scan rate. B: Plot of the peak distance with decreasing scan rate.

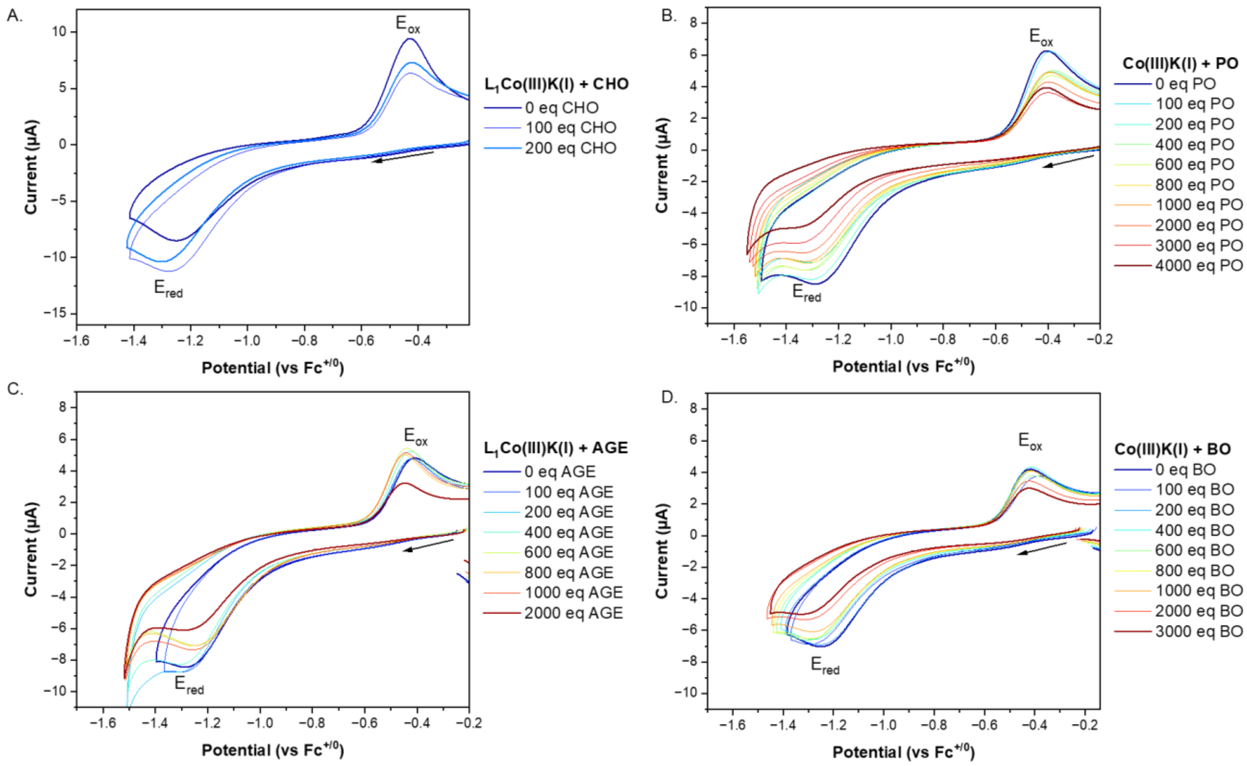

Fig. S33 Voltammograms of L_1Co(III)K(I) with increasing equivalents of epoxide: A. CHO, B. PO, C. AGE, D. BO.

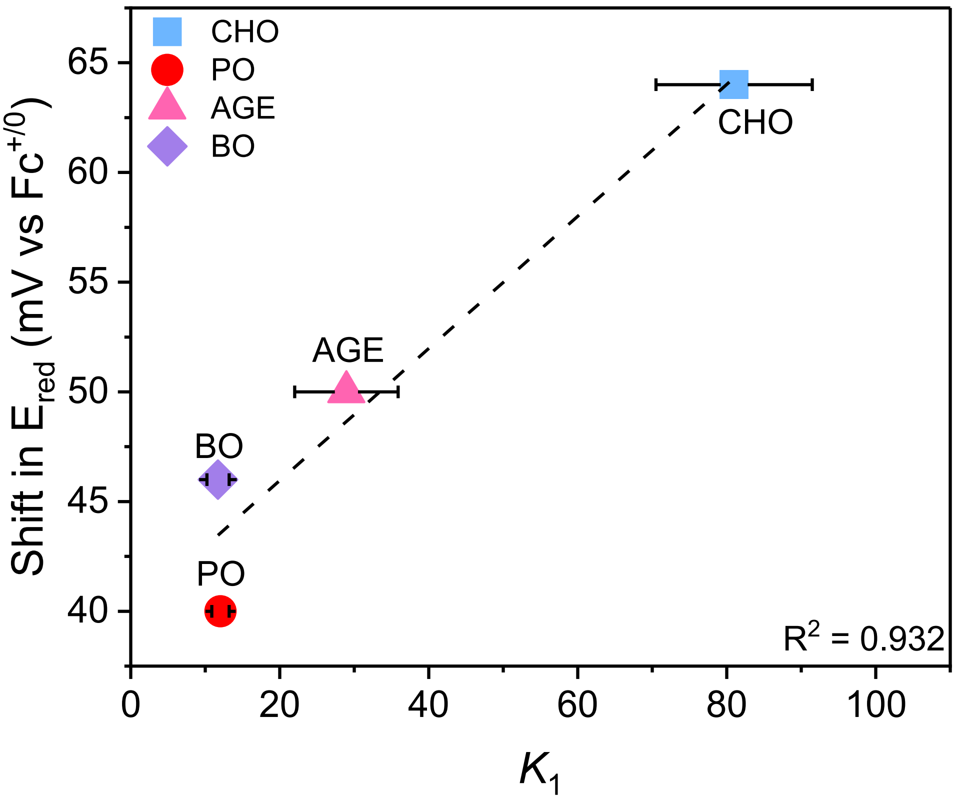

Fig. S34 Plot showing the shift in E_{red} upon epoxide addition compared to $L_1Co(III)K(I)$ in the absence of any epoxide vs the binding constant, K_1, determined by UV-Vis spectroscopy.

Preliminary Study of Epoxide Binding to $L_2Co(III)K(I)$

Synthesis and Characterization

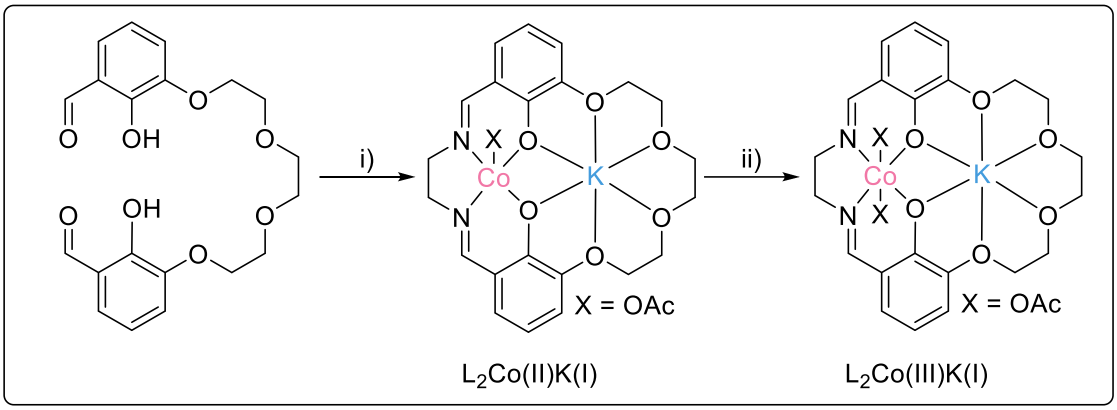

Fig. S35 Synthesis of $L_2Co(II)K(I)$ and $L_2Co(III)K(I)$, where i) Ethylenediamine, $Co(II)OAc_2$, KOAc, MeCN (99% conversion, 38 % isolated yield (220 mg)) ii) 2 equivalents AcOH, MeCN, air (20 % isolated yield (120 mg)).

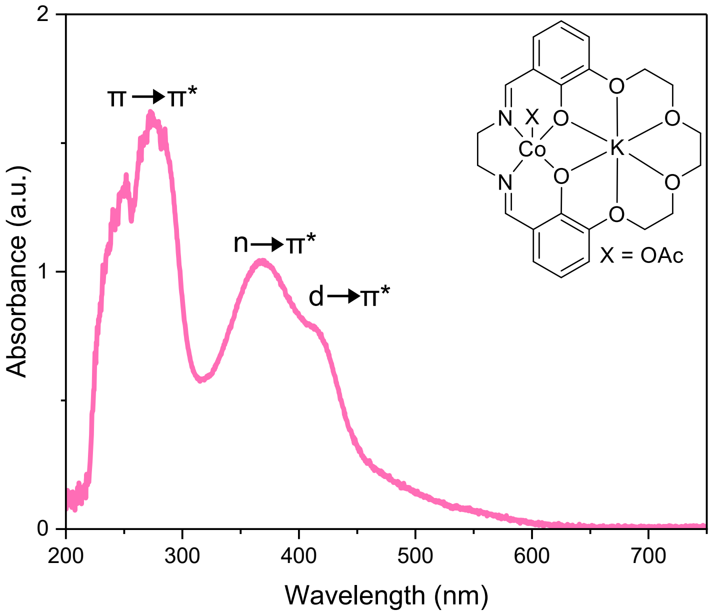

Fig. S36 UV-Vis spectrum of $L_2Co(II)K(I)$ (0.125 mM in MeCN).

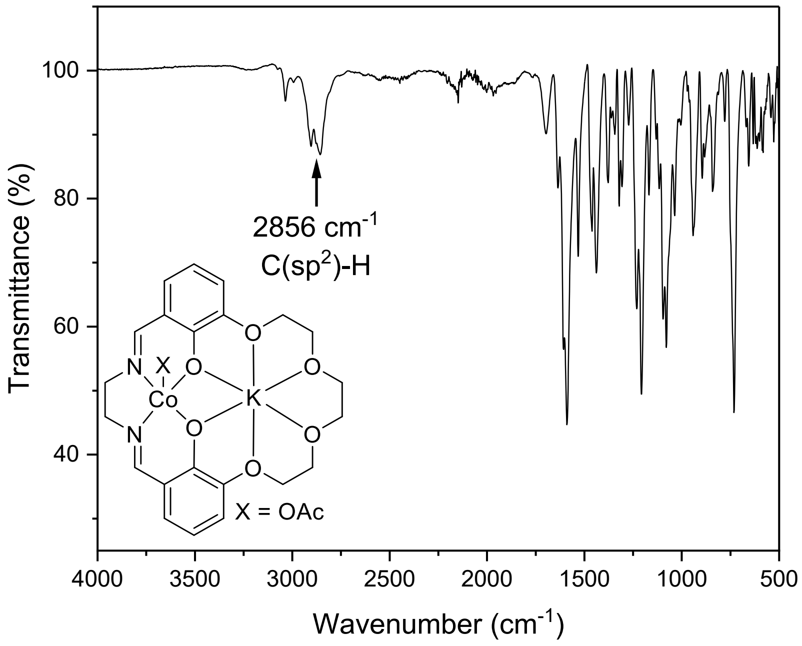

Fig. S37 IR spectrum of $L_2Co(II)K(I)$.

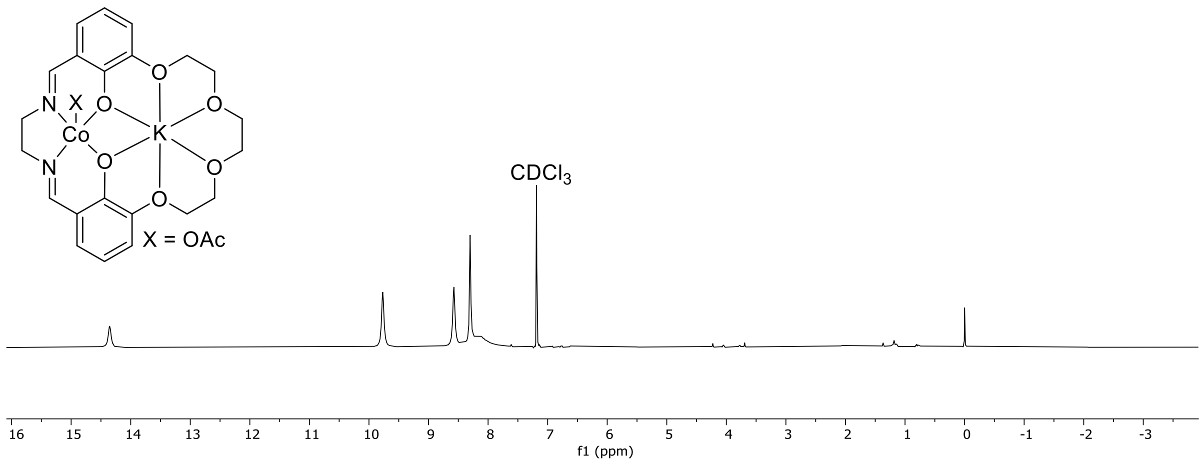

Fig. S38 ^1H NMR spectrum of $L_2Co(II)K(I)$ in $CDCl_3$.

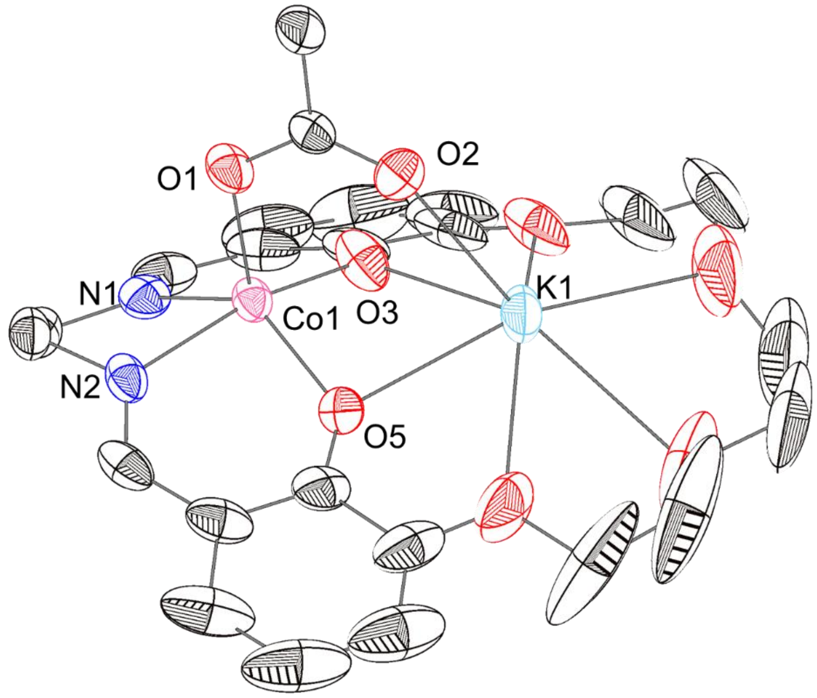

Fig. S39 Solid state structure of $L_2Co(II)K(I)$ obtained by single XRD. Thermal ellipsoids are shown at a probability of 50 %. Hydrogens are omitted for clarity. See Table S9-10 for details.

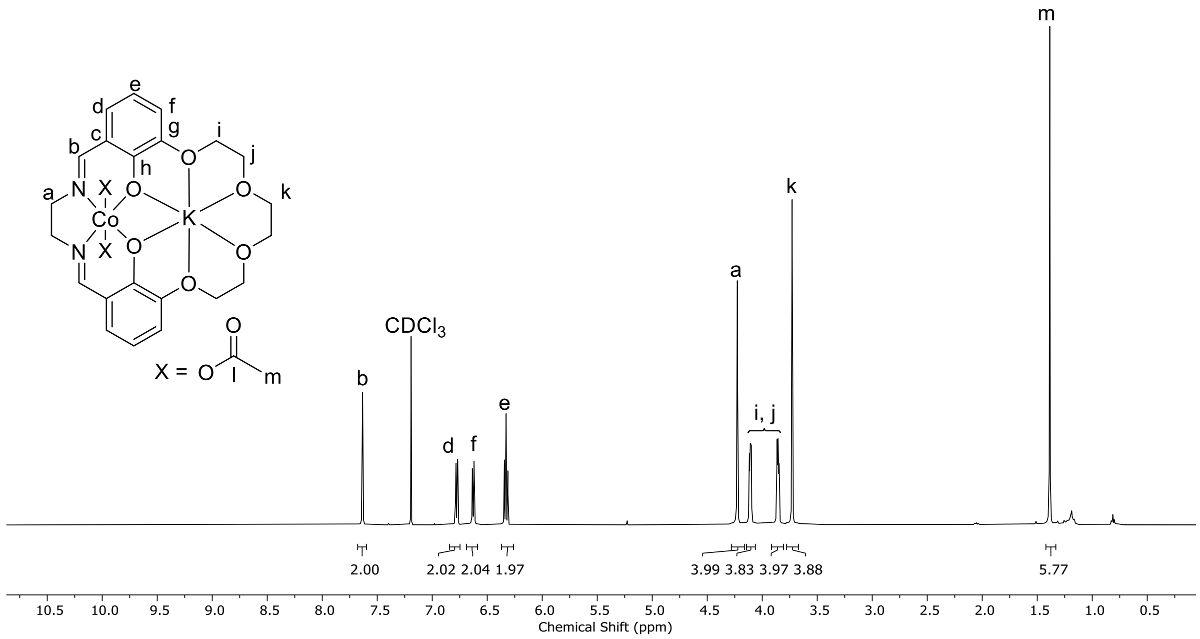

Fig. S40 ^{1}H NMR spectrum of $L_2Co(III)K(I)$ in $CDCl_3$.

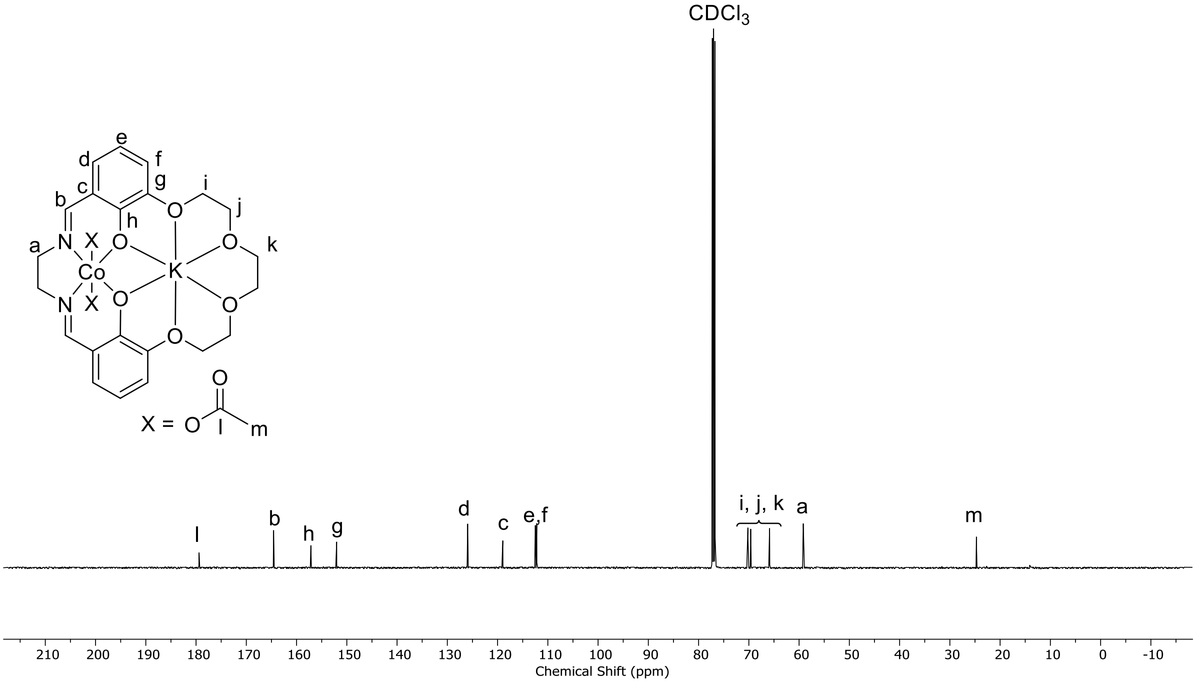

Fig. S41 $^{13}C\{^{1}H\}$ NMR spectrum of the previously reported ligand $L_2Co(III)K(I)$ in $CDCl_3$.

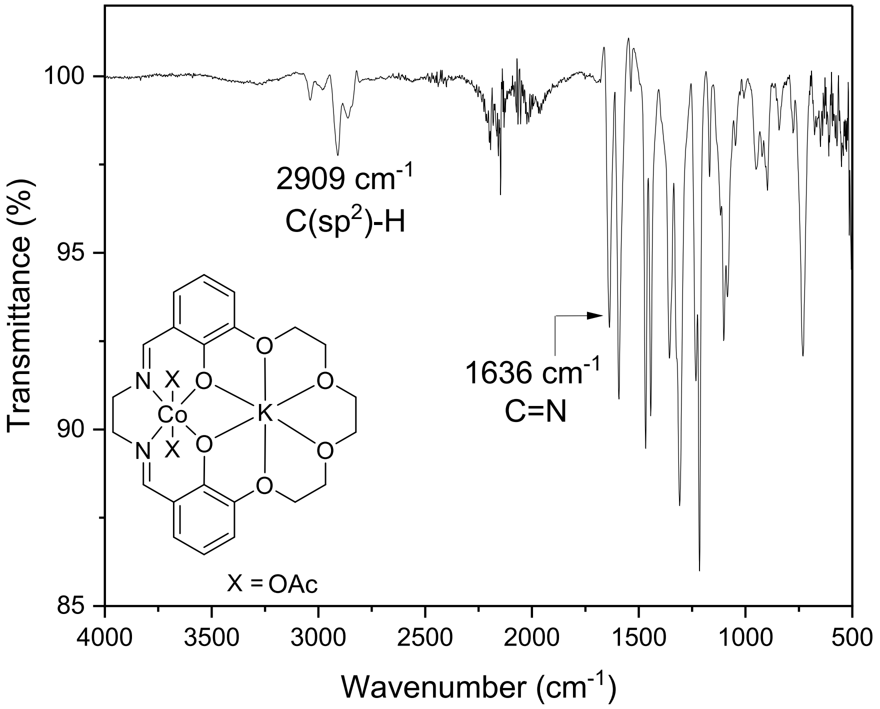

Fig. S42 IR spectrum of $L_2Co(III)K(I)$

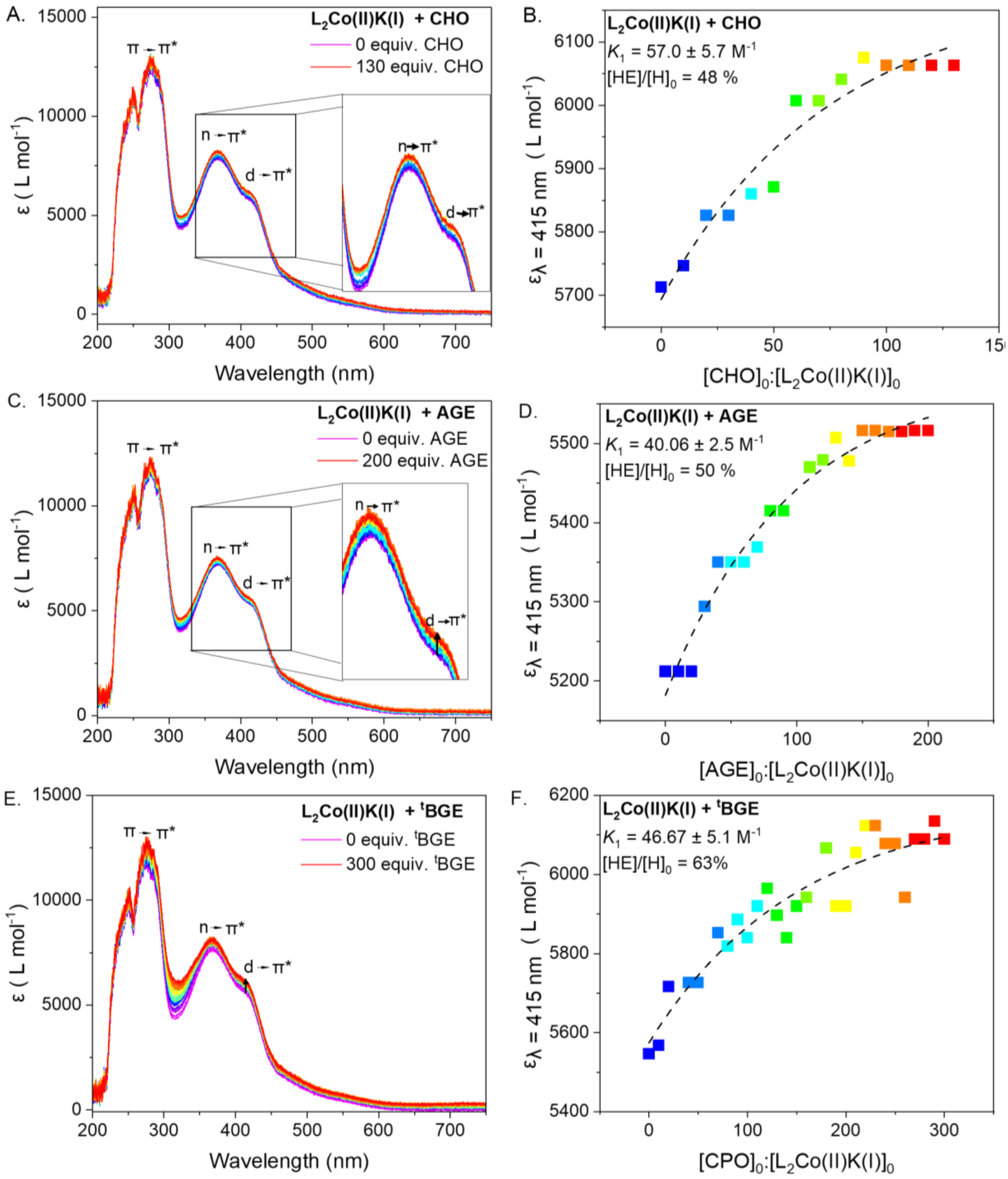

Fig. S43 UV-Vis spectra obtained by titrating $L_2Co(II)K(I)$ with increasing equivalents of A. CHO, C. AGE and E. ^tBGE. Increasing equivalents of epoxide are represented by changing colours from purple to blue to yellow to orange and red. Additions were performed in 10 equivalents increments. Plot showing the change in normalised absorbance of the peak 415 nm (d→π* transition, represented with filled squares) with increasing equivalents of B. CHO, D. AGE and F. ^tBGE. The association constant K_1 was obtained from the fit of the change in absorbance of the d→π* transition at 415 nm fort each epoxide using non-linear regression modelling accessed through the Bindfit calculator (supramolecular.org/bindfit/). The fit of the data is accessible through the link provided in Table S7.

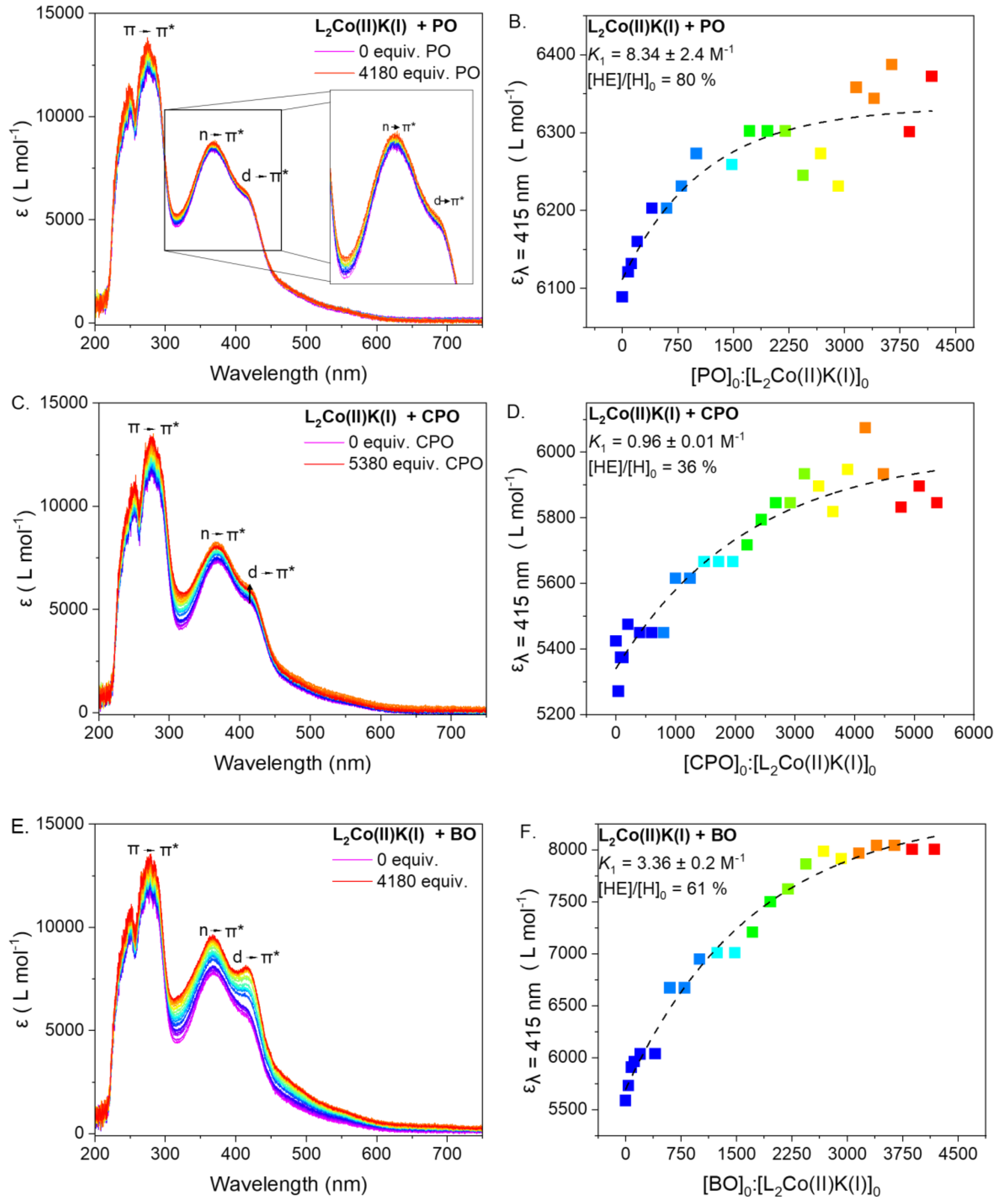

Fig. S44 UV-Vis spectra obtained by titrating $L_2Co(II)K(I)$ with increasing equivalents of A. PO, C. CPO and E. BO. Increasing equivalents of epoxide are represented by changing colours from purple to blue to yellow to orange and red. Additions were performed in 40 equivalents increments from 0-120 equivalents epoxide, in 200 equivalents increments between 200-1000 equivalents epoxide and in 240 equivalents increments from above 1000 equivalents of epoxide. Plot showing the change in normalised absorbance of the peak 415 nm (d→π* transition, represented with filled squares) with increasing equivalents of B. PO, D. CPO and F. BO. The association constant K_1 was obtained from the fit of the change in absorbance of the d→π* transition at 415 nm fort each epoxide using non-linear regression modelling accessed through the Bindfit calculator (supramolecular.org/bindfit/). The fit of the data is accessible through the link provided in Table S7.

Table S7 Links to the fits to the UV-Vis spectroscopy data, used to determined association constants, describing binding of epoxides to $L_2Co(II)K(I)$ using supramolecular.org/Bindfit/.

Host	Guest	K_1 (M^{-1})	q (%)	$K_{q=1}$ (M^{-1})	K determined between XX-YY equiv. epoxide added	Link to fitted data
Co(II)K(I)	PO	8.34 ± 2.4	79.7	10.5 ± 3.0	0-4180	http://app.supramolecular.org/bindfit/view/6ee954e6-cad1-4f14-a5e2-e7b1bd440005
Co(II)K(I)	BO	3.36 ± 0.2	61.3	5.47 ± 0.4	0-4180	http://app.supramolecular.org/bindfit/view/cfc20dcf-90a1-436c-a7bb-335a2266274b
Co(II)K(I)	CHO	57.02 ± 5.7	47.6	119.8 ± 12.0	0-130	http://app.supramolecular.org/bindfit/view/6a8cc6fd-98ba-4a8b-bd7e-5f0281ca4984
Co(II)K(I)	AGE	40.06 ± 0.1	49.4	81.2 ± 5.2	0-200	http://app.supramolecular.org/bindfit/view/f833253c-75a2-47a1-a9bd-83cc90c4cb76
Co(II)K(I)	CPO	0.96 ± 0.1	36. 3	2.6 ± 0.03	0-5380	http://app.supramolecular.org/bindfit/view/2c5ea7b8-dc95-4d481-8d78-2b4fe28c5016
Co(II)K(I)	^{t}BGE	46.7 ± 5.1	62.7	74.4 ± 8.13	0-300	http://app.supramolecular.org/bindfit/view/ac4d2dc6-599e-44f7-8df9-a952ff86aa9b

Table S8 Polymerization data for the polymerization of PO, CHO, BO, AGE, CPO and ^{t}BGE. All polymerizations were conducted using [cat]:[1,2-trans cyclohexane diol]:[epoxide] = 1:20:4000 (neat epoxide, 5 mL, 20 bar CO_2 pressure, 50 °C). Entries 1 and 2 are reproduced from references [3] and [19].

#	Epoxide (Conc. /M)	t / h	Select. CO_2 / %^{a}	Select. Poly (carbonate) / %^{b}	Activity TOF / h^{-1} ^{c}	k_{obs} / x 10^{-7} s^{-1} ^{d}	k_p / x 10^{-3} s^{-1} M^{-1} ^{e}	Poly (carbonate) molar mass M_n [$Đ$] / g mol^{-1} ^{f}
1[3]	PO (14.29)	4	> 99	98	340	400	11.20	5900 [1.10]
2[19]	CHO (9.89)	-	> 99	> 99	808	663	26.48	8900 [1.04]
3	AGE (8.49)	15	> 99	83	217	212	9.64	7100 [1.04]
4	BO (11.49)	2.5	> 99	> 99	297	222	7.93	6300 [1.04]
5	CPO (11.46)	23	> 99	> 99	58	6.22	2.22	9400 [1.04]
6	^{t}BGE	18	> 99	> 99	120	118	6.70	9000 [1.10]

$^{a}CO_2$ uptake was calculated from the ^{1}H NMR spectrum by dividing the sum of integrals for polycarbonate and cyclic carbonate against the sum of integrals for polycarbonate, cyclic carbonate, and polyether. ^{b}Poly(carbonate) selectivity was determined ^{1}H NMR spectrum by dividing the sum of integrals for polycarbonate against the sum of integrals for polycarbonate, cyclic carbonate, and polyether.^{c} Turnover frequency (TOF) was calculated by dividing the turnover number (TON) against time, where TON was determined by dividing the moles of epoxide consumed determined by comparison of the sum of integrals by ^{1}H NMR spectroscopy of poly(carbonate), cyclic carbonate and poly(ether) against mesitylene (0.25 mol %) as an internal standard. $^{d}k_{obs}$ determined as the gradient of the plot of ln[epoxide]$_t$/[epoxide]$_0$ vs time. ^{e} k_p was determined by dividing k_{obs} by [catalyst] according to the rate law deduced previously at 20 bar.[3] ^{f}Determined by GPC in THF using narrow dispersity polystyrene standards.

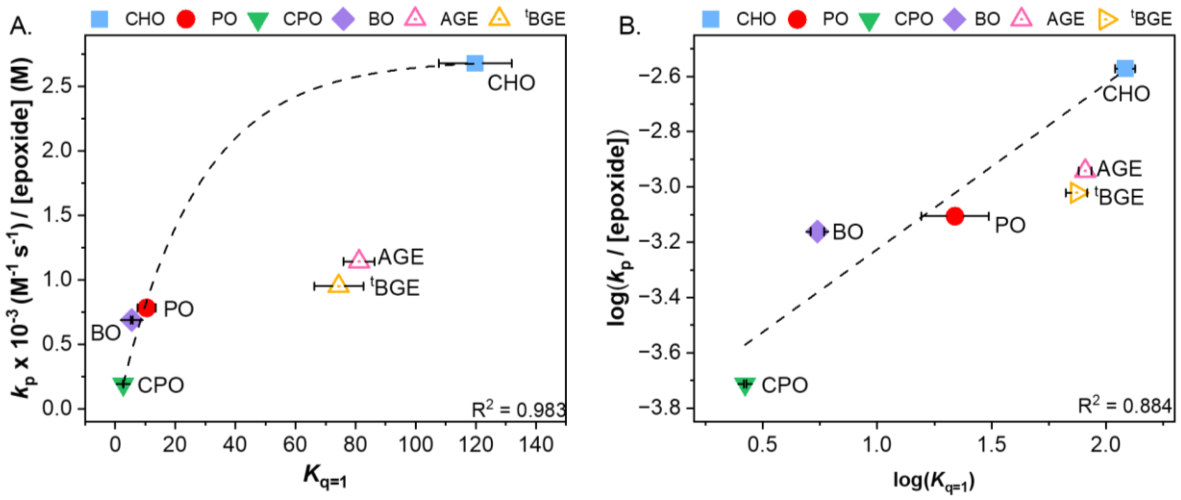

Fig. S45 A. Exponential plot of k_p normalized to the neat concentration of each epoxide vs $K_{q=1}$ for epoxide/CO_2 ROCOP catalyzed by $L_2Co(III)K(I)$, where *K* was obtained from UV-Vis spectroscopy binding experiments. Error bars on the binding constant were obtained from the non-linear fitting of the UV-Vis spectroscopy data (Table S7). **B. Linear plot of log(k_p / [epoxide]$_0$) vs log (K), showing that the observed correlation is a true LFER.** All fits are obtained from the data relating to CPO, BO, PO and CHO, AGE and ᵗBGE are excluded from the fitting as 20% cyclic carbonate were produced in the polymerization of these monomers (Table S8).

Crystallographic Details

A solvent mask has been used in the refinement of the $L_1Co(III)K(I)$ structure to remove residual electron density, which is proposed to be one disordered $CHCl_3$ molecule and half a H_2O molecule per unit cell. The solvent molecules are located across the asymmetric cell borders, making the modelling of these challenging. The solvent molecules are not coordinated to the complex and the reported structure is consistent with the previously reported one.[1]

Table S9 Selected geometric parameters for $L_1Co(II)K(I)$ and $L_1Co(III)K(I)$.

**$L_1Co(II)K(I)$ (local code: 051ke24_rint)**			
Bond length (Å)			
Co1—K1	3.4935 (10)	Co1—N1	2.081 (3)
Co1—O3	1.971 (3)	Co1—N2	2.023 (4)
Co1—O8	1.983 (3)	K1—O2	2.817 (3)
Co1—O1	2.004 (3)	O2—C1	1.245 (5)
Bond Angles (°)			
O3—Co1—K1	49.56 (8)	O1—Co1—K1	98.19 (9)
O3—Co1—O8	90.78 (11)	O1—Co1—N1	91.48 (13)
O3—Co1—O1	110.92 (12)	O1—Co1—N2	105.56 (14)
O3—Co1—N1	86.52 (13)	N1—Co1—K1	135.62 (11)
O3—Co1—N2	141.01 (14)	N2—Co1—K1	137.42 (10)
**$L_1Co(III)K(I)$ (local code: 049ke24_rint024)**			
Bond length (Å)			
Co1—K1	3.7607 (12)	Co1—O1	1.928 (3)
Co1—O5	1.907 (3)	Co1—N2	1.876 (4)
Co1—O3	1.926 (3)	Co1—N1	1.875 (4)
Co1—O10	1.921 (3)	O3—C3	1.279 (6)
Bond Angles (°)			
O5—Co1—K1	43.47 (9)	O1—Co1—K1	85.28 (10)
O5—Co1—O3	89.57 (14)	N2—Co1—K1	135.43 (13)
O5—Co1—O10	86.79 (14)	N2—Co1—O3	94.63 (15)
O5—Co1—O1	93.85 (15)	N2—Co1—O1	85.67 (15)
O3—Co1—K1	96.87 (9)	N1—Co1—K1	136.78 (13)

**L_2Co(II)K(I) (local code: 065ke25_rint0149**			
Bond length (Å)			
Co1—K1	3.4563 (13)	Co1—N1	2.095 (4)
Co1—O1	2.012 (3)	K1—O3	2.669 (4)
Co1—O3	1.966 (3)	K1—O2	2.725 (4)
Co1—O8	1.985 (3)	K1—O8	2.623 (4)
Co1—N2	2.052 (4)		
Bond Angles (°)			
O1—Co1—K1	98.92 (10)	O3—Co1—K1	50.26 (11)
O1—Co1—N2	106.40 (15)	N2—Co1—K1	135.53 (13)
O1—Co1—N1	93.81 (14)	N1—Co1—K1	135.65 (12)

Table S10 Summary of crystallographic refinement data for L_1Co(II)K(I), L_2Co(II)K(I) and L_1Co(III)K(I)

Complex	L_1Co(II)K(I)OAc	L_1Co(III)K(I)OAc$_2$	L_2Co(II)K(I)OAc
Local code	051ke24_rint	049ke24_rint024	065ke25_rint014
CCDC Deposition Number	2441770	2441771	2493075
Crystal data			
Chemical formula	$C_{24}H_{28}CoKN_2O_8$	$C_{26}H_{32}CoKN_2O_{10}$·1[$CHCl_3$]·0.5[H 2O]	$C_{24}H_{23}CoKN_2O_8$
M_r	569.50	758.94	565.37
Space group	monoclinic, $P2_1/c$	monoclinic, $P2_1/c$	monoclinic, $P2_1/n$
Temperature (K)	100	150	150
a, b, c (Å)	13.8882 (4), 17.5218 (6), 10.8779 (3)	11.7362 (4), 11.8772 (2), 24.2394 (11)	12.7806 (1), 8.4258 (1), 22.8878 (2)
β (°)	107.932 (3)	92.015 (4)	98.082 (1)
V (Å^3)	2518.51 (14)	3376.7 (2)	2440.23 (4)
Radiation type	Cu $K\alpha$	Cu $K\alpha$	Cu $K\alpha$

μ (mm^{-1})	7.27	7.76	7.50
Crystal size (mm)	0.16 × 0.13 × 0.10	0.56 × 0.05 × 0.04	0.22 × 0.14 × 0.1
Data collection			
Diffractometer	XtaLAB Synergy R, DW system, HyPix-Arc 150	SuperNova, Dual, Cu at home/near, Atlas	SuperNova, Dual, Cu at home/near, Atlas
Absorption correction	Multi-scan *CrysAlis PRO* 1.171.43.90 (Rigaku Oxford Diffraction, 2023) Empirical absorption correction using spherical harmonics, implemented in SCALE3 ABSPACK scaling algorithm.	Gaussian *CrysAlis PRO* 1.171.43.90 (Rigaku Oxford Diffraction, 2023) Numerical absorption correction based on gaussian integration over a multifaceted crystal model Empirical absorption correction using spherical harmonics, implemented in SCALE3 ABSPACK scaling algorithm.	Multi-scan *CrysAlis PRO* 1.171.43.90 (Rigaku Oxford Diffraction, 2023) Empirical absorption correction using spherical harmonics, implemented in SCALE3 ABSPACK scaling algorithm.
T_{min}, T_{max}	0.660, 1.000	0.597, 1.000	0.368, 1.000
No. of measured, independent and observed [$I > 2\sigma(I)$] reflections	28815, 5178, 4654	66103, 7047, 5303	51122, 5097, 4633
R_{int}	0.037	0.077	0.042
$(\sin \theta/\lambda)_{max}$ (Å^{-1})	0.630	0.632	0.629
Refinement			
$R[F^2 > 2\sigma(F^2)]$, $wR(F^2)$, S	0.065, 0.163, 1.06	0.076, 0.231, 1.06	0.079, 0.196, 1.08
No. of reflections	5178	7047	5097
No. of parameters	338	367	326
No. of restraints	2	0	421

$\Delta\rho_{max}$, $\Delta\rho_{min}$ (e Å^{-3})	1.23, -0.87	1.02, -0.52	1.76, -1.95

Computer programs: *CrysAlis PRO* 1.171.43.95a (Rigaku OD, 2023)[20], *SHELXT* 2018/2 (Sheldrick, 2018)[21], *SHELXL* 2018/3 (Sheldrick, 2015)[22, 23], Olex2 1.5 (Dolomanov *et al.*, 2009)[24]

References

(1) Eisenhardt, K. H. S.; Fiorentini, F.; Lindeboom, W.; Williams, C. K. Quantifying CO2 Insertion Equilibria for Low-Pressure Propene Oxide and Carbon Dioxide Ring Opening Copolymerization Catalysts. *J. Am. Chem. Soc.* **2024**, *146* (15), 10451–10464. DOI: 10.1021/jacs.3c13959.

(2) McGuire, T. M.; Ning, D.; Williams, C. K. Using Differential Scanning Calorimetry to Accelerate Polymerization Catalysis: A Toolkit for Miniaturized and Automated Kinetics Measurements. *ACS Catal.* **2025**, 6760–6771. DOI: 10.1021/acscatal.5c01758.

(3) Deacy, A. C.; Moreby, E.; Phanopoulos, A.; Williams, C. K. Co(III)/Alkali-Metal(I) Heterodinuclear Catalysts for the Ring-Opening Copolymerization of CO2 and Propylene Oxide. *J. Am. Chem. Soc.* **2020**, *142* (45), 19150–19160. DOI: 10.1021/jacs.0c07980.

(4) Darensbourg, D. J.; Chung, W.-C.; Wilson, S. J. Catalytic Coupling of Cyclopentene Oxide and CO2 Utilizing Bifunctional (salen)Co(III) and (salen)Cr(III) Catalysts: Comparative Processes Involving Binary (salen)Cr(III) Analogs. *ACS Catal.* **2013**, *3* (12), 3050–3057. DOI: 10.1021/cs4008667.

(5) Nakano, K.; Kobayashi, K.; Ohkawara, T.; Imoto, H.; Nozaki, K. Copolymerization of Epoxides with Carbon Dioxide Catalyzed by Iron–Corrole Complexes: Synthesis of a Crystalline Copolymer. *J. Am. Chem. Soc.* **2013**, *135* (23), 8456–8459. DOI: 10.1021/ja4028633.

(6) Du, P.; Li, Y.; Lu, X.-B. Chiral Organoboron-Mediated Alternating Copolymerization of meso-Epoxides with CO2. *Macromolecules* **2023**, *56* (17), 6783–6789. DOI: 10.1021/acs.macromol.3c01264.

(7) Yang, L.; Liu, S.; Zhou, Z.; Zhang, R.; Zhou, H.; Zhuo, C.; Wang, X. Aggregate Catalysts: Regulating Multimetal Cooperativity for CO2/Epoxide Copolymerization. *Macromolecules* **2024**, *57* (1), 150–161. DOI: 10.1021/acs.macromol.3c02222.

(8) Uchida, M.; Cortney, C. H.; Bustos, K.; Manzo, E.; Sauls, E.; Bouchard, J.; Fukazawa, R.; Krishnan, V. V. Discovery-Based Approach to Identify Multiple Factors That Affect the Spin State of Coordination Complexes Using the Evans NMR Method. *J. Chem. Ed.* **2023**, *100* (12), 4822–4827. DOI: 10.1021/acs.jchemed.3c00738.

(9) Gaussian 16, Revision C.01, Frisch, M. J.; Trucks, G. W.; Schlegel, H. B.; Scuseria, G. E.; Robb, M. A.; Cheeseman, J. R.; Scalmani, G.; Barone, V.; Petersson, G. A.; Nakatsuji, H.; Li, X.; Caricato, M.; Marenich, A. V.; Bloino, J.; Janesko, B. G.; Gomperts, R.; Mennucci, B.; Hratchian, H. P.; Ortiz, J. V.; Izmaylov, A. F.; Sonnenberg, J. L.; Williams-Young, D.; Ding, F.; Lipparini, F.; Egidi, F.; Goings, J.; Peng, B.; Petrone, A.; Henderson, T.; Ranasinghe, D.; Zakrzewski, V. G.; Gao, J.; Rega, N.; Zheng, G.; Liang, W.; Hada, M.; Ehara, M.; Toyota, K.; Fukuda, R.; Hasegawa, J.; Ishida, M.; Nakajima, T.; Honda, Y.; Kitao, O.; Nakai, H.; Vreven, T.; Throssell, K.; Montgomery, J. A., Jr.; Peralta, J. E.; Ogliaro, F.; Bearpark, M. J.; Heyd, J. J.; Brothers, E. N.; Kudin, K. N.; Staroverov, V. N.; Keith, T. A.; Kobayashi, R.; Normand, J.; Raghavachari, K.; Rendell, A. P.; Burant, J. C.; Iyengar, S. S.; Tomasi, J.; Cossi, M.; Millam, J. M.; Klene, M.; Adamo, C.; Cammi, R.; Ochterski, J. W.; Martin, R. L.; Morokuma, K.; Farkas, O.; Foresman, J. B.; Fox, D. J. Gaussian, Inc., Wallingford CT, 2016.

(10) Chai, J.-D.; Head-Gordon, M. Systematic optimization of long-range corrected hybrid density functionals. *J. Chem. Phys.* **2008**, *128* (8). DOI: 10.1063/1.2834918 (accessed 4/17/2025).

(11) Chai, J.-D.; Head-Gordon, M. Long-range corrected hybrid density functionals with damped atom–atom dispersion corrections. *Phys. Chem. Chem. Phys.* **2008**, *10* (44), 6615–6620, 10.1039/B810189B. DOI: 10.1039/B810189B.

(12) Grimme, S. Semiempirical GGA-type density functional constructed with a long-range dispersion correction. *J. Comput. Chem.* **2006**, *27* (15), 1787–1799. DOI: 10.1002/jcc.20495.

(13) Cossi, M.; Rega, N.; Scalmani, G.; Barone, V. Energies, structures, and electronic properties of molecules in solution with the C-PCM solvation model. *J. Comput. Chem.* **2003**, *24* (6), 669–681. DOI: 10.1002/jcc.10189.

(14) Ribeiro, R. F.; Marenich, A. V.; Cramer, C. J.; Truhlar, D. G. Use of Solution-Phase Vibrational Frequencies in Continuum Models for the Free Energy of Solvation. *J. Phys. Chem. B.* **2011**, *115* (49), 14556–14562. DOI: 10.1021/jp205508z.

(15) Li, Y.-P.; Gomes, J.; Mallikarjun Sharada, S.; Bell, A. T.; Head-Gordon, M. Improved Force-Field Parameters for QM/MM Simulations of the Energies of Adsorption for Molecules in Zeolites and a Free Rotor Correction to the Rigid Rotor Harmonic Oscillator Model for Adsorption Enthalpies. *J. Phys. Chem. B.* **2015**, *119* (4), 1840–1850. DOI: 10.1021/jp509921r.

(16) Luchini, G.; Alegre-Requena, J. V.; Funes-Ardoiz, I.; Paton, R. S. GoodVibes: automated thermochemistry for heterogeneous computational chemistry data [version 1; peer review: 2 approved with reservations]. *F1000Research* **2020**, *9(Chem Inf Sci)*, 291. DOI: 10.12688/f1000research.22758.1.

(17) Pracht, P.; Bohle, F.; Grimme, S. Automated exploration of the low-energy chemical space with fast quantum chemical methods. *Phys. Chem. Chem. Phys.* **2020**, *22* (14), 7169–7192, 10.1039/C9CP06869D. DOI: 10.1039/C9CP06869D.

(18) Bannwarth, C.; Ehlert, S.; Grimme, S. GFN2-xTB—An Accurate and Broadly Parametrized Self-Consistent Tight-Binding Quantum Chemical Method with Multipole Electrostatics and Density-Dependent Dispersion Contributions. *J. Chem. Theory Comput.* **2019**, *15* (3), 1652–1671. DOI: 10.1021/acs.jctc.8b01176.

(19) Butler, F.; Fiorentini, F.; Eisenhardt, K. H. S.; Williams, C. K. Structure-Activity Relationships for s-Block Metal/Co(III) Heterodinuclear Catalysts in Cyclohexene Oxide Ring-Opening Copolymerizations. *Angew. Chem. Int. Ed.* **2025**, *64* (12), e202422497. DOI: 10.1002/anie.202422497.

(20) *CrysAlis PRO*; Rigaku Oxford Diffraction Ltd, Yarnton, Oxfordshire, England.: 2020. (accessed 07/04/2025).

(21) Sheldrick, I. U. a. G. M. An introduction to experimental phasing of macromolecules illustrated by SHELX; new autotracing features. *Acta Cryst.* **2018**, (D74), 106–116.

(22) Sheldrick, G. M. SHELXT – Integrated space-group and crystal-structure determination. *Acta Cryst. A* **2014**, *71*, 3–8. DOI: doi.org/10.1107/S2053273314026370.

(23) Sheldrick, G. M. Crystal structure refinement with SHELXL. *Acta Cryst. C* **2015**, *71*, 3–8.

(24) Dolomanov, O. V., Bourhis, L. J., Gildea, R. J., Howard, J. A. K. & Puschmann, H. . OLEX2: a complete structure solution, refinement and analysis program. *J. Appl. Cryst.* **2009**, *41*, 339–341.
